# Precise and broad scope genome editing based on high-specificity Cas9 nickases

**DOI:** 10.1093/nar/gkaa1236

**Published:** 2021-01-04

**Authors:** Qian Wang, Jin Liu, Josephine M Janssen, Marie Le Bouteiller, Richard L Frock, Manuel A F V Gonçalves

**Affiliations:** Department of Cell and Chemical Biology, Leiden University Medical Center, Einthovenweg 20, 2333 ZC Leiden, The Netherlands; Department of Cell and Chemical Biology, Leiden University Medical Center, Einthovenweg 20, 2333 ZC Leiden, The Netherlands; Department of Cell and Chemical Biology, Leiden University Medical Center, Einthovenweg 20, 2333 ZC Leiden, The Netherlands; Department of Radiation Oncology, Division of Radiation and Cancer Biology, Stanford University School of Medicine, 269 Campus Dr. Stanford, CA 94305, USA; Department of Radiation Oncology, Division of Radiation and Cancer Biology, Stanford University School of Medicine, 269 Campus Dr. Stanford, CA 94305, USA; Department of Cell and Chemical Biology, Leiden University Medical Center, Einthovenweg 20, 2333 ZC Leiden, The Netherlands

## Abstract

RNA-guided nucleases (RGNs) based on CRISPR systems permit installing short and large edits within eukaryotic genomes. However, precise genome editing is often hindered due to nuclease off-target activities and the multiple-copy character of the vast majority of chromosomal sequences. Dual nicking RGNs and high-specificity RGNs both exhibit low off-target activities. Here, we report that high-specificity Cas9 nucleases are convertible into nicking Cas9^D10A^ variants whose precision is superior to that of the commonly used Cas9^D10A^ nickase. Dual nicking RGNs based on a selected group of these Cas9^D10A^ variants can yield gene knockouts and gene knock-ins at frequencies similar to or higher than those achieved by their conventional counterparts. Moreover, high-specificity dual nicking RGNs are capable of distinguishing highly similar sequences by ‘tiptoeing’ over pre-existing single base-pair polymorphisms. Finally, high-specificity RNA-guided nicking complexes generally preserve genomic integrity, as demonstrated by unbiased genome-wide high-throughput sequencing assays. Thus, in addition to substantially enlarging the Cas9 nickase toolkit, we demonstrate the feasibility in expanding the range and precision of DNA knockout and knock-in procedures. The herein introduced tools and multi-tier high-specificity genome editing strategies might be particularly beneficial whenever predictability and/or safety of genetic manipulations are paramount.

## INTRODUCTION

RNA-guided nucleases (RGNs) based on prokaryotic CRISPR–Cas9 adaptive immune systems consist of ribonucleoprotein complexes made of single guide RNAs (gRNAs) and Cas9 nucleases (1). RGNs are programmable nucleases in that they can be tailored to cleave specific DNA sequences whose recognition involves sequential protein–DNA and RNA–DNA interactions. Firstly, the Cas9 component binds to a so-called protospacer adjacent motif (PAM) on the DNA (2). The PAM of the prototypic *Streptococcus pyogenes* Cas9 (SpCas9) nuclease and that of its orthologue *Staphylococcus aureus* Cas9 (SaCas9) nuclease, reads NGG and NNGRRT, respectively (3,4). Secondly, hybridization of the 5′ end of the gRNA (spacer) to a normally 20 nucleotide-long sequence (protospacer) located next to the PAM ultimately triggers double-stranded DNA break (DSB) formation through the allosteric activation of the two Cas9 nuclease domains, i.e. RuvC-like and HNH (1). Hence, RGNs bypass the need for protein engineering owing to their RNA-based programmability and, as such, constitute versatile and powerful tools for changing specific nucleotide sequences amidst large eukaryotic genomes (1,5). Commonly, such genome editing maneuvers yield gene knockouts and, in the presence of exogenous donor DNA, gene knock-ins resulting from non-homologous end joining (NHEJ) and homology-directed repair (HDR) of site-specific DSBs, respectively (1,5).

Despite the far-reaching appeal of RGN technologies, major concerns regarding their use are, however, off-target DNA cleavage and associated collateral effects, e.g. chromosomal sequence disruptions and translocations (6–12). Off-target activities result from the fact that, often, RGNs remain cleaving-proficient even when several mismatches exist between gRNA and genomic sequence(s). This is especially so if the mismatches locate distally to the PAM (7–9). Moreover, although to a lesser degree than NGG, certain non-canonical PAMs (e.g. NAG) can also be engaged by *S. pyogenes* Cas9 and lead to off-target DSB formation when located next to sequences fully or partially complementary to the gRNA spacer (7–9,12–14).

RGN off-target activities have prompted an increasing number of Cas9 mutagenesis screens based on rational design and directed evolution principles whose results include an expanding portfolio of Cas9 variants with enhanced target site specificities (15). A parallel, broadly applicable, approach for reducing off-target activities involves using nicking RGN (nRGN) pairs containing sequence- and strand-specific Cas9 nucleases (nickases) generated by disabling either the RuvC-like (Cas9^D10A^) or the HNH (Cas9^H840A^) domains (3,16,17). The simultaneous induction of single-stranded DNA breaks (SSBs) at offset positions in opposite target DNA chain by pairs of these nicking RGNs (dual nRGNs) yields a targeted DSB (18,19). Crucially, SSBs made at off-target sites by individual dual nRGN pair members are mostly repaired through conservative, non-mutagenic, DNA repair processes (20,21). Notably, when compared to regular RGNs containing Cas9, dual nRGNs harboring Cas9^D10A^ offer a higher target-site selection density and, hence, wider genomic space coverage. This follows from the fact that the effective spacing separating the bipartite target sites of dual nRGNs is relatively broad (up to ∼100 bp) widening the range for locating suitable PAMs (18,19). Moreover, dual nRGNs containing Cas9^D10A^ can sometimes induce higher target DNA cleaving activities when compared to their corresponding monomeric RGNs (22). Presumably, this results from the fact that such dual nRNGs bypass the need for a functional RuvC-like domain, which of the two SpCas9 nuclease domains, seems to be the least catalytically active in mammalian cells (22).

In this study, we start by investigating whether a representative panel of SpCas9 nucleases with enhanced specificities, i.e. SpCas9-KA (23), SpCas9-KARA (23), eSpCas9(1.1) (23), Sniper-Cas9 (24), SpCas9-HF1 (25), evoCas9 (26) and xCas9-3.7 (27), are convertible into functional nicking forms. In these experiments, the activities and specificities of the respective nRGNs were compared with those containing the conventional Cas9^D10A^ nickase. Subsequently, we asked whether these new enzymes are operational as dual nRGNs for triggering gene knockouts and gene knock-ins in human cells, including induced pluripotent stem cells (iPSCs). We report that high-specificity SpCas9 proteins vary greatly in their permissiveness to the incorporation of the RuvC-disabling D10A mutation. Indeed, the phosphodiester bond cleaving efficiencies achieved by these RNA-programmable nickases, in their single and dual nRGN formats, varies from lower to higher than those obtained via their respective, unmodified, Cas9^D10A^-containing counterparts. Importantly, the identified high-activity Cas9^D10A^ nickases endow single and dual nRGNs with specificities that are superior to those conferred by the unmodified Cas9^D10A^ protein.

## MATERIALS AND METHODS

### Cells

Human cervix carcinoma (HeLa) cells and human embryonic kidney 293T (HEK293T) cells (both from American Type Culture Collection) were cultured in Dulbecco's modified Eagle's medium (DMEM; Thermo Fisher Scientific; Cat. No.: 41966029) supplemented with 5% and 10% fetal bovine serum ultra-low endotoxin (FBS; Biowest; Cat. No.: S1860500), respectively. The generation and characterization of H2AX::mCherry^+^, TURQ2 and H27 cells were described elsewhere (14,28,29). All these reporter HeLa cell-derived cell lines were maintained in DMEM containing 5% FBS.

The human iPSCs used in this study (LUMC0020iCTRL06) were generated and characterized elsewhere (28). The iPSCs were maintained in feeder-free Essential 8 Medium (E8; Thermo Fisher Scientific; Cat. No.: A1517001) supplemented with 25 U ml^−1^ penicillin and 25 μg ml^−1^ of streptomycin (Thermo Fisher Scientific; Cat. No.: 15140122). The cells were passaged as small clumps using 0.5 mM ethylenediaminetetraacetic acid (EDTA) (Invitrogen; Cat. No: 15575020) diluted 1:1000 in Dulbecco's phosphate-buffered saline (DPBS; Thermo Fisher Scientific; Cat. No.: 14190094) every three to four days and were re-plated in wells of six-well plates (Greiner Bio-One; Cat. No.: 662160) containing E8 medium supplemented with a 1:200 dilution of RevitaCell (Thermo Fisher Scientific; Cat. No.: A2644501). All the cell culture vessels used for iPSCs culture in this work were coated with Vitronectin Recombinant Human Protein (VTN-N; Thermo Fisher Scientific; Cat. No.: A14700) diluted 1:100 to a final concentration of 5 ng ml^−1^ in DPBS for at least 1 h at room temperature (RT).

The various cell types were kept at 37°C in a humidified-air 10% CO_2_ atmosphere except for iPSCs, which were instead maintained in a humidified-air 5% CO_2_ atmosphere. The cells used in this work were tested for the absence of mycoplasma.

### Recombinant DNA

The isogenic expression plasmids containing the open reading frames of the SpCas9 nucleases and SpCas9 nickases under the control of the same hybrid CAG promoter and rabbit *β-globin* polyadenylation signal, were assembled on the basis of the indicated previously published constructs and BB36_pCAG.Cas9eSp(1.1)-D10A.bGHpA, AL65_pEX-A128.partialCas9-eSp(1.1).K1003-R1060, AL66_pEX-A128.partialCas9-eSp(1.1).K1003, BA59_pUC57.start-Cas9-HF1-D10A, AL68_pEX-A258.Cas9-evo(partial) and BA16_pU.CAG.dSaCas9.rBGpA. The codes and names of the expression plasmids encoding SpCas9 nucleases and nickases generated in this study are gathered in Supplementary Table S1. The annotated maps and nucleotide sequences of BB36_pCAG.Cas9eSp(1.1)-D10A.bGHpA, AL65_pEX-A128.partialCas9-eSp(1.1).K1003-R1060, AL66_pEX-A128.partialCas9-eSp(1.1).K1003, BA59_pUC57.start-Cas9-HF1-D10A, AL68_pEX-A258.Cas9-evo(partial) and BA16_pU.CAG.dSaCas9.rBGpA are available in pages 1–14 of the Supplementary Information. The amino acid sequences of nickases encoded by AB65_pU.CAG.Cas9-D10A.rBGpA (14), AP76_pU.CAG.Cas9-D10A-K848A.rBGpA, AP70_pU.CAG.Cas9-D10A-K848A-R1060A.rBGpA, AA69_pU.CAG.Cas9-eSp(1.1)-D10A.rBGpA.2NLS, AE70_pU.CAG.SniperCas9-D10A.rBGpA, BB37_pU.CAG.Cas9-HF1-D10A.rBGpA, AP74_pU.CAG.Cas9-evo-D10A.rBGpA and AT85_pU.CAG.xCas9-3.7-D10A.rBGpA are depicted in pages 15–22 of the Supplementary Information. Constructs AW01_pU.CAG.Cas9-eSp(1.1).rBGpA (30) and BB36_pCAG.Cas9eSp(1.1)-D10A.bGHpA were digested with BshTI and Eco32I. Subsequently, the 7378-bp backbone fragment from AW01_pU.CAG.Cas9-eSp(1.1).rBGpA (30) and the 1982-bp insert fragment from BB36_pCAG.Cas9eSp(1.1)-D10A.bGHpA were extracted from agarose gel and ligated together, leading to the generation of construct AA69_pU.CAG.Cas9-eSp(1.1)-D10A.rBGpA.2NLS encoding eSpCas9(1.1)^D10A^. Next, AW01_pU.CAG.Cas9-eSp(1.1).rBGpA (30) and AA69_pU.CAG.Cas9-eSp(1.1)-D10A.rBGpA.2NLS were digested with Eco72I and BsmI, after which, the 8509-bp backbone fragments were isolated from agarose gel and dephosphorylated with FastAP (Thermo Fisher Scientific; Cat. No.: EF0651) for 1 h at 37°C according to the specifications of the manufacturer. The 851-bp insert fragments encoding SpCas9-KA and SpCas9-KARA were extracted from agarose gel after digesting AL65_pEX-A128.partialCas9-eSp(1.1).K1003-R1060 and AL66_pEX-A128.partialCas9-eSp(1.1).K1003 with Eco72I and BsmI. Subsequently, the resulting insert fragments were ligated to the dephosphorylated vector backbone from AW01_pU.CAG.Cas9-eSp(1.1).rBGpA (30) or that from AA69_pU.CAG.Cas9-eSp(1.1)-D10A.rBGpA.2NLS. These maneuvers led to the assembly of expression constructs AP75_pU.CAG.Cas9-K848A.rBGpA, AP76_pU.CAG.Cas9-D10A-K848A.rBGpA, AP69_pU.CAG.Cas9-K848A-R1060A.rBGpA and AP70_pU.CAG.Cas9-D10A-K848A-R1060A.rBGpA encoding SpCas9-KA, SpCas9-KA^D10A^, SpCas9-KARA and SpCas9-KARA^D10A^, respectively. To generate expression plasmids encoding Sniper-Cas9 and Sniper-Cas9^D10A^, constructs AV62_pU.CAG.Cas9.rBGpA (30) and AB65_pU.CAG.Cas9-D10A.rBGpA (14) were digested with SdaI and Eco72I. The resulting 6673-bp backbone fragments were then extracted from agarose gel and dephosphorylated as above-indicated. Next, plasmid AV72_pCMV.Sniper-Cas9.bGHpA (Addgene plasmid #113912) was digested with SdaI and Eco72I, after which, the 2542-bp insert fragment was ligated to the dephosphorylated vector backbones from AV62_pU.CAG.Cas9.rBGpA (30) and AB65_pU.CAG.Cas9-D10A.rBGpA (14), yielding constructs AE69_pU.CAG.SniperCas9.rBGpA and AE70_pU.CAG.SniperCas9-D10A.rBGpA, respectively. For generating the construct encoding SpCas9-HF1^D10A^, plasmids AV64_pU.CAG.Cas9-HF1.rBGpA (30) and BA59_pUC57.start-Cas9-HF1-D10A were digested with SacI and BstZ17I. Subsequently, the 9039-bp backbone fragment from AV64_pU.CAG.Cas9-HF1.rBGpA (30) and the 261-bp insert fragment from BA59_pUC57.start-Cas9-HF1-D10A were isolated from agarose gel and ligated together, leading to the expression construct BB37_pU.CAG.Cas9-HF1-D10A.rBGpA. To assemble expression plasmids encoding evoCas9 and evoCas9^D10A^, constructs AV62_pU.CAG.Cas9.rBGpA (30) and AB65_pU.CAG.Cas9-D10A.rBGpA (14) were digested with SalI and BamHI and, after agarose gel extraction, the 7750-bp backbone fragments were dephosphorylated. Next, construct AL68_pEX-A258.Cas9-evo(partial) was digested with SalI and BamHI, after which, the 1465-bp insert fragment was isolated from agarose gel and ligated to the dephosphorylated vector backbones from AV62_pU.CAG.Cas9.rBGpA (30) and AB65_pU.CAG.Cas9-D10A.rBGpA (14), resulting in constructs AP73_pU.CAG.Cas9-evo.rBGpA and AP74_pU.CAG.Cas9-evo-D10A.rBGpA, respectively. To generate expression plasmids encoding xCas9-3.6, xCas9-3.6^D10A^, xCas9-3.7 and xCas9-3.7^D10A^, AV62_pU.CAG.Cas9.rBGpA (30) and AB65_pU.CAG.Cas9-D10A.rBGpA (14) were digested with SdaI and BshTI and the 5309-bp backbone fragments were then extracted from agarose gel and dephosphorylated. In parallel, AE65_pCMV.xCas9-3.6.HSV-TKpA (Addgene plasmid #108384) and AE66_pCMV.xCas9-3.7.HSV-TKpA (Addgene plasmid #108379) were digested with SdaI and BshTI and the 3908-bp insert fragments were then isolated from agarose gel and ligated to the dephosphorylated vector backbone from AV62_pU.CAG.Cas9.rBGpA (30) or that from AB65_pU.CAG.Cas9-D10A.rBGpA (14). These manoeuvres led to the assembly of AT82_pU.CAG.xCas9-3.6.rBGpA, AT83_pU.CAG.xCas9-3.6-D10A.rBGpA, AT84_pU.CAG.xCas9-3.7.rBGpA, and AT85_pU.CAG.xCas9-3.7-D10A.rBGpA encoding xCas9-3.6, xCas9-3.6^D10A^, xCas9-3.7 and xCas9-3.7^D10A^, respectively. The generation of the construct expressing nicking SaCas9^D10A^ was carried out as follows. Plasmids BA15_pCAG.SaCas9.rBGpA (31) and BA16_pU.CAG.dSaCas9.rBGpA were digested with BcuI and Kpn2I, after which, the 5063-bp backbone and 3316-bp insert fragments, respectively, were isolated from agarose gel and ligated to each other yielding BA31_pU.CAG.SaCas9-D10A.rBGpA.

The expression plasmids coding for gRNAs used in this work were assembled by inserting annealed oligonucleotide pairs indicated in Supplementary Table S2 into BveI-digested AY56_pUCBM21.U6.opt-sgRNA.Bvel-stuffer (32). AV85_pSa-gRAG1.1 (14) and AM51_pUCBM21.U6.gRNAI-SceI.1 (30), encoding *RAG1*-specific Sa-gRNA1.1 and an irrelevant, non-targeting gRNA, respectively, have been described previously (14,30).

### Cell transfections

With the exception of iPSCs, all other cell types were seeded in the cell culture vessels indicated in Supplementary Tables S3–S26. At ∼16–24 h after seeding, the cells were transfected with the aid of 1 mg ml^−1^ 25 kDa linear polyethyleneimine (PEI, Polysciences) solution (pH 7.4). The cell numbers, the amounts of PEI, DNA (in ng) and 150 mM NaCl (in μl) as well as the compositions of each DNA mixture corresponding to the different transfection reactions are specified in Supplementary Tables S3–S26. Prior to transfection the plasmids were first diluted in 150 mM NaCl (Merck), after which, the appropriate amount of the PEI solution was added to each of the transfection reactions. After vigorously vortexing for about 10 s, the transfection mixtures were incubated for 15 min at RT to let PEI–DNA complexes form. The resulting transfection mixtures were then directly added into the culture media of the target cells and, after 6 h, the transfection media were substituted by regular culture media.

The transfections of iPSCs were done by using Lipofectamine Stem Transfection Reagent (Thermo Fisher Scientific, Cat. No.: STEM00003) according to the manufacturer's protocols. In brief, cells were seeded in wells of 24-well plates coated with Vitronectin with the culture media refreshed at least 2 h prior to transfection. The cell numbers, the amounts of Lipofectamine Stem Transfection Reagent (in μl), DNA (in ng) as well as the compositions of each of the DNA mixtures corresponding to the different transfection reactions are specified in Supplementary Table S27. The plasmid mixtures and the appropriate amounts of Lipofectamine Stem Transfection Reagent were diluted in 25 μl of Opti-MEM medium (Gibco; Cat. No.: 31985-047) in 1.5-ml sterile Eppendorf tubes. After mixing, by gently pipetting, the resulting transfection reactions were incubated at RT for 10 min and were then directly added into the culture media of the target iPSCs. The transfection media were replaced with regular iPSC culture medium 24 h post-transfection.

### Flow cytometry

**Figure 1.**
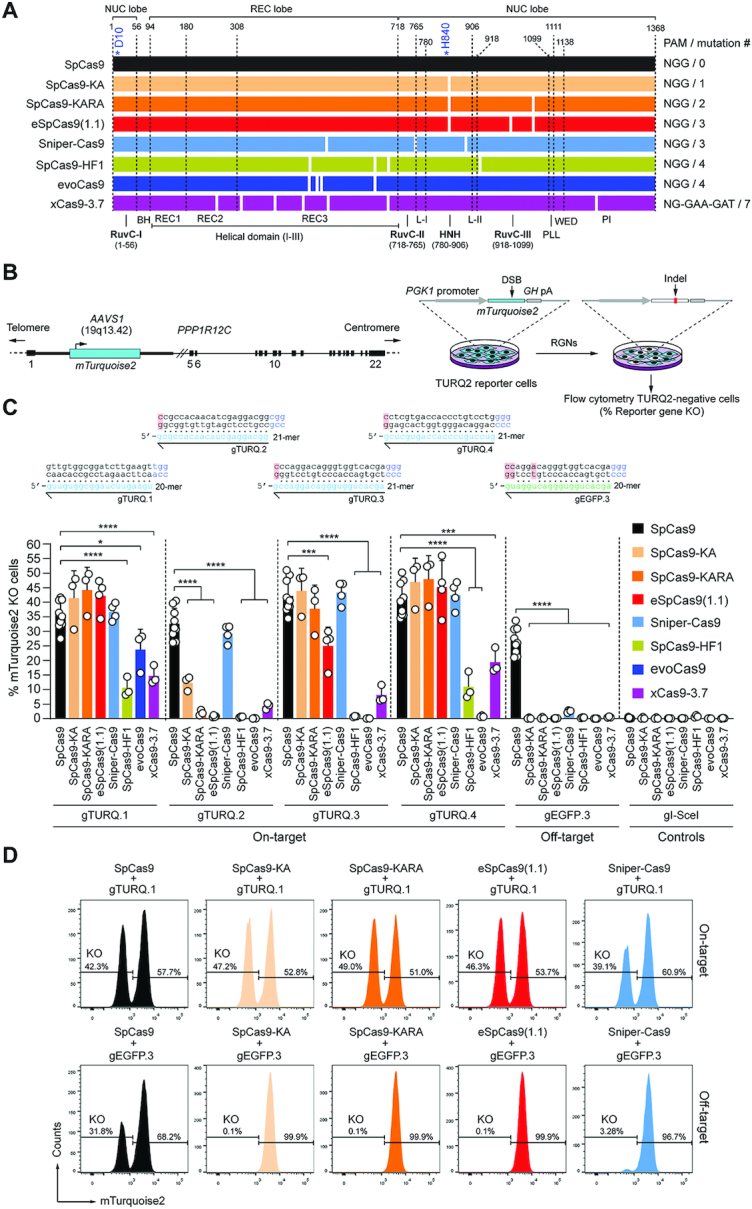
Comparing the activity and specificity of RGNs based on SpCas9 or SpCas9 variants. (**A**) Schematics of nucleases derived from the *S. Pyogenes* type II CRISPR system. Protein domains and mutations (white bars) are indicated. HNH, histidine-asparagine-histidine nuclease domain; RuvC, RNase H-like fold nuclease domain formed by tripartite assembly of RuvC-I, -II and -III. The HNH and RuvC domains in the nuclease lobe digest the target and non-target DNA strands, respectively. L-I and L-II, linker region I and II, respectively. Numerals correspond to the amino acid positions delimiting the various protein domains and motifs. BH, Arginine-rich bridge helix that connects the NUC and REC lobes; CTD, C-terminal domain in which the PAM-interacting motif (PI) is lodged; NUC and REC, nuclease and recognition lobes, respectively; PLL, phosphate lock loop. Asterisks mark residues D10 and H840 crucial for RuvC and HNH catalytic activities, respectively. (**B**) Gene knockout assays. TURQ2 cells contain an *mTurquoise2* transgene at intron 1 of *PPP1R12C* (*AAVS1* locus). Small insertions and deletions (indels) resulting from the action of programmable nucleases and NHEJ pathways at *mTurquoise2* yield gene knockouts quantifiable by flow cytometry. (**C**) Determining RGN activities. TURQ2 cells were transfected with plasmids expressing the indicated RGN components. The gRNAs gTURQ.1 through gTURQ.4 have spacers fully complementary to *mTurquoise2* sequences (on-target); *EGFP*-specific gEGFP.3 has a spacer with mismatches to a *mTurquoise2* sequence (off-target). The non-targeting gRNA gI-SceI was used as a negative control. Non-hybridizing DNA-gRNA bases are highlighted in red. Gene knockout frequencies were determined at 10 days post-transfection through flow cytometry of mTurquoise2-negative cells. Data are presented as mean ± S.D. of at least three independent biological replicates. Significant differences between datasets were calculated with one-way ANOVA followed by Dunnett's test for multiple comparisons; *0.01 < *P* < 0.05; ***0.0001 < *P* < 0.001; ****P < 0.0001. (**D**) Examples of gene knockout datasets. Histograms corresponding to TURQ2 cell populations subjected to RGNs with spacers complementary and partially complementary to a target sequence (top and bottom panels, respectively).

Gene knockout frequencies in transfected cell populations were determined by flow cytometry of reporter-negative cells at 10 days post-transfection and, with the exception of the experiments presented in Figure 1 and Supplementary Figure S1, were normalized for initial transfection efficiencies on a per sample basis by reporter-directed flow cytometry at 3 days post-transfection. The flow cytometry analyses were carried out by using a BD LSR II flow cytometer (BD Biosciences). In brief, cells were trypsinied, washed with PBS and resuspended in PBS supplemented with 0.5% bovine serum albumin (BSA) and 2 mM EDTA (pH 8.0). Parental non-transfected cells were used as negative controls to set background fluorescence. At least 10 000 viable single cells were acquired per sample. Data were analyzed with the aid of FlowJo 10.5.0 software (Tree Star).

### Western blotting

Cells were lysed with Laemmli buffer consisting of 8.0% glycerol, 3% sodium dodecyl sulfate (SDS) and 200 mM Tris–HCl (pH 6.8), followed by boiling at 100°C for 5 min. Protein concentrations were measured by a DC™ protein assay kit (Bio-Rad; Cat. No.: 5000111) according to the manufacturer's instructions. Equal amounts of proteins were loaded and separated by SDS-polyacrylamide gel electrophoresis (SDS-PAGE). Afterwards, the resolved proteins were transferred onto 45-μm polyvinylidene difluoride (PVDF) membrane (Merck Millipore; Cat. No.: IPVH00010). Next, 5% non-fat dry milk dissolved in Tris-buffered saline with 0.1% Tween 20 (TBST) was used to block the membrane at RT for 1 h. Membranes were incubated overnight at 4°C with the respective primary antibodies recognizing *S. pyogenes* Cas9 (Abcam; Cat. No.: ab191468), α/β Tubulin (Cell Signaling; Cat. No.: 2148), and GAPDH (Merck Millipore; Cat. No.: MAB374) diluted 1:1000 in TBST supplemented with 5% BSA. Subsequently, the membranes were washed with TBST thrice and probed with secondary antibodies specific for mouse IgG (Sigma-Aldrich; Cat. No.: NA931V) or rabbit IgG (Cell Signaling; Cat. No.: 7074S) diluted 1:5000 in TBST containing 1% non-fat dry milk at RT for 2 h. Clarity™ Western ECL Substrate (Bio-Rad; Cat. No.: 1705060) was applied for signal detection using the ChemiDoc Imaging System (Bio-Rad; Cat. No.: 17001402).

### Testing gene-editing tools at alternate chromatin states

Cultures of HEK.EGFP^TetO.KRAB^ cells (30), were either not treated or treated with doxycycline (Dox) at a final concentration of 200 ng ml^–1^ starting 7 days prior to transfection (Supplementary Table S19). After a sub-culture period of 10 days, HEK.EGFP^TetO.KRAB^ cells that were kept in the presence or absence of Dox (200 ng ml^–1^), were incubated for an additional 7-day period with Dox (200 ng ml^-1^), after which, the frequencies of EGFP-negative cells were determined by flow cytometry.

### Target-site genotyping assays

Genotyping assays based on the mismatch-sensing T7 endonuclease I (T7EI), were performed for the assessment of NHEJ-derived indel formation at target sequences. In brief, genomic DNA was extracted by using the DNeasy Blood & Tissue Kit (Qiagen; Cat. No.: 69506) according to the manufacturer's instructions. Next, the various target sites were amplified with the aid of the primers listed in Supplementary Tables S28 and S29. The cycling conditions and PCR mixture compositions used are specified in Supplementary Tables S28 and S30–S33. The resulting amplicons were subjected to cycles of denaturation and reannealing to form heteroduplexes using the thermocycling parameters indicated in Supplementary Table S34. Subsequently, 10 μl of reannealed samples were treated with 0.5 μl (5U) of T7EI (New England Biolabs; Cat. No.: M0302) at 37°C for 15 min and were analysed by agarose gel electrophoresis. Parallel samples of reannealed amplicons not treated with T7EI served as negative controls. After electrophoresis, untreated and T7EI-treated amplicons were detected by using the Gel-Doc XR+ system and the ImageLab 4.1 software (both from Bio-Rad).

### Clonal analysis for assessing gene knock-ins at *OCT4* in HeLa cells

HeLa cells were transfected as indicated in Supplementary Table S24. At 3 days post-transfection, the cells were transferred into wells of six-well plates (Greiner Bio-One) and were subsequently exposed to 1 μg ml^−1^ puromycin (Invitrogen, Cat. No.: A11138-03) for 7 days. The resulting puromycin-resistant HeLa clones were identified through colony-formation assays using standard Giemsa or Crystal violet staining protocols. In addition, parallel cultures of puromycin-resistant HeLa cell populations were seeded at a density of 0.3 cells per well in wells of 96-well plates (Greiner Bio-One). The resulting single cell-derived clones were then sub-cultured for ∼3 weeks in DMEM supplemented with 5% FBS, 1 μg ml^−1^ puromycin, 50 nM α-thioglycerol (Sigma-Aldrich; Cat. No.: M6145) and 0.02 nM bathocuproinedisulfonic acid disodium salt (Sigma-Aldrich; Cat. No.: B1125). Subsequently, genomic DNA of randomly collected single cell-derived clones was extracted and analysed by junction PCR using Phire™ Tissue Direct PCR Master Mix (Thermo Fisher Scientific, Cat. No.: F-107L) according to the manufacturer's protocols. The PCR primer pairs, composition of the PCR mixtures and cycling parameters are specified in Supplementary Tables S35 and S36, respectively.

### Quantification of *OCT4* gene targeting frequencies in iPSCs

The transfection of iPSCs was carried out as indicated under ‘Cell transfections’ and in Supplementary Table S27. At 2 days post-transfection, the iPSCs were transferred into new wells of 24-well plates (Greiner Bio-One) and were subsequently expanded into wells of six-well plates (Greiner Bio-One) for 5–7 days in the presence of 0.5 μg ml^−1^ puromycin in E8 Medium containing 25 U ml^−1^ penicillin and 25 μg ml^−1^ streptomycin. The resulting puromycin-resistant iPSC colonies were identified through colony-formation assays using the leukocyte AP kit and protocol (Sigma-Aldrich; Cat. No.: 86R-1KT). In addition, parallel cultures of puromycin-resistant iPSC populations were further expanded for quantification of *OCT4* gene targeting frequencies. In brief, puromycin-resistant iPSC populations resulting from the different *OCT4* gene targeting strategies were reseeded in wells of 24-well plates at a density of 40 000 cells per well. The next day, a lentiviral vector expressing the bacteriophage P1 Cre recombinase (LV.Cre) (14) was added to the target iPSCs at a multiplicity-of-infection (MOI) of 20 viral particles per cell. After a 5-day sub-culture period, the frequency of iPSCs expressing OCT4::EGFP, assembled via Cre-mediated recombination, was measured by flow cytometry.

### Confocal immunofluorescence microscopy

Cells were fixed in 4% paraformaldehyde (PFA) and were permeabilized in 0.5% Triton X-100 in tris-buffered saline (TBS) pH 7.6 (50 mM Tris–HCl pH 7.6; 150 mM NaCl) at RT for 10 min, after three washes with 0.1% Triton X-100 in TBS (TBST). A blocking solution consisting of TBS, 0.1% Triton X-100, 2% BSA and 0.1% sodium azide was applied to block non-specific antibody binding for 1 h at RT. Next, the cells were incubated with the primary antibodies indicated in Supplementary Table S37, diluted in blocking solution for 1 h at RT. The specimens were subsequently subjected to three washes with TBST and the target antigens were probed with fluorochrome-conjugated secondary antibodies diluted in blocking solution for 1 h in the dark at RT (Supplementary Table S37). Finally, ProLong Gold Antifade Mounting reagent containing DAPI (Thermo Fisher Scientific; Cat. No.: P36931) was used for mounting samples after three washes with TBST. The fluorescence images were captured with the aid of an upright Leica SP8 confocal microscope (Leica Microsystems) equipped with Leica hybrid detectors, HyD (Leica Microsystems) and were analyzed using LAS X software.

### Spontaneous differentiation of iPSCs

OCT4::EGFP^+^ iPSC populations were dissociated into large cell clumps by scrapping after incubating them in PBS/EDTA for 1 min at 37°C. The cell clumps were then cultured in suspension at 37°C for 24 h on low-attachment plates containing culture media E8. Next, the cell clumps were seeded on glass coverslips coated with Vitronectin in culture media supplemented with Revitacell. After 2 days in culture, the medium was changed to differentiation medium DMEM/F12 (Gibco; Cat. No. 31331-028) containing 20% FBS. The differentiation medium was replenished every 2–3 days during the following 3 weeks. Immunofluorescence staining was carried out to detect the markers for mesoderm, ectoderm and endoderm (Supplementary Table S37). The targeted markers for these embryonic germ layers were, α-smooth muscle actin (α-SMA), tubulin β3 class III (TUBB3) and α-fetoprotein (AFP), respectively.

### Preparation of genomic DNA for orthogonal HTGTS analysis

The isolation of genomic DNA used for orthogonal HTGTS analysis was detailed elsewhere (14). In brief, HEK293T cells transfected as indicated in Supplementary Table S26, were collected at 36 h post-transfection and were resuspended in freshly prepared lysis buffer containing 200 mM NaCl, 10 mM Tris–HCl (pH 7.4), 2 mM EDTA (pH 8.0), 0.2% SDS and 200 ng ml^−1^ proteinase K (Thermo Fisher Scientific; Cat. No.: #EO0491). After overnight incubation at 56°C, genomic DNA was precipitated by adding isopropanol to a final concentration of 50% and then washed with 1 ml of 70% ethanol. After centrifugation at 13 000 × g for 5 min at 4°C, genomic DNA pellets were dissolved in TE buffer (10 mM Tris–HCl pH 8.0 and 1 mM EDTA pH 8.0) for at least 2 h at 56°C. The assessment of bait and prey chromosomal DNA breaks at *RAG1* and *VEGFA* alleles in the transfected HEK293T cell populations was done using T7EI-based genotyping assays. To this end, the *RAG1* and *VEGFA* target regions were PCR-amplified with KOD Hot Start DNA Polymerase (Merck Millipore; Cat. No.: 71086-3) and GoTaq G2 Flexi DNA Polymerase (Promega; Cat. No.: M7805) by using the PCR mixtures indicated in Supplementary Tables S32 and S33, respectively. The PCR primer pairs and cycling parameters are specified in Supplementary Tables S29 and S31, respectively. Subsequently, the amplicons were subjected to T7EI treatments for the detection of indels at *RAG1* and *VEGFA* loci.

### Assessing genome-wide off-target effects through orthogonal HTGTS analyses

The orthogonal HTGTS analyses on genomic DNA samples extracted from transfected HEK293T cells were performed in a blind fashion. The reagents and protocols used in HTGTS, including the orthogonal HTGTS assay, have been detailed elsewhere (12,14,33). In this work, however, prey/bait sequence alignments were performed against human genome assembly hg38 instead of hg19. In brief, 25-μg genomic DNA samples were sheared in a Bioruptor (Diagenode) with a circulating temperature of 4°C using a low-power setting, i.e. 2 × 30 s pulses intercalated by a cooldown period of 60 s. The biotinylated RAG1A/B-F1 primer (12) was used for LAM-PCR (33). Prior to the ligation of bridge adapters (12,33), the LAM-PCR ssDNA products were purified using streptavidin-coated magnetic beads (ThermoFisher Scientific; Cat. No.: 65002). Barcoded RAG1A/B-F2 I5 and AP2 I7 primers (12) and primers P5–I5 and P7–I7 primers (33) were applied for the nested PCR and final PCR, respectively. The PCR products ranging in size from 500 bp to 1 kb were subsequently purified after agarose gel electrophoresis (Qiagen; Cat. No.: 28706). The Phusion polymerase (ThermoFisher Scientific; Cat. No.: F530L) was used for the synthesis of the various amplicons with the blocking enzyme step being omitted. The HTGTS deep sequencing libraries were run on a Bioanalyzer (Agilent 2100) prior to 250-bp paired end MiSeq sequencing (Illumina; Cat. No.: MS-102-2003). The resulting pooled sequence reads were demultiplexed and trimmed using the selected molecular barcodes and adapter sequences. Finally, each read library was subjected to (i) bait/prey sequence alignments to the human genome assembly hg38, (ii) filtering and (iii) post-pipeline analysis as specified elsewhere (33). Enriched sites are off-target sites found significant in at least one of the total libraries; hotspots are defined as enriched sites found significant in at least 2 out of 3 normalized libraries for each CRISPR complex. Significantly enriched translocation sites and hotspots in sequence read libraries were called using MACS2 (*q*-value cutoff -10^–10^), as previously detailed (12).

### Target site genotyping by amplicon deep sequencing

H27 reporter cells and HEK293T cells were exposed to dual nRGNs containing SpCas9^D10A^ or SpCas9^D10A^ variants as indicated under ‘Cell transfections’ and in Supplementary Tables S8 and S9. At 2 days post-transfection, genomic DNA extracted via the DNeasy Blood & Tissue Kit protocol (Qiagen; Cat. No.: 69506), was subjected to Illumina MiSeq next generation sequencing for obtaining 100 000 paired end reads from *EGFP* and *H2AX* target sequences in H27 and HEK293T cells, respectively. The NGS procedure was as follows. *EGFP*- and *H2AX*-specific PCR products (254 and 291 bp, respectively), were amplified with Phusion High-Fidelity Polymerase (Thermo Fisher Scientific; Cat. No.: #F-530L) and the PCR mixtures indicated in Supplementary Table S38. The primer pairs with adapter tag overhangs and the cycling parameters applied are specified in Supplementary Tables S39 and S40, respectively. After purification using AMPure XP beads (Beckman Coulter; Cat. No.: A63881), the resulting amplicons were subjected to PCR barcoding using Illumina tag-specific primer pairs with unique sequence combinations for demultiplexing and sample identification (Supplementary Table S41). The PCR mixtures and cycling parameters used for the preparation of barcoded amplicons are indicated in Supplementary Tables S42 and S40, respectively. After purification using AMPure XP beads, the concentrations of barcoded amplicons were determined by using the Qubit dsDNA HS assay kit (Invitrogen; Cat. No.: Q32854) and a Qubit2.0 fluorometer (Invitrogen). Sample quality control was done by capillarity electrophoresis through a 2100 Bioanalyzer system (Agilent). Finally, libraries of pooled barcoded amplicons were subjected to Illumina MiSeq deep sequencing with the reads corresponding to each individual sample being subsequently analysed with the aid of CRISPResso2 (34). In brief, after demultiplexing, adapter trimming of the paired end MiSeq raw reads (R1 and R2 fastq files) was performed with Cutadapt 2.10. Finally, the alignment of amplicon sequences to reference sequences was carried out by using CRISPResso2 set in the standard NHEJ mode. The codes applied in the CRISPResso2 analysis are available as Supplementary Information.

### Statistical analyses

With the exception of the genomic DNA samples used in the orthogonal HTGTS analyses, the researchers were not blinded to sample allocation. Data derived from a minimum of three biological replicates were analysed by GraphPad Prism 8.0.1 software package. Statistical significances were analyzed using the tests indicated in the figure legends. *P* values lower than 0.05 were considered to be statistically significant.

## RESULTS

### Comparing the performances of standard and high-specificity nucleases

We started by comparing the performance of wild-type SpCas9 with those of SpCas9 mutant variants SpCas9-KA (23), SpCas9-KARA (23), eSpCas9(1.1) (23), Sniper-Cas9 (24), SpCas9-HF1 (25), evoCas9 (26) and xCas9-3.7 (27) (Figure 1A). To this end, TURQ2 reporter cells were transfected with isogenic constructs expressing each of these nucleases (Figure 1A) mixed with plasmids synthesizing four different *mTurquoise2*-specific gRNAs. TURQ2 cells (28) contain a constitutively active *mTurquoise2* transgene (35) inserted at the human *AAVS1* ‘safe harbor’ locus (Figure 1B). Hence, *mTurquoise2* knockouts, resulting from small insertions and deletions (indels) generated after NHEJ-mediated DSB repair processes, report nuclease activity. To simultaneously confirm the higher specificity of SpCas9 variants over that of SpCas9, an *EGFP*-specific gRNA presenting three mismatches to an *mTurquoise2* sequence (gEGFP.3), was taken along (Figure 1C).

Flow cytometric quantification of mTurquoise2-negative cells showed that Sniper-Cas9 was the most consistent nuclease variant in that it yielded the most similar DNA cleaving activities when coupled to each of the four *mTurquoise2*-targeting gRNAs tested. However, once combined with gEGFP.3, Sniper-Cas9 led to off-target activities above background levels (Figure 1C and D). As expected, the native SpCas9 protein was the least specific enzyme of the panel (Figure 1C and D). The sub-set formed by the single, double and triple mutants SpCas9-KA, SpCas9-KARA and eSpCas9(1.1), respectively, yielded robust DNA cleaving activities except when combined with gTURQ.2 (Figure 1C). Moreover, eSpCas9(1.1) was also significantly less active than SpCas9 when coupled to gTURQ.3 (Figure 1C). Contrasting with gTURQ.1, that has a canonical 20-mer spacer fully complementary to the target DNA, the least performing gTURQ.2, similarly to gTURQ.3 and gTURQ.4, has a 21-mer spacer whose 5′ terminal guanine does not hybridize to the target sequence. Of notice, such non-canonical gRNAs are common gene-editing reagents due to a strong preference exhibited by frequently used RNA polymerase III promoters for guanines as first transcript nucleotide. Additional experiments performed in EGFP-expressing H27 reporter cells (29) showed that when compared with parental SpCas9, excluding Sniper-Cas9, all other high-specificity SpCas9 nucleases yielded substantially reduced gene knockout levels once coupled to gEGFP.21 whose 21-mer spacer is fully complementary to the target DNA (Supplementary Figure S1). Consistent with our results, gRNAs with 5′ non-hybridizing guanines and/or extended spacers were shown to significantly inhibit high-specificity SpCas9 nucleases, including eSpCas9(1.1), SpCas9-HF1 and evoCas9 but less so Sniper-Cas9 (24,26,36–38). Taken together, these data generally confirm the differential performance of the various SpCas9 variants vis-à-vis the wild-type SpCas9 protein in terms of their specificities and compatibilities with different gRNA moieties. Regarding the latter aspect, our data revealed that Sniper-Cas9 is the most compatible with a 5′ non-hybridizing guanine whilst evoCas9 the least. Furthermore, our results uncovered an inverse correlation between the increasing number of mutations in the nuclease set formed by SpCas9-KA, SpCas9-KARA and eSpCas9(1.1), and gene knockout frequencies when using gRNAs with 21-mer spacers (Figure 1C and Supplementary Figure S1).

### Functional screens identify a versatile set of high-specificity nickases

**Figure 2.**
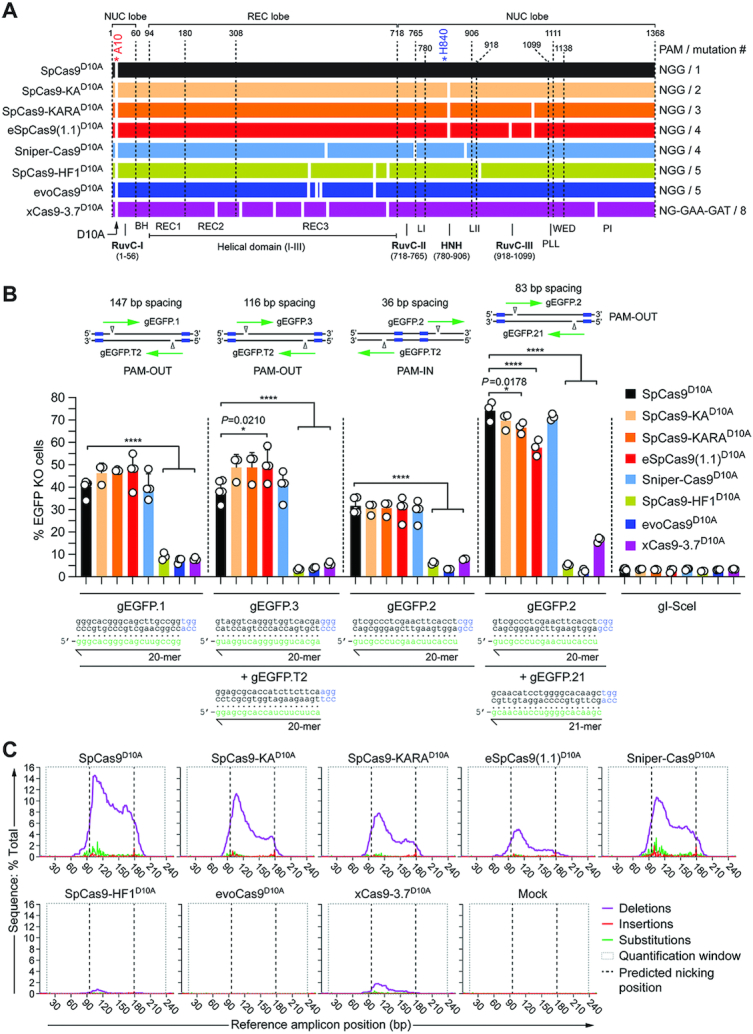
Comparing the activity of dual nRGNs based on SpCas9^D10A^ or SpCas9^D10A^ variants. (**A**) Schematics of original SpCas9^D10A^ and SpCas9^D10A^ variants generated for this study. Domains and mutations (white bars) in the nickases derived from the *S. pyogenes* type II CRISPR system are indicated. All nickases were obtained by introducing the RuvC-disabling D10A mutation into the nucleases depicted in Figure 1A. (**B**) Determining dual nRGN activities by gene knockout assays. EGFP-expressing H27 cells were transfected with constructs encoding the indicated dual nRGNs. Blue boxes, green arrows and open arrowheads in the insets indicate PAMs, gRNA spacers and nicking positions, respectively. Dual nRGNs with PAM-out and PAM-in arrangements were assessed. The non-targeting gRNA gI-SceI was used as a negative control. Gene knockout frequencies were determined by flow cytometry of EGFP-negative cells at 10 days post-transfection. Data are shown as mean ± S.D. of at least three independent biological replicates. Significance amongst the indicated datasets was calculated with one-way ANOVA followed by Dunnett's test for multiple comparisons; *0.01 < *P* < 0.05; ****P < 0.0001. (**C**) Characterization of dual nRGN ‘footprints by amplicon deep sequencing. H27 cells were exposed to dual nRGNs consisting of the indicated nickases loaded with gEGFP.2 and gEGFP.21. The types and frequencies of gene modifications detected at 48 h post-transfection within the *EGFP* target sequence are plotted.

After comparing SpCas9 nuclease performances, we generated isogenic constructs expressing the corresponding RuvC-disabled nicking forms; SpCas9-KA^D10A^, SpCas9-KARA^D10A^, eSpCas9(1.1)^D10A^, Sniper-Cas9^D10A^, SpCas9-HF1^D10A^, evoCas9^D10A^ and xCas9-3.7^D10A^ (Figure 2A). These enzymes were subsequently screened in quantitative assays as dual nRGNs for establishing their gene knockout activities upon simultaneous SSB formation. These assays were initiated by exposing H27 cells to dual nRGNs harboring the conventional SpCas9^D10A^ protein or each of the nicking variants coupled to different gRNA pairs (Figure 2B). The frequencies of gene knockouts resulting from the concerted action of nRGN pairs were measured through flow cytometry. Notably, these experiments showed that dual nRGNs containing SpCas9-KA^D10A^, SpCas9-KARA^D10A^, eSpCas9(1.1)^D10A^ or Sniper-Cas9^D10A^ can be as active as or more active than dual nRGNs built on the original SpCas9^D10A^ protein (Figure 2B). In contrast, dual nRGNs harboring SpCas9-HF1^D10A^, evoCas9^D10A^ or xCas9-3.7^D10A^ were less active than their respective SpCas9^D10A^-containing dual nRGN counterparts. Targeted deep sequencing analysis of ‘footprints’ induced by dual nRGNs containing the gRNA pair gEGFP.2/gEGFP.21 confirmed the flow cytometry data (Figure 2B) on their differential DNA cleavage activities (Figure 2C and Supplementary Figure S2). In most instances, this analysis further uncovered a clear preponderance of deletions over insertions and substitutions with a skewing of the deletions centred around the gEGFP.2 target site (Figure 2C and Supplementary Figure S2) which, of the two gRNAs, is the most effective when coupled to Cas9 nucleases (Supplementary Figures S1 and S3). Interestingly, sequence profiling of the most frequent ‘footprints’ revealed a paucity of insertions in cells treated with dual nRGNs harbouring members of the nickase variant sub-set formed by the single, double and triple mutants SpCas9-KA, SpCas9-KARA and eSpCas9(1.1), respectively (Figure 2C and Supplementary Figure S2B). This data suggests that the choice of nickase variant impacts the complexity of dual nRGN-induced target DNA changes.

The best-performing dual nRGNs, i.e., those with SpCas9-KA^D10A^, SpCas9-KARA^D10A^, eSpCas9(1.1)^D10A^ or Sniper-Cas9^D10A^, were less active when placed in a so-called PAM-in arrangement (Figure 2B). This data is in agreement with previous experiments using conventional dual nRGNs in which among PAM-out and PAM-in arrangements, the former normally yields higher DNA cleaving activities (39). Interestingly, not only for the original SpCas9^D10A^ nickase but also for each of the four best-performing SpCas9^D10A^ variants, the highest absolute frequencies of gene knockouts were detected in cultures exposed to the gRNA pair in which one of the members had a non-canonical 21-mer spacer (i.e. gEGFP.21) (Figure 2B). This result is especially notable for dual nRGNs containing eSpCas9(1.1)^D10A^ in that its parental eSpCas9(1.1) nuclease was poorly active when provided with gEGFP.21 but highly active when coupled to gEGFP.2 (Supplementary Figures S1 and S3). This data suggests that in the context of dual nRGNs a highly active complex can rescue or compensate for a poorly active neighbouring complex. In particular, it is possible that non-canonical 21-mer spacers mostly affect the RuvC domain of eSpCas9(1.1) which is functionally absent in dual nRGNs with eSpCas9(1.1)^D10A^. Finally, with the exception of xCas9-3.7 and xCas9-3.7^D10A^, western blot analysis revealed similar amounts of cleaving and nicking SpCas9 enzymes and dual nRGNs in transfected cells (Supplementary Figure S4). Importantly, dose-response experiments showed that gene knockout activities of RGNs and dual nRGNs containing xCas9-3.7 and xCas9-3.7^D10A^, respectively, were not affected or scarcely affected by increasing the amounts of these proteins (Supplementary Figure S5).

**Figure 3.**
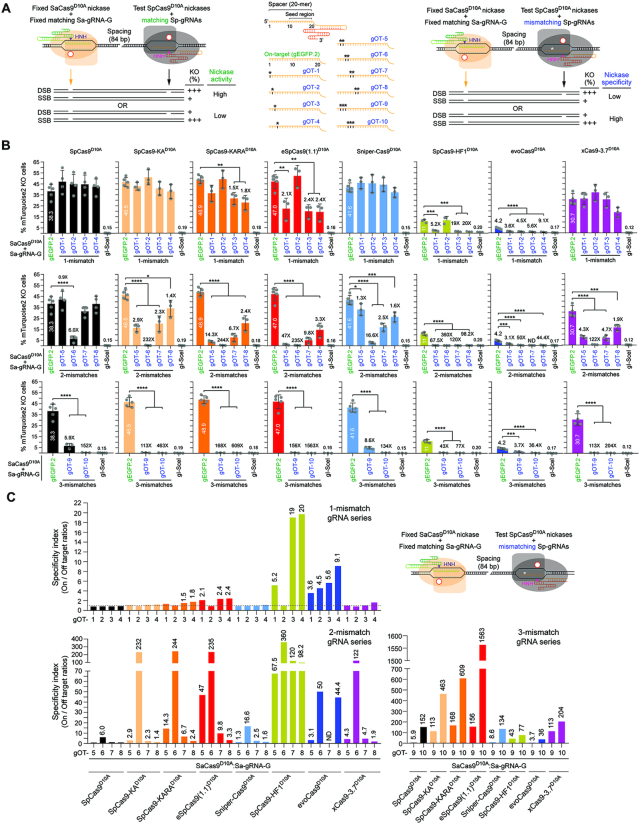
Comparing the performance of nRGNs based on SpCas9^D10A^ or SpCas9^D10A^ variants. (**A**) Cas9 orthogonal assay for determining the activity and specificity of nRGNs. A fixed *S. aureus* nRGN (orange) is introduced together with a test *S. pyogenes* nRGN (black) into reporter cells. Coordinated formation of SSBs at opposite strands of a bipartite reporter-encoding sequence by each nicking complex results in DSB-induced gene knockouts. Comparing the activities and specificities of different nickases can be assessed by loading *S. pyogenes* gRNAs with fully or partially hybridizing spacers (left and central panel, respectively). Test nRGN activities and specificities are directly and inversely proportional, respectively, to gene knockout frequencies (right panel). The fully matching spacer of *S. pyogenes* gEGFP.2 is drawn in relation to *S. pyogenes* gRNA spacers with 1-nt, 2-nt or 3-nt mismatches (asterisks) outside the seed region (central panel). (**B**) Comparing the specificity profiles of nRGNs with different nickases. Reporter cells were transfected with plasmids encoding the denoted nRGNs. The spacers of the three sets of off-target (OT) gRNAs, i.e., gOT-1 through gOT-4, gOT-5 through gOT-8 and gOT-9 plus gOT-10 have 1-nt, 2-nt and 3-nt mismatches, respectively, to the target sequence of gEGFP.2. Gene knockout levels were determined at 10 days post-transfection through flow cytometry of mTurquoise2-negative cells. Datasets correspond to mean ± S.D. of a minimum of three independent biological replicates. Significance between the indicated datasets was calculated with one-way ANOVA followed by Tukey's test for multiple comparisons; *0.01< *P* <0.05; **0.001< *P* <0.01; ***0.0001< *P* < 0.001; ****P< 0.0001. (**C**) The specificity indexes corresponding to DNA cleavage frequencies induced by nRGNs with *mTurquoise2*-matched gEGFP.2 divided by those triggered with *mTurquoise2*-mismatched gRNAs gOT-1 through gOT-10, are plotted. The statistically significant nRGN specificity indexes are presented above the respective bars.

Next, we sought to study the relationship between the activities and specificities of individual nRGNs endowed either with either SpCas9^D10A^ or each of the SpCas9^D10A^ variants. To detect targeted SSBs catalyzed by individual nRGNs, we established an assay based on delivering two types of SSB-inducing complexes into reporter cells. The first is a test *S. pyogenes* nRGN whose activity and specificity one wishes to determine; the second is a fixed *S. aureus* nRGN whose role is that of inducing a SSB off-set to that made by the test nRGN. Hence, this Cas9 orthogonal readout system permits sensitive and accurate measurements of nicking activities via recapitulating the *modus operandi* of dual nRGNs (Figure 3A, left panel). Crucially, by providing SpCas9^D10A^ variants with gRNAs presenting an array of mismatches to reporter sequences (Figure 3A, central panel), this readout system equally permits precisely assessing nRGN specificities which, as per definition, should inversely correlate with off-target nRGN activities (Figure 3A, right panel).

Previous experiments have indicated that RGN tolerance to DNA-gRNA mismatches roughly increases with the distance of these mismatches to the PAM (1,9). In keeping with these data, the 10–12 nts most proximal to the PAM have been proposed to constitute a ‘seed region’ in which DNA-gRNA mismatches are particularly detrimental for RGN activity (1,9). Hence, to increase the stringency of the nickase specificity screens in TURQ2 cells and maximize detecting differences in on-to-off target ratios (specificity indexes), we used a panel of gRNAs whose single, double and triple mismatches to reporter sequences were all located outside this ‘seed region’ (gOT-1 through gOT-10) (Figure 3A, central panel, Supplementary Figure S6). Furthermore, we chose to build the panel of mismatching gRNAs on basis of gEGFP.2 as its spacer is fully complementary to a *mTurquoise2* target site and led to comparably robust gene knockout frequencies irrespective of the SpCas9 nuclease used (Supplementary Figure S1). The *mTurquoise2*-specific *S. pyogenes* gEGFP.2 and its target site-mismatched derivatives were combined with a fixed fully-matching *S. aureus* gRNA (Sa-gRNA-G).

Consistent with the previous experiments using *S. pyogenes* gRNA pairs (Figure 2), gene knockout levels attained with gEGFP.2 and Sa-gRNA-G revealed that SpCas9-KA^D10A^, SpCas9-KARA^D10A^, eSpCas9(1.1)^D10A^ and Sniper-Cas9^D10A^ constitute robust SSB-inducing enzymes (Figure 3B, compare respective first bars). Equally in line with the previous data (Figure 2), SpCas9-HF1^D10A^ and evoCas9^D10A^ were the least performing nickases whilst, in this case, xCas9-3.7^D10A^ presented an intermediate nicking activity (Figure 3B, compare respective first bars). Together, these data demonstrate a striking difference in the tolerability of high-specificity SpCas9 nucleases to the D10A mutation and, hence, to their conversion into operative nickases.

The specificity assays involving loading the different SpCas9^D10A^ nickases with gRNAs partially complementary to the gEGFP.2 target DNA, generically showed a mismatch number-dependent decrease in gene knockout frequencies (Figure 3B and Supplementary Figure S7). Among the high-activity nickases, i.e. SpCas9-KA^D10A^, SpCas9-KARA^D10A^, Sniper-Cas9^D10A^ and eSpCas9(1.1)^D10A^, the latter was the most consistent in discriminating 1-nt, 2-nt and 3-nt gRNA–DNA mismatches, as indicated by the respective specificity indexes (Figure 3C). The high specificity of eSpCas9(1.1)^D10A^ was confirmed through gene knockout experiments using dual nRGNs exclusively with *S. pyogenes* gRNAs (Supplementary Figure S8). Amongst the low-activity nickases, i.e. SpCas9-HF1^D10A^ and evoCas9^D10A^, the former outperformed the latter in that, besides presenting higher on-target activity (Figure 3B), it was generally better at discriminating 1-nt, 2-nt and 3-nt mismatches (Figure 3C). Finally, the intermediate-activity xCas9-3.7^D10A^ nickase had its highest discriminating power at gRNA–DNA sequences with 2-nt and 3-nt mismatches (Figure 3C). Despite their low activities, SpCas9-HF1^D10A^, evoCas9^D10A^ and xCas9-3.7^D10A^ offer higher specificities than SpCas9^D10A^. In fact, for gRNA–DNA heteroduplexes with 3-nt mismatches, xCas9-3.7^D10A^ presented specificity indexes superior to those of Sniper-Cas9^D10A^, SpCas9-HF1^D10A^ and evoCas9^D10A^ (Figure 3C). Importantly, notwithstanding their varying on-target cleaving proficiencies, all engineered SpCas9^D10A^ variants were shown to be more specific than their parental SpCas9^D10A^ counterpart (Figure 3B and C).

We conclude that these reagents form a broad and versatile set of RNA-programmable nicking enzymes whose activities and/or specificities are superior to those of the commonly used SpCas9^D10A^ protein.

### Three-tier precision gene editing based on integrating high-specificity dual nicking RGN and truncated gRNA principles

**Figure 4.**
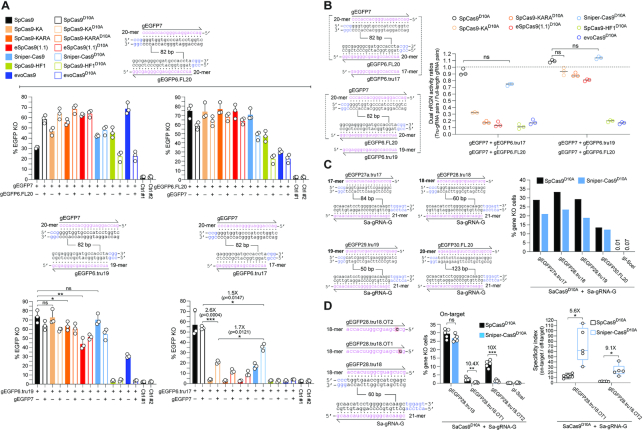
Investigating the integration of high-specificity dual nRGN and truncated gRNA principles. (**A**) Functional screening of high-specificity dual nRGNs with full-length and truncated gRNAs. EGFP-expressing H27 cells were exposed to dual nRGNs (open bars) containing a full-length gRNA pair (top panel) or expressing dual nRGNs harboring gRNA pairs with a truncated member (bottom panel). As references, H27 cells were exposed to RGNs (solid bars) with the same full-length gRNAs or truncated gRNAs. Results are presented as mean ± S.D. of independent biological replicates (*n* = 3). Significance between the indicated datasets was calculated using two-tailed Student's *t* tests. *0.01 < *P* < 0.05; **0.001< *P* < 0.01; *P*≥ 0.05 was considered non-significant (ns). (**B**) Testing the effect of full-length versus truncated gRNAs on dual nRGN activities. Dual RGN activity ratios obtained by dividing DNA cleavage frequencies induced with gRNA pairs containing a truncated member by those triggered with gRNA pairs with full-length gRNAs (panel A). Data are shown as mean ± S.D. of independent biological replicates (*n* = 3). Significance between the indicated datasets was calculated by one-way ANOVA followed by Dunnett's test for multiple comparisons; *P* ≥ 0.05 was considered non-significant (ns). (**C**) Assessing the activities of nRGNs with truncated gRNAs. The *S. aureus* SaCas9:Sa-gRNA-G complex was introduced into TURQ2 cells together with *S. pyogenes* complexes formed by SpCas9^D10A^ or Sniper-Cas9^D10A^ loaded with 17-, 18-, 19- or 20-mer gRNAs specific for *EGFP* and *mTurquoise2* sequences. The frequencies of SSBs induced by each of the *S. pyogenes* nRGNs were established by flow cytometry of mTurquoise2-negative cells. (**D**) Testing the specificities of nRGNs with truncated gRNAs. The *S. aureus* SaCas9:Sa-gRNA-G complex was delivered into TURQ2 cells together with *S. pyogenes* complexes formed by SpCas9^D10A^ or Sniper-Cas9^D10A^ coupled to 18-mer spacer gRNAs specific for *EGFP* and *mTurquoise2* sequences with no mismatches or with a single mismatch (red boxes) to a transgene sequence. PAMs for *S. pyogenes* and *S. aureus* Cas9 proteins are highlighted in blue (left panel). *S. pyogenes* nRGN activities were determined by mTurquoise2-negative cell quantification, with SpCas9^D10A^ showing significantly more tolerance to gRNA–DNA mismatches than Sniper-Cas9^D10A^ as presented in absolute and relative terms (graphs in middle and right panels, respectively). In the middle panel, the data are presented as mean ± S.D. of independent biological replicates (*n* = 5). Significance between the indicated datasets was calculated with two-tailed Student's *t* tests. **0.001 < *P* < 0.01; ***0.0001 < *P* < 0.001; *P* ≥ 0.05 was considered non-significant (ns). In the right panel, Box plot of independent biological replicates (*n* = 5), with significances calculated through two-tailed Student's *t* tests; *0.01 < *P* < 0.05. In all experimental settings, gene knockout levels, were determined by flow cytometry of mTurquoise2-negative cells at 10 days post-transfection.

Depending on their particular sequence, gRNAs with <20-mer spacers can significantly decrease SpCas9 off-target activities (40). It was postulated that amongst RGNs with 5′-truncated and full-length gRNAs, mismatches mostly destabilize the former leading to higher specificities (40). Hence, coupling high-specificity SpCas9 nucleases to validated truncated gRNAs is an appealing two-tier strategy to further reduce RGN off-target activities. Yet, similarly to 5′ non-hybridizing and extended gRNAs (36–38), truncated gRNAs can significantly hamper the on-target activities of high-specificity SpCas9 nucleases (23–25,31). To investigate a multi-tier approach for maximizing gene-editing tool precision based on integrating high-specificity dual nRGN and truncated gRNA principles, we tested the effect of truncated gRNAs on the activities of RGNs and dual nRGNs with high-specificity cleaving and nicking SpCas9 enzymes, respectively. To this end, H27 cells were subjected to dual nRGNs formed by gRNA pairs in which both members were full-length (i.e. gEGFP7/gEGFP6.FL20) (Figure 4A, open bars in top graphs) or one member was full-length and the other was truncated (i.e. gEGFP7/gEGFP6.tru19 and gEGFP7/gEGFP6.tru17) (Figure 4A, open bars in bottom graphs). As references, H27 reporter cells were exposed to RGNs with full-length gRNAs (i.e. gEGFP7 and gEGFP6.FL20) (Figure 4A, solid bars in top graphs) or truncated gRNAs (i.e. gEGFP6.tru19 and gEGFP6.tru17) (Figure 4A, solid bars in bottom graphs).

The cumulative gene knockout experiments revealed that the Sniper-Cas9 nuclease was the variant most compatible with truncated gRNAs with the 17-mer gRNA in particular only yielding gene knockouts once associated with this high-specificity nuclease (Figure 4A, solid cyan bar in bottom right-hand graph). These results are generically consistent with those of another study indicating that when compared to eSpCas9(1.1), SpCas9-HF1 and evoCas9, Sniper-Cas9 was least affected by 5′-end gRNA truncation (24). Crucially, nickases SpCas9-KA^D10A^, SpCas9-KARA^D10A^, eSpCas9(1.1)^D10A^ and Sniper-Cas9^D10A^, once combined with gRNA pair gEGFP7/gEGFP6.tru17, invariably performed better than their respective high-specificity nucleases provided with gEGFP6.tru17 (Figure 4A, bottom right-hand graph). In fact, although the nucleases SpCas9-KA, SpCas9-KARA and eSpCas9(1.1) presented robust activities with gEGFP6.tru19, their activities were reduced to background levels once coupled to gEGFP6.tru17 (Figure 4A, compare respective solid bars in bottom graphs). Moreover, amongst the high-specificity dual nRGNs, those harboring Sniper-Cas9^D10A^ achieved the highest absolute levels of target gene knockout (Figure 4A, open bars in bottom right-hand graph). This conclusion was further supported through complementary experiments in which gene knockout levels induced by dual nRGNs with truncated gRNAs were measured against those triggered by dual nRGNs containing full-length gRNA pairs (Figure 4B). Additional experiments involving a Cas9 orthogonal readout system and gRNAs with 17-, 18- and 19-mer spacers confirmed that dual nRGNs based on Sniper-Cas9^D10A^ are compatible with truncated gRNAs (Figure 4C). Follow-up experiments using the same Cas9 orthogonal assay, established that Sniper-Cas9^D10A^ endowed with truncated gRNAs can discriminate gRNA–DNA mismatches significantly better than SpCas9^D10A^ (Figure 4D). In fact, single base-pair mismatches located at PAM distal positions in 18-mer spacers sufficed to bring Sniper-Cas9^D10A^ nicking activities at near background levels (Figure 4D). Taken together, these data validate a three-tier precision gene editing strategy based on integrating into the dual nRGN concept, the high-specificity nickase and truncated gRNA principles.

### Standard and high-specificity dual nRGN activities are comparable at heterochromatic target sites

The previous functional screens of standard and high-specificity nucleolytic enzymes, demonstrated that eSpCas9(1.1)^D10A^ and Sniper-Cas9^D10A^ offer a favourable and complementary set of attributes, as judged by their efficiency, specificity and versatility. In particular, eSpCas9(1.1)^D10A^ and Sniper-Cas9^D10A^ display enhanced specificity and mostly retain the activity of SpCas9^D10A^. The specificity of eSpCas9(1.1)^D10A^ is superior to that of Sniper-Cas9^D10A^, yet Sniper-Cas9^D10A^ is more compatible with non-canonical gRNAs, including truncated gRNAs, than eSpCas9(1.1)^D10A^.

**Figure 5.**
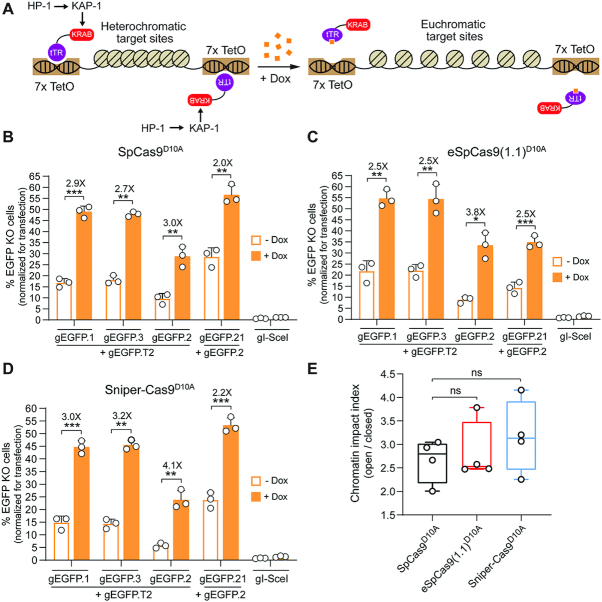
Comparing standard versus high-specificity dual nRGNs at alternate chromatin states. (**A**) Diagram of the experimental system. Doxycycline (Dox) availability regulates higher-order chromatin conformations that are controlled by KRAB-mediated recruitment of cellular silencing complexes to target sequences. In the absence of Dox, the tTR-KRAB fusion protein binds to *TetO* elements leading to the nucleation of cellular epigenetic modulators (e.g. KAP1 and HP1) and ensuing formation of compact heterochromatin at *EGFP* target sequences. In the presence of Dox, tTR-KRAB cannot bind to DNA, resulting in the maintenance of a relaxed euchromatin conformation at the same sequences. HEK.EGFP^TetO.KRAB^ cells treated or not treated with Dox were subjected to the indicated sets of gene-editing reagents that differed through their inclusion of either SpCas9^D10A^ (**B**), eSpCas9(1.1)^D10A^ (**C**) or Sniper-Cas9^D10A^ (**D**). After eliminating gene-editing reagents by sub-culturing and exposing both culture types to Dox, to assure transgene expression, *EGFP* knockout frequencies were determined by flow cytometry (Supplementary Figure S9A). Data are presented as mean ± S.D. of independent biological replicates (*n* = 3). Significance between datasets was calculated by two-tailed Student's *t* tests; *0.01 < *P* < 0.05; **0.001< *P* < 0.01; ***0.0001 < *P* < 0.001. (**E**) Cumulative chromatin impact indexes. Box plot presenting the chromatin impact indexes obtained by dividing gene knockout mean frequencies determined in the presence and absence of Dox (solid and open bars, respectively) (Figure S9b). Significance between the data points was calculated by one-way ANOVA followed by Dunnett's test for multiple comparisons; *P* ≥ 0.05 was considered non-significant (ns).

We thus progressed by investigating these nickases further, starting with their performance at alternate higher-order chromatin conformations. It is known that compact heterochromatic states can hinder gene-editing tool activities, including those of transcription activator-like effector nucleases, RGNs and standard dual nRGNs (30,31). To compare standard and high-specificity dual nRGNs at isogenic target sites packed in loose euchromatin versus compact heterochromatin, we employed HEK.EGFP^TetO.KRAB^ reporter cells (30). These cells allow for doxycycline-dependent control over Krüppel-associated box (KRAB)-mediated recruitment of endogenous epigenetic remodelling complexes to programmable nuclease target sites (Figure 5A and Supplementary Figure S9A). These complexes consist of, among other factors, KRAB-Associated Protein 1 (KAP1) and heterochromatin protein 1 (HP1) (Figure 5A). As expected, dual nRGNs based on SpCas9^D10A^, eSpCas9(1.1)^D10A^ and Sniper-Cas9^D10A^ were all significantly more active at euchromatic sequences in doxycycline-treated HEK.EGFP^TetO.KRAB^ cells than at the same heterochromatic sequences in untreated HEK.EGFP^TetO.KRAB^ cells (Figure 5B, C and D, respectively). Importantly, at KRAB-impinged heterochromatin, high-specificity dual nRGNs containing Sniper-Cas9^D10A^ or eSpCas9)1.1)^D10A^ performed similarly to standard dual nRGNs (Figure 5E and Supplementary Figure S9B).

### High-specificity dual nRGNs outperform standard dual nRGNs at genomic sequences

**Figure 6.**
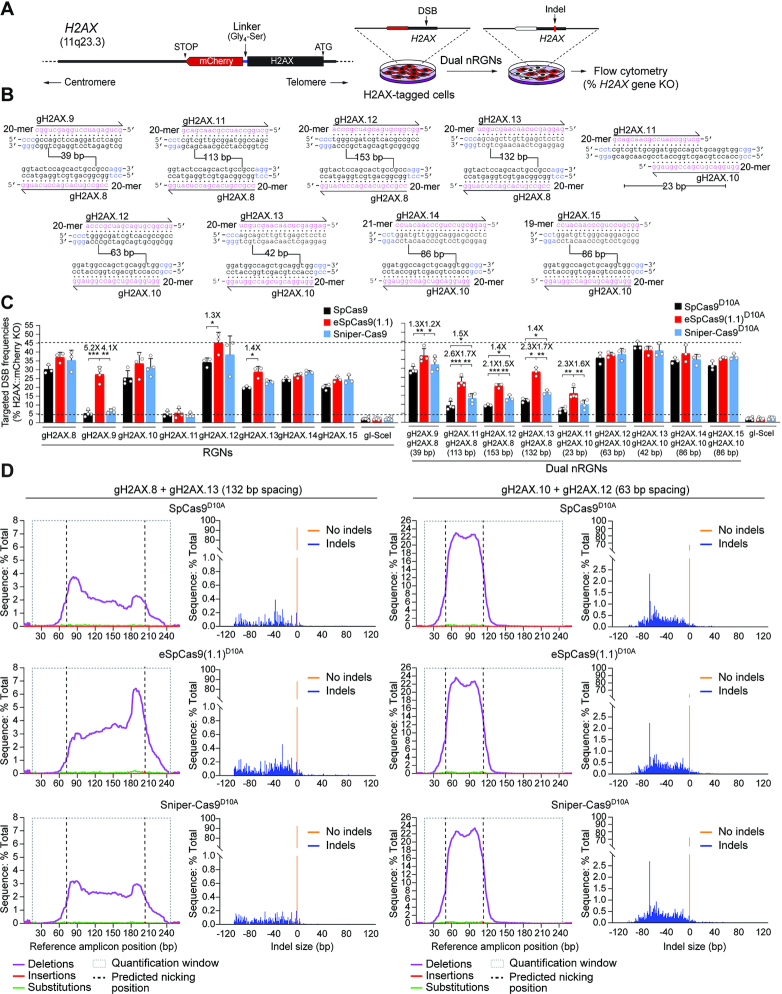
Testing the activity of RGNs and dual nRGNs at human genomic DNA. (**A**) Schematics of readout system. HeLa cells containing the *H2AX* gene in-frame with a *mCherry* reporter are exposed to dual nRGN components. Target DNA cleavage is assessed through flow cytometric quantification of mCherry-negative cells resulting from DSB-induced indels at *H2AX* sequences. (**B**) *H2AX*-targeting gRNAs. The gRNA spacer nucleotides are drawn annealing to the respective target DNA strands. PAM nucleotides are highlighted in blue. Numbers within broken line correspond to the spacing between gRNA pair members using as reference the base pair positions at which nicking occurs. (**C**) Functional screening of RGNs and dual nRGNs with standard or variant SpCas9 proteins at *H2AX*. H2AX::mCherry^+^ HeLa cells were transfected with plasmids expressing the indicated combinations of RGN and dual nRGN elements. DNA cleaving activities were assessed through flow cytometry of mCherry-negative cells at 10 days post-transfection. Dashed lines, corresponding to the lowest and highest DNA cleaving frequencies measured. Data are presented as mean ± S.D. of at least three independent biological replicates. Significance between the indicated datasets was calculated by two-tailed Student's *t* tests; *0.01 < *P* < 0.05; **0.001 < *P* < 0.01; ***0.0001 < *P* < 0.001. (**D**) Characterization of dual nRGN ‘footprints’ at *H2AX* alleles. The types and frequencies of gene modifications within the indicated dual nRGN target sequences were determined at 48 h post-transfection by amplicon deep sequencing of HEK293T cells.

To compare the activities and specificities of dual nRGNs based on standard versus high-specificity nickases at endogenous genomic DNA, we targeted *H2AX* alleles in-frame with a *mCherry* reporter in HeLa cells. This set-up allows for sensitive flow cytometric quantification of DNA cleaving activities (Figure 6A). In initial experiments, SpCas9, eSpCas9(1.1), Sniper-Cas9, and their respective nicking derivatives, were used together with a panel of eighteen gRNAs (Figure 6B). In line with earlier results (Figure 2, Supplementary Figures S1 and S3) (22), it was observed that low to intermediate RGN cleaving activities conferred by certain gRNAs can be bypassed via combining these gRNAs with a nickase and a second gRNA addressed to an off-set sequence; thus, effectively forming an operational dual nRGN complex (Figure 6C, compare left and right graphs). Most importantly, amidst the nine randomly selected PAM-out gRNA pairs covering a wide range of spacing lengths (Figure 6B), five yielded significantly higher *H2AX* knockout frequencies when combined with eSpCas9(1.1)^D10A^ instead of SpCas9^D10A^ (Figure 6C, right graph). Albeit to a lesser extent than eSpCas9(1.1)^D10A^, three out of the nine gRNA pairs performed also better with Sniper-Cas9^D10A^ than with SpCas9^D10A^ (Figure 6C, right graph). Moreover, four gRNA pairs led to similar *H2AX* knockout frequencies, independently of the nickase to which they were joined (Figure 6C, right graph). These data indicate that dual nRGNs based on eSpCas9(1.1)^D10A^ can outperform SpCas9^D10A^-containing dual nRGNs in inducing target DNA cleavage.

Targeted deep sequencing analysis of HEK293T cells exposed to dual nRGNs containing gRNA pairs gH2AX.8/gH2AX.13 and gH2AX.10/gH2AX.12, was consistent with the relative gene knockout levels measured by flow cytometry of HeLa reporter cells treated with the same gene-editing reagents (Figure 6D and Supplementary Figure S10A). This analysis further uncovered a vast representation of deletions over insertions and substitutions. In fact, sequence profiling revealed neither insertions nor substitutions amongst the ten most frequent ‘footprints’ (Supplementary Figure S10B and S10C). Interestingly, deletions triggered by dual nRGNs with the most spaced gRNAs (i.e. gH2AX.8/gH2AX.13) were often centred around either one of the target sites (Supplementary Figure S10B); whereas deletions induced by dual nRGNs with the least spaced gRNAs (i.e. gH2AX.10/gH2AX.12) mostly encompassed the intervening sequence (Supplementary Figure S10C). This data suggests that gRNA spacing impacts the complexity of dual nRGN-induced target DNA changes.

**Figure 7.**
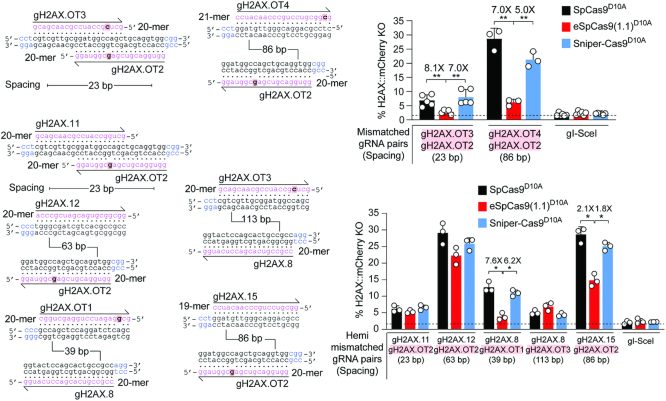
Testing the specificity of dual nRGNs at human genomic DNA. Specificity assay comparing standard and variant dual nRGNs containing gRNAs with mismatches to *H2AX* in both spacers (mismatched gRNA pairs) or only in one of the two spacers (hemi-mismatched gRNA pairs). *H2AX* and gRNA spacer sequences are drawn hybridizing to each other with mismatched and PAM nucleotides highlighted in red boxes and blue lettering, respectively. In these assays, the DNA mismatch discriminating power (specificity) of individual dual nRGNs inversely correlates with *H2AX* gene knockout frequencies. H2AX::mCherry^+^ HeLa cells were transfected with constructs expressing the denoted dual nRGNs. *H2AX* gene knockout frequencies were determined by flow cytometry of mCherry-negative cells at 10 days post-transfection. The results are expressed as mean ± S.D. of a minimum of three independent biological replicates. Significance between the indicated datasets was calculated by two-tailed Student's *t* tests; *0.01 < *P* < 0.05; **0.001 < *P* < 0.01.

**Figure 8.**
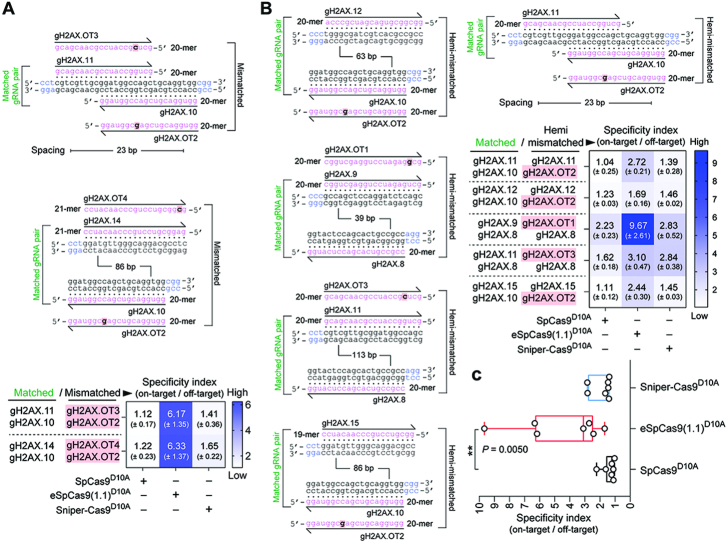
Testing the effect of sequence mismatches on standard and high-specificity dual nRGN activities. (**A**) Comparing standard versus variant dual nRGNs with DNA-mismatched gRNA pairs. Dual nRGNs based on SpCas9^D10A^, eSpCas9(1.1)^D10A^ or Sniper-Cas9^D10A^, coupled to *H2AX*-matched or mismatched gRNA pairs, were introduced into H2AX::mCherry^+^ HeLa cells. The heatmap presents dual nRGN specificity indexes (mean ± S.D.) resulting from dividing the gene knockout frequencies induced with *H2AX*-matched gRNA pairs by those attained with the respective mismatched gRNA pairs. (**B**) Comparing standard versus variant dual nRGNs with DNA hemi-mismatched gRNA pairs. Dual nRGNs based on SpCas9^D10A^, eSpCas9(1.1)^D10A^ or Sniper-Cas9^D10A^, linked to *H2AX*-matched or hemi-mismatched gRNA pairs, were delivered into H2AX::mCherry^+^ HeLa cells. The heatmap depicts dual nRGN specificity indexes (mean ± S.D.) derived from dividing the gene knockout frequencies achieved with *H2AX*-matched gRNA pairs by those attained with the respective hemi-mismatched gRNA pairs. (**C**) Cumulative specificity indexes. Box plot of the specificity indexes presented in the heatmaps of panels A and B. In all experimental settings, gene knockout levels, corresponding to at least three independent biological replicates, were determined by flow cytometry of mCherry-negative cells at 10 days post-transfection. Significance between datasets was calculated with one-way ANOVA followed by Dunnett's test for multiple comparisons; ** 0.001< *P* < 0.01.

To strictly challenge the specificity of dual nRGNs based on SpCas9^D10A^, eSpCas9(1.1.)^D10A^ and Sniper-Cas9^D10A^, we next designed gRNAs bearing single nt mismatches to *H2AX* sequences mapping at PAM distal positions. HeLa cells expressing mCherry-tagged H2AX were exposed to dual nRGNs formed by gRNAs in which both or only one of their spacers contained 1-nt mismatches to *H2AX* sequences (Figure 7, top and bottom panels, respectively). In agreement with previous results (Figure 3B and C, Supplementary Figure S8B and C), these DNA cleaving specificity assays revealed that, amongst dual nRGNs based on SpCas9^D10A^, Sniper-Cas9^D10A^ and eSpCas9(1.1.)^D10A^, the latter are the most robust in discriminating subtle gRNA–DNA mismatches (Figure 7). This conclusion was strengthened through complementary experiments in which gene knockout levels triggered by dual nRGNs with DNA mismatching gRNAs were measured against those induced by dual nRGNs containing the respective, fully matching, gRNAs (Figure 8). We conclude that dual nRGNs based on eSpCas9(1.1)^D10A^ are valuable gene-editing tools in that they can outperform standard dual nRGNs at both the activity and specificity levels.

### High-specificity dual nRGN ‘tiptoeing’ achieves selective cleavage of genomic sites with high similarity to off-target sequences

**Figure 9.**
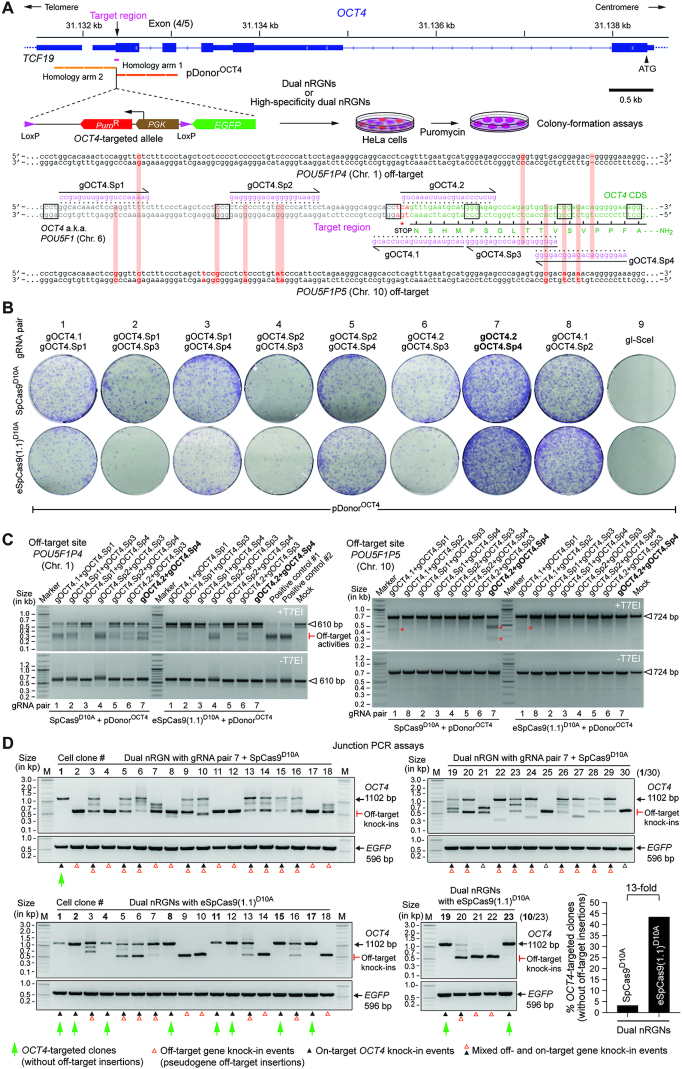
Homology-directed gene targeting of genomic sites sharing high sequence identity with off-target sequences using conventional or high-specificity complexes. (**A**) *OCT4* gene targeting set-up. The *OCT4* target region is presented in relation to similar sequences in *OCT4* pseudogenes *POU5F1P4* and *POU5F1P5* located at chromosomes 1 and 10, respectively. HeLa cells were transfected with pDonor^OCT4^ and plasmids encoding dual nRGNs containing SpCas9 or high-specificity dual nRGNs harboring eSpCas9(1.1)^D10A^. Donor construct pDonor^OCT4^ is designed to knock-in into *OCT4* the EGFP coding sequence together with a floxed marker gene that confers resistance to puromycin in colony-formation assays. PAM and gRNA sequences are boxed and magenta colored, respectively. DNA-gRNA mismatches are highlighted by vertical red bars. (**B**) Colony-formation assays on HeLa cells. HeLa cells genetically modified through the delivery of the indicated gene-editing tools are scored after puromycin selection and Giemsa staining. (**C**) Detection of dual nRGN off-target activities. T7EI-based genotyping assays were performed on DNA from puromycin-resistant HeLa cell populations initially exposed to pDonor^OCT4^ and the indicated dual nRGN elements. T7EI-specific products diagnostic for mutant *POU5F1P4* and *POU5F1P5* loci generated by the installation of indels after NHEJ-mediated DSB repair, are labelled as ‘Off-target activities’ and asterisks, respectively. Products representing intact loci are instead marked by open arrowheads. (**D**) Characterization of HDR-mediated *OCT4* gene editing specificity achieved by dual nRGNs containing SpCas9^D10A^ or eSpCas9(1.1)^D10A^. Junction PCR analysis on genomic DNA from puromycin-resistant HeLa cell clones from cultures treated with pDonor^OCT4^, SpCas9^D10A^, gOCT4.2 and gOCT4.Sp4 (*n* = 30) or with pDonor^OCT4^, eSpCas9(1.1)^D10A^, gOCT4.2 and gOCT4.Sp4 (*n* = 23). For details see Supplementary Figure S12. Lanes M, GeneRuler DNA Ladder Mix molecular weight marker.

*OCT4* (a.k.a. *POU5F1*) is a coveted gene editing target owing to its essentiality for the maintenance of embryonic stem cells (ESCs) as well as for the maintenance and generation of iPSCs through cellular reprogramming (41,42). *OCT4* is equally crucial during early human embryogenesis (43). The selective modification of *OCT4* though programmable nucleases is, however, challenging due to the presence of *OCT4* pseudogenes in different chromosomes. Moreover, off-target sites located in *OCT4* pseudogenes combined with the particularly high sensitivity of pluripotent stem cells (PSCs) to few DSBs (44–46), renders the isolation of *OCT4*-edited PSCs highly inefficient (14,47,48). Indeed, *OCT4* tagging experiments in PSCs involving recombination between target and pDonor^OCT4^ sequences (Figure 9A) triggered with TALENs (47) or RGNs (48) retrieved, respectively, no iPSC (*n* = 48) or only eight ESC (*n* = 288) clones that were correctly edited. Thus, to compare the capacity of standard and high-specificity dual nRGNs to distinguish target DNA from highly similar off-target genomic sequences, we performed HDR-mediated gene knock-in experiments at *OCT4* using pDonor^OCT4^ (Figure 9A). In particular, we asked whether the heightened single base-pair resolution of high-specificity dual nRGNs permits discriminating highly similar genomic sequences from each other by ‘tiptoeing’ over preexisting indels or single nucleotide polymorphisms (SNPs). To this end, HeLa cells were first transfected with pDonor^OCT4^ mixed with constructs encoding a panel of dual nRGNs based on SpCas9^D10A^ or eSpCas9(1.1)^D10A^ (Figure 9A and B). Colony-formation assays revealed that the number of cells acquiring puromycin resistance varied as a function of the nickase and gRNA pair used (Figure 9B). Most importantly, off-target analysis of genomic DNA from puromycin-resistance HeLa cell populations revealed that dual nRGNs with eSpCas9(1.1)^D10A^ were substantially more specific than their SpCas9^D10A^-containing counterparts (Figure 9C). Indeed, six out of seven gRNA pairs readily led to DSB formation at *POU5F1P4* when coupled to SpCas9^D10A^, whilst only two of these gRNA pairs induced DSBs at this locus once linked to eSpCas9(1.1)^D10A^ (Figure 9C, left panel). At *POU5F1P5*, out of eight gRNA pairs tested, two and one yielded off-target cleavage when coupled to SpCas9^D10A^ and eSpCas9(1.1)^D10A^, respectively (Figure 9C, right panel). The fact that *POU5F1P4* and *POU5F1P5* overlap with coding genes (i.e. *ASH1L* and *HERC4*, respectively) further compounds the genotype of cells suffering off-target DSBs at these loci (Supplementary Figure S11). Moreover, clonal analysis assessing gene knock-ins at *OCT4* and pseudogene loci, established that the specificity of HDR-mediated gene editing was substantially higher (13-fold) when dual nRGNs were endowed with eSpCas9(1.1)^D10A^ instead of SpCas9^D10A^ (Figure 9D and Supplementary Figure S12). In particular, from 30 randomly selected HeLa cell clones derived from cultures exposed to pDonor^OCT4^ and SpCas9^D10A^-based dual nRGNs, only 1 was properly edited, i.e., was targeted at *OCT4* (Figure 9D, top panels green arrow) and lacked mistargeted insertions at *OCT4* pseudogenes (Supplementary Figure S12). In contrast, 10 out of 23 clones isolated from cultures treated with pDonor^OCT4^ and eSpCas9(1.1)^D10A^-based dual nRGNs, were properly edited (Figure 9D, bottom panels green arrows). Thus, although dual nRGNs are prevalently used for NHEJ-mediated gene knockouts, their capacity to induce HDR-mediated gene knock-ins broadens their applicability, especially if built on high-specificity nickases. Indeed, this data indicates that NHEJ- and HDR-based gene editing with dual nRGNs harboring eSpCas9(1.1)^D10A^ permits a more judicious access to specific genomic variants through ‘tiptoeing’ over short preexisting polymorphisms.

**Figure 10.**
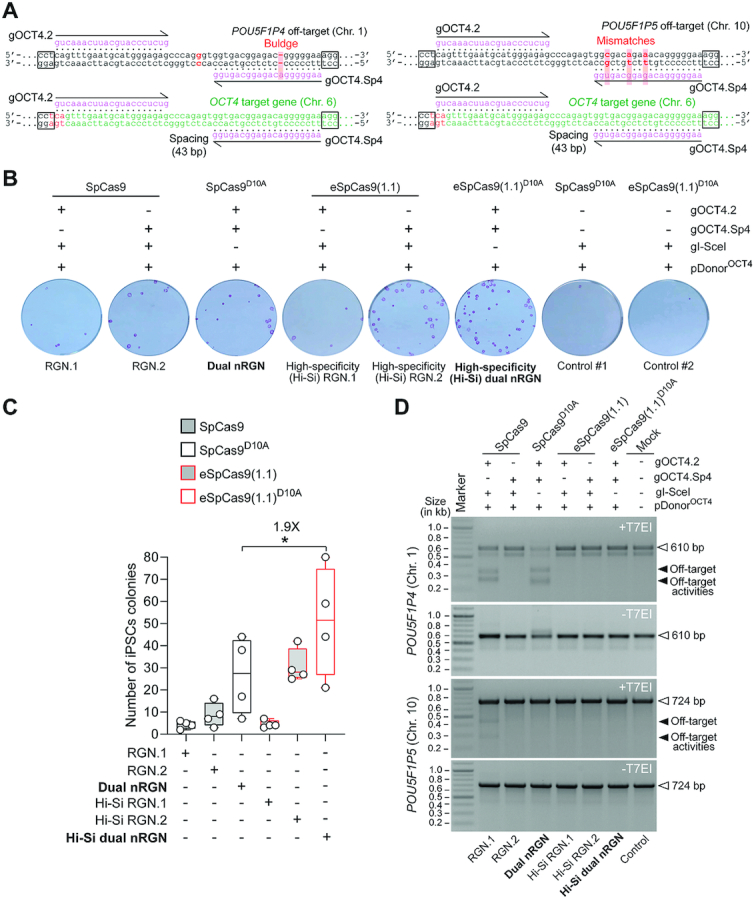
Homology-directed gene targeting in iPSCs at *OCT4* sequences highly similar to off-target sites using conventional or high-specificity complexes. (**A**) RGN and dual nRGN target sites and pseudogene off-target sequences. The *OCT4* sequence (green) is depicted next to similar sequences in *OCT4* pseudogenes *POU5F1P4* and *POU5F1P5* (black) located at chromosomes 1 and 10, respectively. PAM and gRNA sequences are boxed and magenta colored, respectively. DNA-gRNA mismatches and a gRNA buldge are highlighted by vertical red bars. (**B**) Colony-formation assays on iPSCs. iPSCs genetically modified through the transfer of the indicated gene-editing reagents are identified after puromycin selection and staining for the pluripotency marker alkaline phosphatase. (**C**) Quantification of genetically modified iPSCs. The numbers of alkaline phosphatase-positive iPSC colonies resulting from four independent biological replicates are presented in box plots with minimum and maximum. Significance between the indicated datasets was calculated by two-tailed Student's *t* tests; *0.01 < *P* < 0.05 (**D**) Detection of RGN and dual nRGN off-target activities. T7EI-based genotyping assays were carried out on DNA from puromycin-resistant iPSC populations initially subjected to pDonor^OCT4^ and the indicated RGN and dual nRGN components. T7EI-specific species diagnostic for mutant *POU5F1P4* and *POU5F1P5* loci generated by the induction of indels after NHEJ-mediated DSB repair, are marked by solid arrowheads. Products corresponding to intact loci are instead marked by open arrowheads. Marker, GeneRuler DNA Ladder Mix molecular weight marker.

We proceeded by performing gene knock-in experiments targeting active *OCT4* alleles in iPSCs using pDonor^OCT4^ and gRNA pair members gOCT4.2 and gOCT4.Sp4. The latter gRNA forms a bulge at *POU5F1P4* and displays three mismatches to *POU5F1P5* (Figure 10A). The coupling of this gRNA pair to SpCas9^D10A^ or eSpCas9(1.1)^D10A^ yielded high and similar levels of genetically modified HeLa cells (Figure 9B). In the *OCT4* gene targeting experiments in iPSCs, next to dual nRGNs, we extended the testing to RGNs with SpCas9 or eSpCas9(1.1). The highest numbers of puromycin-resistant iPSCs labeled with the pluripotency marker alkaline phosphatase (AP) were observed in cultures initially exposed to dual nRGNs harboring eSpCas9(1.1)^D10A^ (Hi-Si dual nRGN; Figure 10B and C). Importantly, off-target DSBs in puromycin-resistance iPSCs subjected to these high-specificity dual nRGNs were detected neither at *POU5F1P4* nor *POU5F1P5* (Hi-Si dual nRGN; Figure 10D). In contrast, robust off-target DSB activities at *POU5F1P4* were detected in puromycin-resistant iPSCs subjected to dual nRGNs containing SpCas9^D10A^ (Dual nRGN; Figure 10D). In HeLa cells, off-target cleavage provoked by these conventional dual nRGNs was readily detected at *POU5F1P5* as well (Figure 9C), possibly reflecting the higher initial transfection efficiencies achieved in these cells.

As expected, RGN complex SpCas9:gOCT4.2 (RGN.1), by presenting complementarity to pseudogene sequences, cleaved *POU5F1P4* and *POU5F1P5* (Figure 10D). Notably, despite having the same gRNA as SpCas9:gOCT4.2, off-target cleavage was not detected with eSpCas9(1.1):gOCT4.2 (Hi-Si RGN.1). This result is consistent with the fact that gOCT4.2 has an extended spacer and a 5′ non-hybridizing guanine, features previously implicated in eSpCas9(1.1) hindrance here (Figure 1C) and elsewhere (24,36–38). Moreover, the highest numbers of AP^+^ iPSC colonies obtained by using high-specificity dual nRGNs further support our earlier finding that hindrance of eSpCas9(1.1)-mediated DSB formation by non-canonical gRNAs (Figure 2, Supplementary Figures S1 and S3) can be overcome, now in a gene knock-in setting, by converting this nuclease into a nickase and placing it in a dual nRGN context (Figure 10B and C).

Taken together, our results suggest that incorporating eSpCas9(1.1)^D10A^ in dual nRGNs offers the possibility for enhancing the frequencies and specificities of gene knockouts and gene knock-ins, while retaining the broad genomic coverage of dual nRGN designs resulting from their compatibility with wide spacing between nRGNs as well as non-canonical gRNAs. Concerning the latter aspect, as aforesaid, it is possible that non-canonical gRNAs mostly affect the RuvC domain of eSpCas9(1.1) which is rendered dispensable in dual nRGNs with eSpCas9(1.1)^D10A^ (Figure 2, Supplementary Figures S1 and S3).

**Figure 11.**
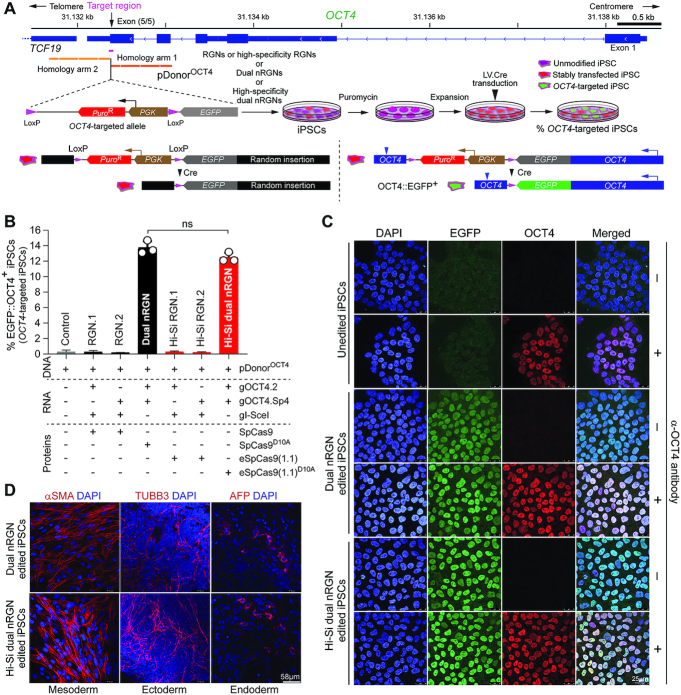
Quantification and characterization of *OCT4* targeted iPSCs by standard versus high-specificity RGNs and dual nRGNs. (**A**) Experimental set-up and genetic assay for detecting *OCT4* gene targeting events. iPSCs were transfected with pDonor^OCT4^ and constructs expressing RGNs containing SpCas9 or eSpCas9(1.1) or high-specificity dual nRGNs harbouring SpCas9^D10A^ or eSpCas9(1.1)^D10A^. pDonor^OCT4^ knock-ins into *OCT4* the *EGFP* coding sequence and a floxed marker gene conferring puromycin resistance. Functional genetic assays, The Cre-mediated selectable marker removal and OCT4::EGFP fusion product assembly reports precisely targeted iPSCs. Stable OCT4::EGFP expression arises from *OCT4* transcription initiation and termination regulatory elements. (**B**) Quantification of OCT4 targeted iPSCs. The frequencies of OCT4::EGFP^+^ iPSCs in puromycin resistant populations were determined by EGFP-directed flow cytometry. Data are shown as mean ± S.D. of three independent biological replicates. Significance between the indicated datasets was calculated through two-tailed Student's *t* test with *P* ≥ 0.05 considered non-significant (ns). (**C**) Confocal fluorescence microscopy analysis of *OCT4* edited iPSCs. OCT4::EGFP^+^ iPSCs edited by dual nRGNs with SpCas9^D10A^ or by high-specificity dual nRGNs with eSpCas9(1.1)^D10A^, were subjected to indirect and direct fluorescence microscopies for detecting OCT4 and EGFP, respectively. Nuclei were identified by DAPI staining. Parental, unedited, iPSCs served as negative controls. Unedited and edited iPSC populations that were not incubated with the OCT4-specific primary antibody provided for staining controls. (**D**) Testing the multilineage differentiation potential of *OCT4* edited iPSCs. OCT4::EGFP^+^ iPSCs edited by dual nRGNs with SpCas9^D10A^ or by high-specificity dual nRGNs with eSpCas9(1.1)^D10A^ were induced to differentiate into cell lineages corresponding to the three embryonic germ layers, i.e. mesoderm, ectoderm and endoderm. Markers for each of these germ layers are indicated. Nuclei were stained with DAPI.

To compare the frequencies of properly targeted *OCT4* alleles in iPSCs genetically modified through RGNs or dual nRGNs with standard or high-specificity enzymes, we exploited the genetic readout system built in pDonor^OCT4^. In this system, Cre-mediated assembly of a traceable OCT4::EGFP fusion product reports targeted iPSCs in puromycin-resistance populations (Figure 11A). Notably, EGFP-directed flow cytometry detected *OCT4*-targeted iPSCs at levels substantially above background exclusively in cell populations genetically modified by standard and high-specificity dual nRGNs (Figure 11B). Finally, EGFP and OCT4 confocal microscopy analyses confirmed accurate tagging of the endogenous OCT4 protein in these iPSC populations (Figure 11C), which were subsequently capable of differentiating into cells representing the three embryonic germ layers (Figure 11D).

Taken together, these data demonstrate that gene-editing involving homologous recombination between pDonor^OCT4^ and *OCT4* was best achieved by using high-specificity dual nRGNs based on eSpCas9(1.1)^D10A^. In fact, these dual nRGNs outperformed conventional and high-specificity RGNs as well as conventional dual nRGNs in terms of avoiding off-target cleavage at highly similar pseudogene sequences (Figures 9C and 10D) and, at the same time, yielding precise gene knock-ins (Figures 9D and 11B).

### Unbiased genome-wide assessment of specificity profiles of cleaving versus nicking RGNs

**Figure 12.**
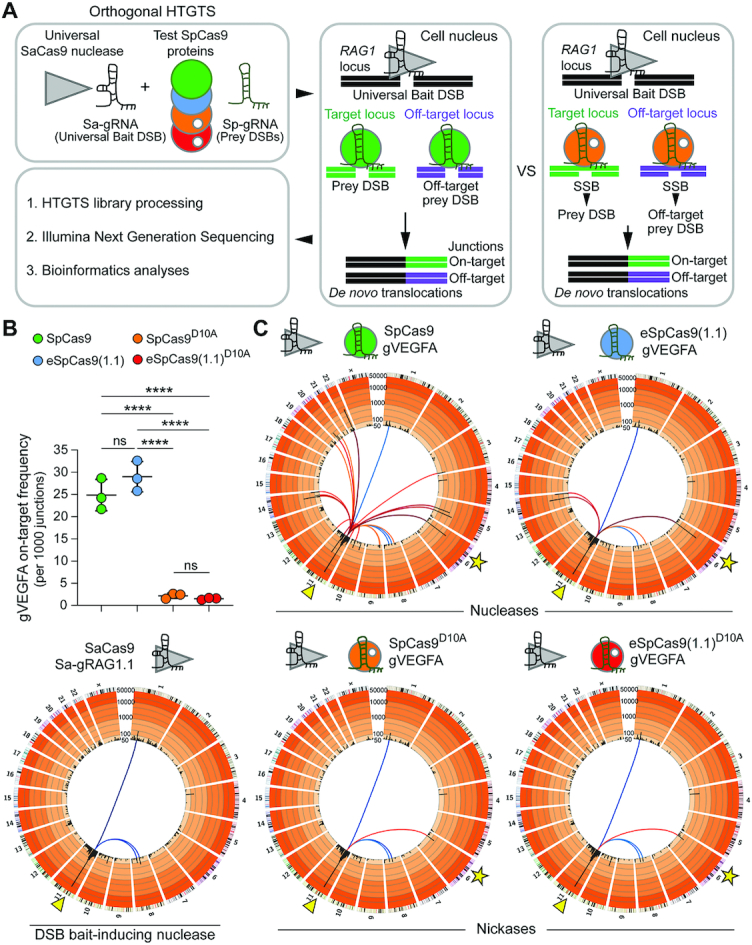
Investigating the specificity of cleaving and nicking RGNs by unbiased genome-wide orthogonal HTGTS analyses. (**A**) Schematics of the orthogonal HTGTS pipeline for genome-wide assessments of off-target effects induced by RGNs versus nRGNs. A universal *S. aureus* cleaving RGN complex (SaCas9:Sa-gRAG1.1) is used to generate bait DSBs at *RAG1*; cleaving and nicking test RGN complexes induce DSBs and SSBs, respectively, at target and off-target loci. Prey DSBs catalyzed by *S. pyogenes* nucleases and prey DSBs generated from SSBs catalyzed by *S. pyogenes* nickases, are measured through deep sequencing of translocation junctions involving bait and prey chromosomal termini. (**B**) On-target DSB frequencies. Number of translocations to the *VEGFA* target locus per 1000 junctions induced by nucleases SpCas9:gVEGFA and eSpCas9(1.1):gVEGFA or by nickases SpCas9^D10A^:gVEGFA and eSpCas9(1.1)^D10A^:gVEGFA. HEK293T cells were transfected with constructs expressing the indicated RGNs and nRGNs (*n* = 3 biological replicates). At 2 days post-transfection, orthogonal HTGTS analyses were carried out on genomic DNA previously screened by target-site genotyping assays (Supplementary Figure S13B). ****P< 0.0001 one-way ANOVA and Tukey's multiple pairwise-comparisons. (**C**) Cumulative orthogonal HTGTS analyses from three biological replicates. Each library was normalized to 11932 junctions. Arrowheads in Circos plots mark the location of the bait DSB on chromosome 11 induced by the universal *S. aureus* RGN for all sequence read libraries; stars in Circos plots mark the *VEGFA* target site of test *S. pyogenes* RGNs and test *S. pyogenes* nRGNs on chromosome 6. Blue-graded lines connected to the *RAG1* locus indicate bait nuclease-related off-targets; red-graded lines linked to the *RAG1* locus indicate on-target (star) and off-targets resulting from RGNs and nRGNs containing the promiscuous gRNA gVEGFA. Black bars correspond to 5 Mb bins across each chromosome with enrichment levels presented on a custom color-coded log scale by order of magnitude. Hotspots are established when significantly enriched translocation sites are present in at least two out of three replicates (MACS2; *q*-value cutoff –10^–10^).

**Figure 13.**
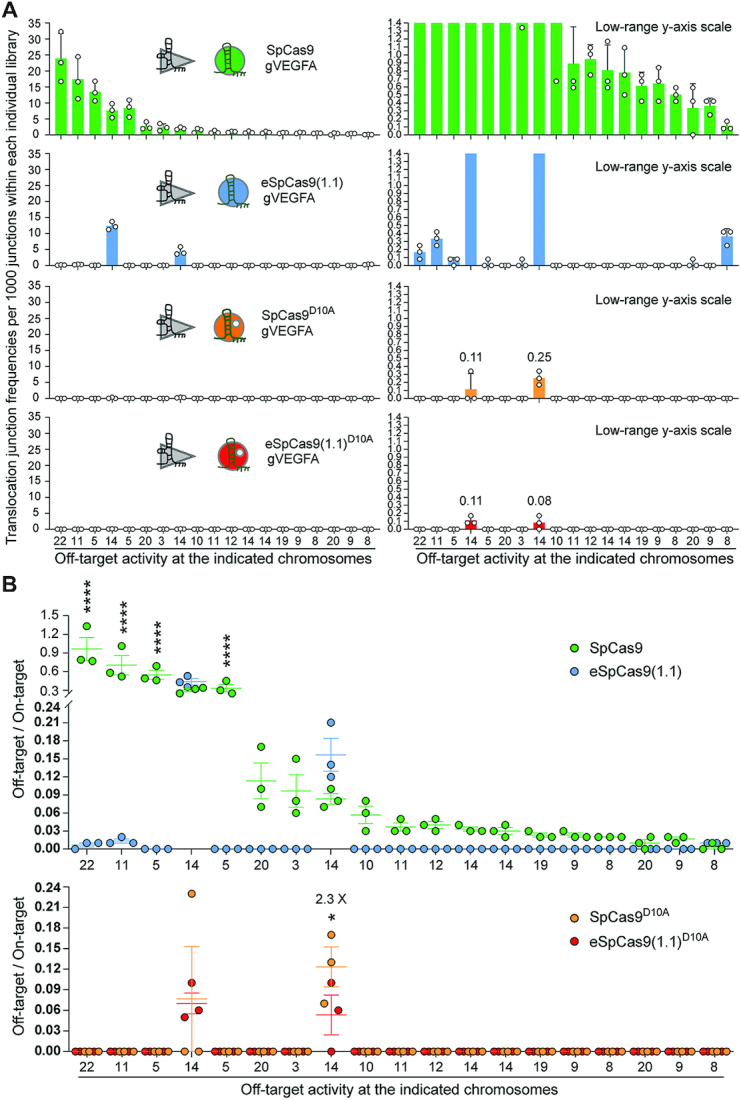
Ranking the off-target sites of RGNs and nRGNs containing a promiscuous gRNA. (**A**) Distribution and frequencies of gVEGFA off-target sites across the human genome. Translocation junction frequencies associated with each of the detected off-target sites plotted with a broad and narrow Y-axis value ranges (left and right panels, respectively). Off-targets were ranked according to their frequencies in sequence read libraries corresponding to SpCas9:gVEGFA complexes. The chromosomes in which each of the off-target sites map are shown. Chromosome coordinates of detected off-target sites and frequencies of translocation junctions per 1000 junctions within each individual library are specified in Supplementary Figure S14. (**B**) Activity indices at the various off-target hotspots. Hotspots are defined as translocation enriched sites found significant in at least 2 out of 3 normalized libraries for each CRISPR complex (MACS2; *q*-value cutoff –10^–10^). Ratios between the number of translocations to an off-target site and the number of translocations to the on-target site at *VEGFA* in libraries normalized to 11932 junctions; asterisks mark statistically significant differences in off-target activity indices in normalized libraries (MACS2; *q*-value cutoff -10^-10^). *P = 0.0217; ****P< 0.0001 two-way ANOVA and Holm–Sidak's multiple comparison tests. Error bars correspond to mean and SEM from three independent biological replicates.

Although most SSBs are resolved through conservative DNA repair processes (20,21), they can nonetheless progress to DSBs in instances in which an advancing replication fork hits them and collapses (49). Therefore, unbiased and sensitive methods for detecting genomic changes resulting from SSBs or nicks are warranted for guiding the refinement of precise gene-editing tools and strategies based on nRGNs. Recently, to measure and examine off-target effects induced by nRGNs, we have adapted the high-throughput genome-wide translocation sequencing (HTGTS) assay by incorporating SaCas9 nuclease and a universal *RAG1*-targeting gRNA (Sa-gRAG1.1) for inducing bait DSBs (Figure 12A) (14). As this assay, dubbed orthogonal HTGTS, permits comparing RGN and nRGN off-target profiles as well, herein we investigated side-by-side the genome-wide specificities of SpCas9, eSpCas9(1.1), SpCas9^D10A^ and eSpCas9(1.1)^D10A^. Thus, after validating that SpCas9 variants are compatible with the *VEGFA*-targeting gRNA gVEGFA (Supplementary Figure S13A), previously used in genome-wide DSB detection assays (23), we introduced this gRNA and universal SaCas9:Sa-gRAG1.1 complexes together with each of the test nucleases or test nickases into HEK293T cells (*n* = 3). As expected, indels at *RAG1* and *VEGFA* were readily detected in cells exposed to SaCas9:Sa-gRAG1.1 and gVEGFA-bound nucleases (Supplementary Figure S13B). In contrast, indels were only detected at *RAG1* in cells subjected to SaCas9:Sa-gRAG1.1 and gVEGFA-bound nickases, confirming that nRGNs have a low mutagenic potential (Supplementary Figure S13B). The higher on-target effects induced by nucleases over nickases was independently confirmed by orthogonal HTGTS analysis (Figure 12B and C, Supplementary Figures S14 and S15). Most importantly, this analysis further demonstrated a gradual overall decrease in off-target effects in cells treated with SpCas9, eSpCas9(1.1), SpCas9^D10A^ and eSpCas9(1.1)^D10A^ (Figure 13A and B). As expected, SpCas9 was more disruptive to the genome than eSpCas9(1.1) (Figures 12C and 13B, Supplementary Figures S14 and S15). Interestingly, a subtle differential off-target site preference for SpCas9 and eSpCas9(1.1) was uncovered within an enriched translocation region at chromosome 11 (Supplementary Figures S14 and S16). In the case of nicking SpCas9^D10A^ and eSpCas9(1.1)^D10A^ enzymes, off-target activities were detected at two chromosome 14 regions, with the latter enzyme presenting a 2.3-fold lower off-target activity index at one of these two genomic regions (Figure 13B, lower panel). Taken together, the orthogonal HTGTS data indicate that, amongst the four proteins tested, eSpCas9(1.1)^D10A^ is the least genome-disrupting thus constituting a preferable tool for precise genome editing based on targeted DSB or SSB formation.

## DISCUSSION

We report that the enhanced specificity of a representative panel of SpCas9 mutants is transportable to their respective SpCas9^D10A^ variants. Indeed, albeit differing significantly in their sequence-specific and strand-specific nuclease activities, the assembled RNA-guided nickases exhibit specificities that are markedly superior to that of the commonly used SpCas9^D10A^ protein. By using an array of functional screens, we have identified high-specificity nickases that can, when operating as dual nRGNs, outperform their conventional dual nRGN counterparts in terms of target DNA cleaving activities and specificities. Concerning the latter aspect, after selecting Sniper-Cas9^D10A^, we provide a proof-of-concept for a three-tier precision gene editing strategy based on integrating into the dual nRGN concept (18,19), the truncated gRNA (40) and high-specificity nickase principles. Moreover, high-specificity dual nRGNs containing eSpCas9(1.1)^D10A^ were found to be more versatile than high-specificity RGNs harboring eSpCas9(1.1). In particular, besides retaining the broad genomic space coverage characteristic of dual nRGN designs, dual nRGNs based on eSpCas9(1.1)^D10A^ were compatible with gRNAs containing extended spacers or 5′ non-hybridizing guanines. These data indicate that these non-canonical gRNAs mostly hinder the RuvC domain of eSpCas9(1.1), which is functionally absent in dual nRGNs with eSpCas9(1.1)^D10A^. Importantly, orthogonal HTGTS analyses detected scant off-target activity at the genome-wide level in cells exposed to eSpCas9(1.1)^D10A^ and the promiscuous gRNA gVEGFA (23). Finally, targeted deep sequencing analysis suggests that the choice of nickase variant and gRNA spacing have an impact on the type and uniformity of ‘footprints’ installed by dual nRGNs.

A broad range of small and large chromosomal edits can be established following NHEJ or HDR of targeted DSBs. These edits include *de novo* translocations for studying cancer (50), genomic deletions and gene knockouts for investigating *cis*-acting and *trans*-acting elements, and gene knock-ins to modify, repair or tag endogenous genes (1,5,51,52). However, targeting specific loci or allelic variants in diploid cells is challenging, especially when these elements share high sequence identity with regions located elsewhere in the genome. Yet, for the most part, eukaryotic genomes consist of such recurrent multiple-copy regions that include retroelements, amplified gene clusters, gene paralogs and pseudogenes (53). Moreover, knowledge about genetic differences amongst genomes or amongst different alleles or loci in an individual genome, e.g. SNPs and indels, is crucial for complementing correlative genome-wide association studies (GWAS) with causal genotype-phenotype relationships (54,55). Another aspect concerns the fact that, as genome editing expands its reach into therapeutic gene editing, the human genetic variation is likely to start receiving further attention. Indeed, it has been shown that SNPs and indels can alter the activity and specificity of RGNs in a genotype-dependent manner, including at loci underpinning human disorders (56). Therefore, there is a pressing need to develop genome editing technologies permitting a judicious access to specific chromosomal sequences while averting related off-target sites. To this end, we exploited genomic indels or SNPs and the heightened single base-pair discriminating power of dual nRGNs with eSpCas9(1.1)^D10A^ to selectively target *OCT4* and avoid off-target *OCT4* pseudogene sequences. In contrast, conventional dual nRGNs readily led to disrupted *OCT4* pseudogene loci. The ‘tiptoeing’ of dual nRGNs over SNPs permitted retrieving iPSCs expressing EGFP-tagged OCT4. Despite the superior sensitivity of dual nRGNs containing eSpCas9(1.1)^D10A^ to single-base pair mismatches, a limitation of the ‘tiptoeing’ approach is the need to design and test various gRNA pairs per target region as off-target activities were still detected when using eSpCas9(1.1)^D10A^ and certain gRNA pairs.

In conclusion, after screening and identifying improved RNA-guided nickases, we demonstrate their utility for expanding precise genomic engineering involving the engagement of the NHEJ and HDR pathways. Recent developments in genome editing entail using nicking Cas9 proteins as such or fused to heterologous DNA-modifying moieties. These genome editing approaches include; (i) HDR-mediated chromosomal insertion of donor DNA spanning from single base-pairs to entire transgenes through nicking of target and donor templates, i.e. *in trans* paired nicking (14,28,57,58), and (ii) donor DNA-free installation of single base-pair transversions through base editing (59–61) and any base-pair substitution or short indel through prime editing (62). The herein investigated high-specificity nickases and gene editing strategies involving the recruitment of either NHEJ or HDR pathways might enrich and complement these emerging technologies directed at seamless and scarless genomic engineering.

## DATA AVAILABILITY

All data generated and analysed in this study are included in the article and supplementary files. Additional raw datasets that support the findings of this work are available upon request. The deep sequencing libraries corresponding to the orthogonal HTGTS analyses are deposited in the Gene Expression Omnibus (GEO) repository and are available via accession code GSE153471. The amplicon deep sequencing reads corresponding to dual nicking RGN target-site genotyping analyses are deposited at the NCBI Sequence Read Archive (SRA) database under the BioProject accession PRJNA675830.

## Supplementary Material

gkaa1236_Supplemental_Files
