# Supplementary material for: Precise and broad scope genome editing based on high-specificity Cas9 nickases
Source: Nucleic Acids Res. 2021 Jan 4;49(2):1173–98. doi: 10.1093/nar/gkaa1236 (PMC7826261; doi:10.1093/nar/gkaa1236)
Supplement: gkaa1236_Supplemental_Files [file gkaa1236_supplemental_files.zip › Supplementary Figures S1-S16 (Wang et al.).pdf]

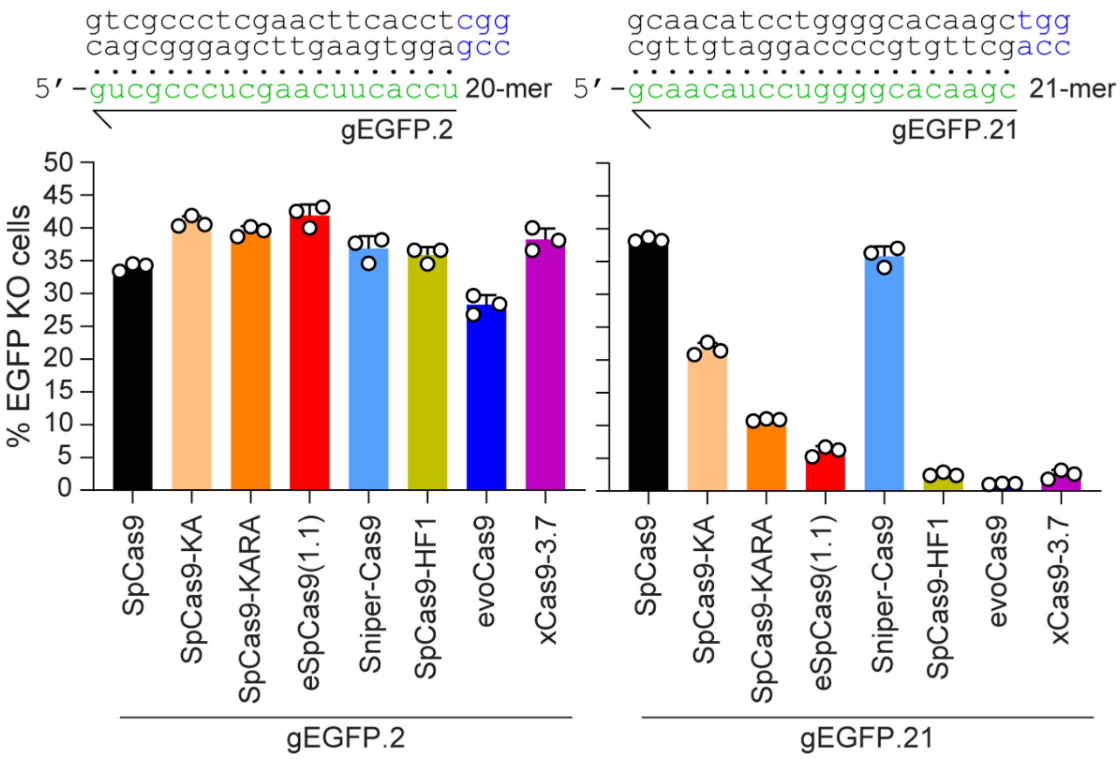

Supplementary Figure S1. Determining RGN activities. H27 cells were transfected with constructs expressing the indicated proteins and gRNAs. The gRNA gEGFP.2 targets a canonical protospacer; whereas gEGFP.21 targets an extended protospacer. Spacer and PAM nucleotides are highlighted in green and blue, respectively. Gene knockout frequencies were assessed at 10 days post-transfection through flow cytometry of EGFP-negative cells. Data are presented as mean ± S.D. of three independent biological replicates.

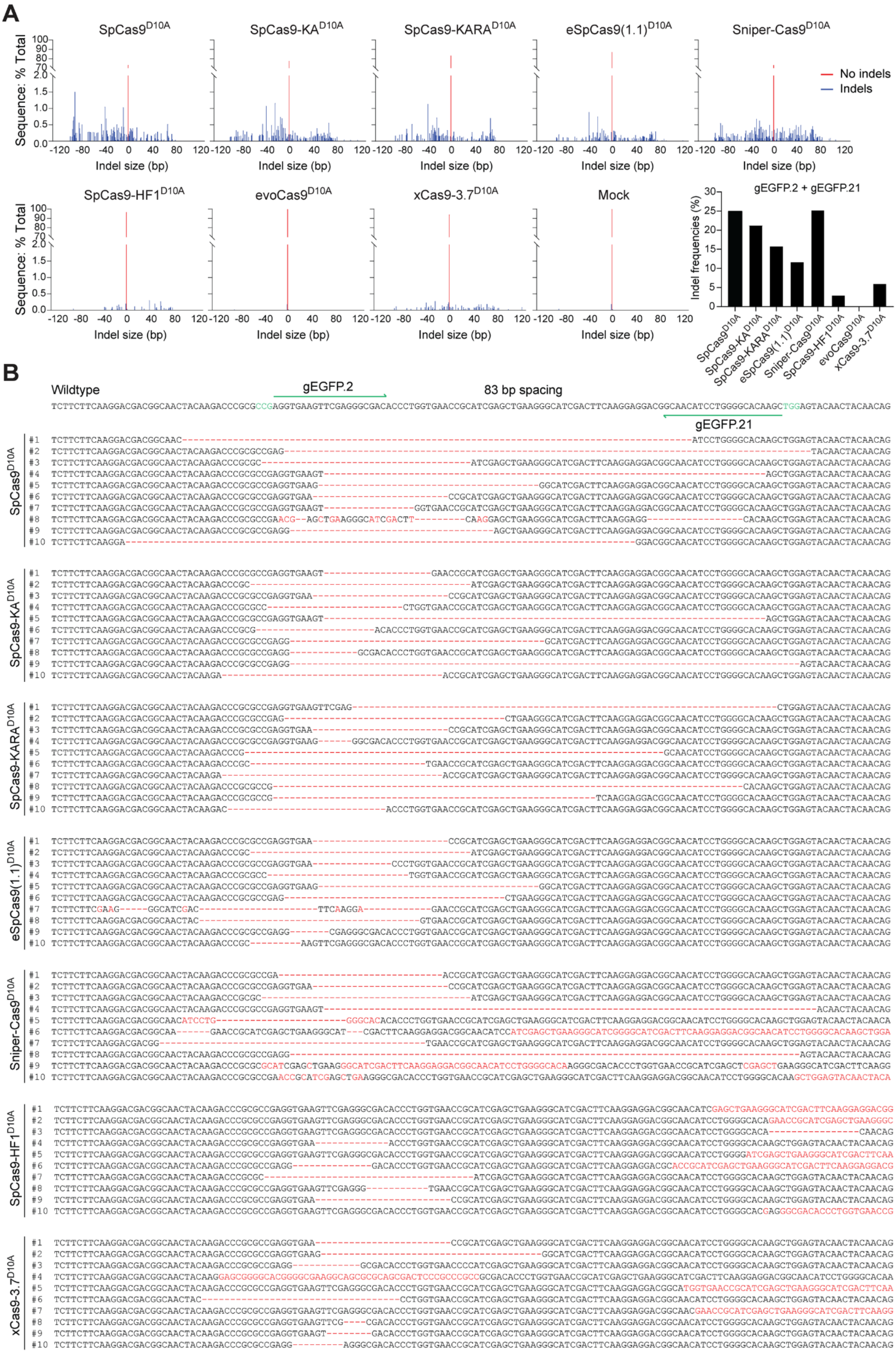

Supplementary Figure S2. Characterization of dual nRGN "footprints" at *EGFP* by deep sequencing analysis. (**A**) Distribution of indel sizes and cumulative indel frequencies and (**B**) top 10 most frequent target-site "footprints" in H27 cells treated with the indicated dual nRGNs.

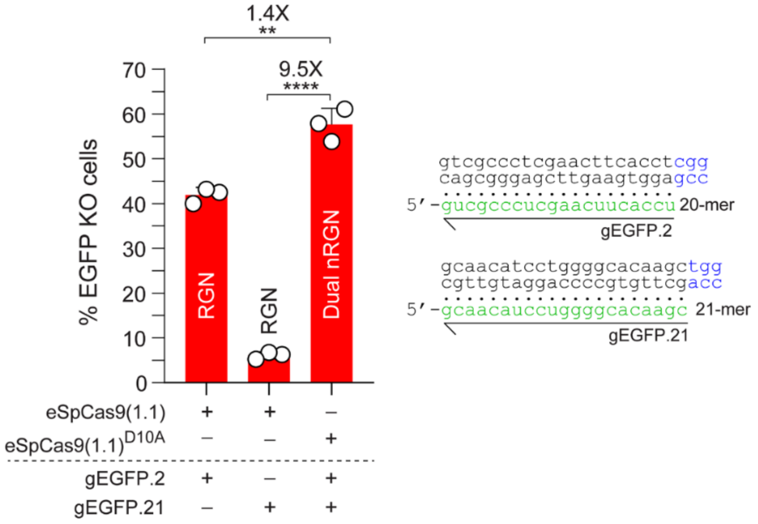

Supplementary Figure S3. Comparing gEGFP.2 and gEGFP.21 in the context of RGNs and respective dual nRGN. The gRNA gEGFP.2 hybridizes to a canonical protospacer; whereas gEGFP.21 hybridizes to an extended protospacer. Spacer and PAM nucleotides are highlighted in green and blue, respectively. The graph gathers the datapoints corresponding to H27 cells transfected with plasmids expressing eSpCas9(1.1) and gEGFP.2 or eSpCas9(1.1) and gEGFP.21 (**Supplementary Figure S1**); or expressing both gRNAs and eSpCas9(1.1)^{D10A} (**Figure 2**). Gene knockout frequencies were measured at 10 days post-transfection through flow cytometry of EGFP-negative cells. Data are presented as mean ± S.D. of three independent biological replicates. Significance values were calculated by two-tailed Student's *t* tests; **0.001< *P* < 0.01; ****P* < 0.0001.

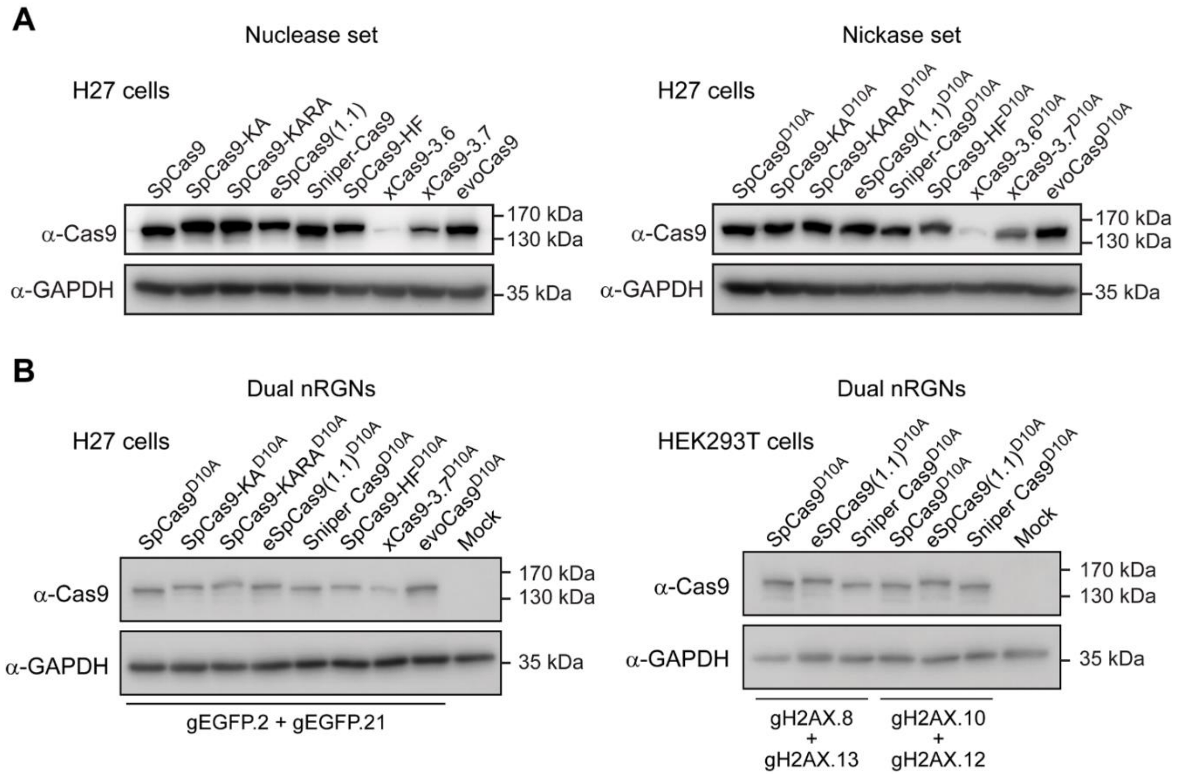

Supplementary Figure S4. Protein expression analysis. (**A**) H27 cells were transfected with plasmids driving the synthesis of the indicated nucleases and nickases. Datasets obtained using xCas9-3.6 and xCas9-3.6^{D10A} were not included in this work due to the low expression levels of these proteins. (**B**) H27 and HEK293T cells were transfected with plasmids driving the synthesis of dual nRGNs for targeted DNA cleavage at *EGFP* and endogenous *H2AX* alleles, respectively. The proteins present in cell lysates at 48 h post-transfection were detected by western blotting using a SpCas9-specific antibody. The immunodetection of the housekeeping GAPDH protein provided for internal loading controls.

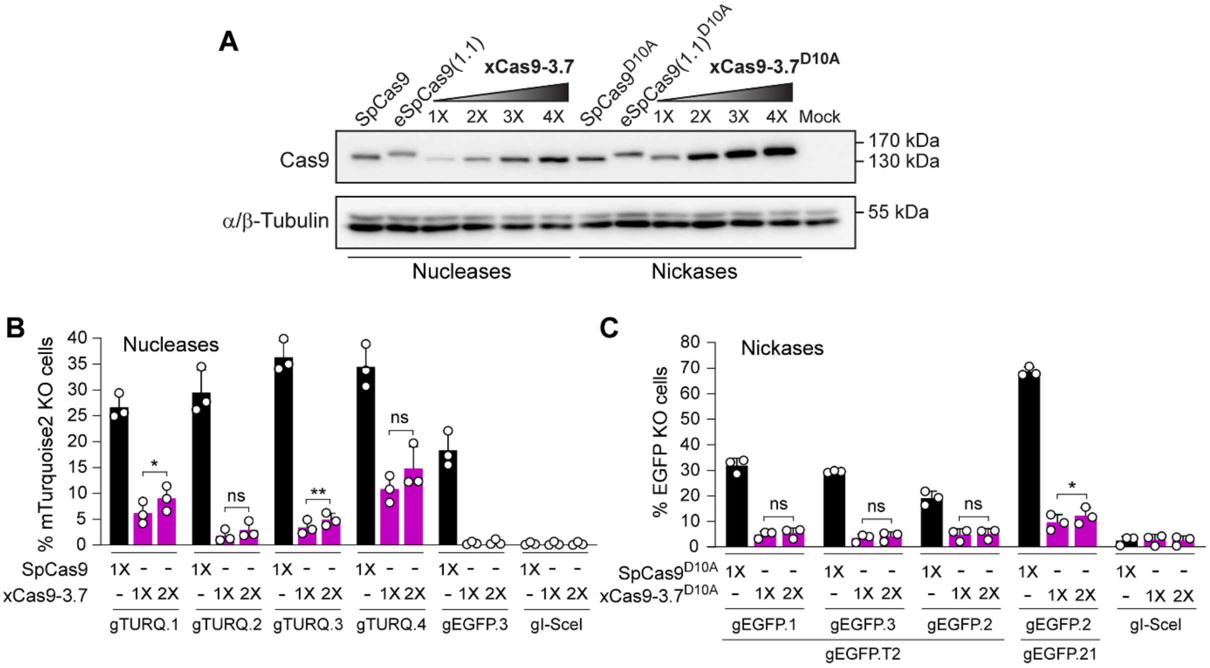

Supplementary Figure S5. xCas9-3.7 and xCas9-3.7^{D10A} dose-response experiments. (**A**) Western blot analysis on different amounts of xCas9-3.7 and xCas9-3.7^{D10A}. H27 cells were transfected with plasmids expressing the indicated nucleases and nickases. Four different doses of the plasmids encoding xCas9-3.7 and xCas9-3.7^{D10A} were applied. The proteins present in cell lysates at 48 h post-transfection were detected by western blotting using a SpCas9-specific antibody, with lysates from mock-transfected H27 cells yielding the negative control. The immunodetection of α/β-Tubulin served as internal loading controls. (**B** and **C**) Dose-response gene knockout experiments with xCas9-3.7 and xCas9-3.7^{D10A}. TURQ2 and H27 cells were transfected with constructs expressing the indicated RGN and dual nRGN elements (left and right panels, respectively). Two doses of the plasmids encoding xCas9-3.7 and xCas9-3.7^{D10A} were applied, with cells exposed to the non-targeting gRNA gI-SceI yielding negative controls. Gene knockout levels were determinefned at 10 days post-transfection through flow cytometry of reporter-negative cells. Datasets correspond to mean ± S.D. of three independent biological replicates. Significance between the indicated datasets was calculated through two-tailed Student's *t* tests. *0.01< P < 0.05; **0.001< P < 0.01; $P \geq 0.05$ was considered non-significant (ns).

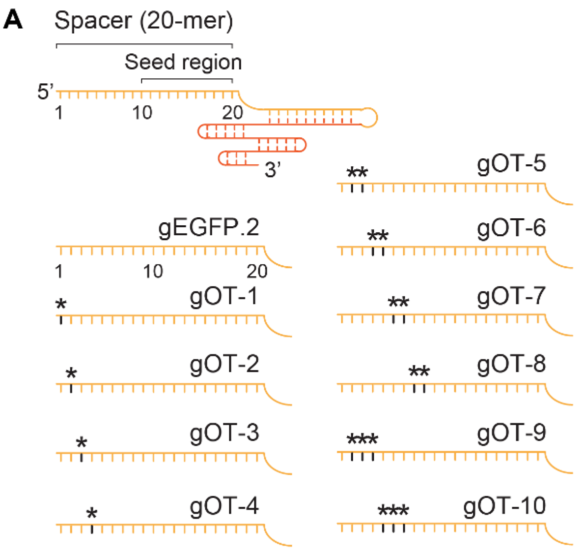

B

		1	2	3	4	5	6	7	8	9	10	11	12	13	14	15	16	17	18	19	20	PAM		
Target	T	G	T	C	G	C	C	C	T	C	G	A	A	C	T	T	C	A	C	C	T	C	G	G
gOT-1	G	A	U	C	G	C	C	C	U	C	G	A	A	C	U	U	C	A	C	C	U	C	G	G
gOT-2		G	C	C	G	C	C	C	U	C	G	A	A	C	U	U	C	A	C	C	U	C	G	G
gOT-3		G	U	U	G	C	C	C	U	C	G	A	A	C	U	U	C	A	C	C	U	C	G	G
gOT-4		G	U	C	A	C	C	C	U	C	G	A	A	C	U	U	C	A	C	C	U	C	G	G
gOT-5		G	C	U	G	C	C	C	U	C	G	A	A	C	U	U	C	A	C	C	U	C	G	G
gOT-6		G	U	C	A	U	C	C	U	C	G	A	A	C	U	U	C	A	C	C	U	C	G	G
gOT-7		G	U	C	G	C	U	U	U	C	G	A	A	C	U	U	C	A	C	C	U	C	G	G
gOT-8		G	U	C	G	C	C	C	C	U	G	A	A	C	U	U	C	A	C	C	U	C	G	G
gOT-9		G	C	U	A	C	C	C	U	C	G	A	A	C	U	U	C	A	C	C	U	C	G	G
gOT-10		G	U	C	G	U	U	U	U	C	G	A	A	C	U	U	C	A	C	C	U	C	G	G

Seed region

gRNA spacer positions

Supplementary Figure S6. Schematics and sequences of gRNA spacers used to test RGN and dual nRGN specificities. (**A**) Schematics of gRNA and spacer structures. A typical gRNA with a 20-mer spacer containing the PAM proximal 10-mer seed region marked. The light and dark orange portions correspond to the CRISPR RNA (crRNA) and trans-activating crRNA (tracrRNA) moieties, respectively. A spacer without mismatches to a protospacer target sequence (gEGFP.2) is drawn in relation to a panel of spacers with mismatches (asterisks) to the protospacer (gOT1 through gOT-10). (**B**) Spacer sequences. The nucleotide sequences of spacers of gRNAs gOT-1 through gOT-10 containing 1-nt, 2-nt or 3-nt mismatches (marked in grey boxes) to a protospacer sequence (marked in green). The canonical 20-mer gRNA spacer positions and respective 10-mer seed positions are demarkated. DNA-gRNA mismatches locating within the seed region frequently have a more detrimental impact on RGN activity than those located outside this PAM-proximal region. PAM, protospacer adjacent motif.

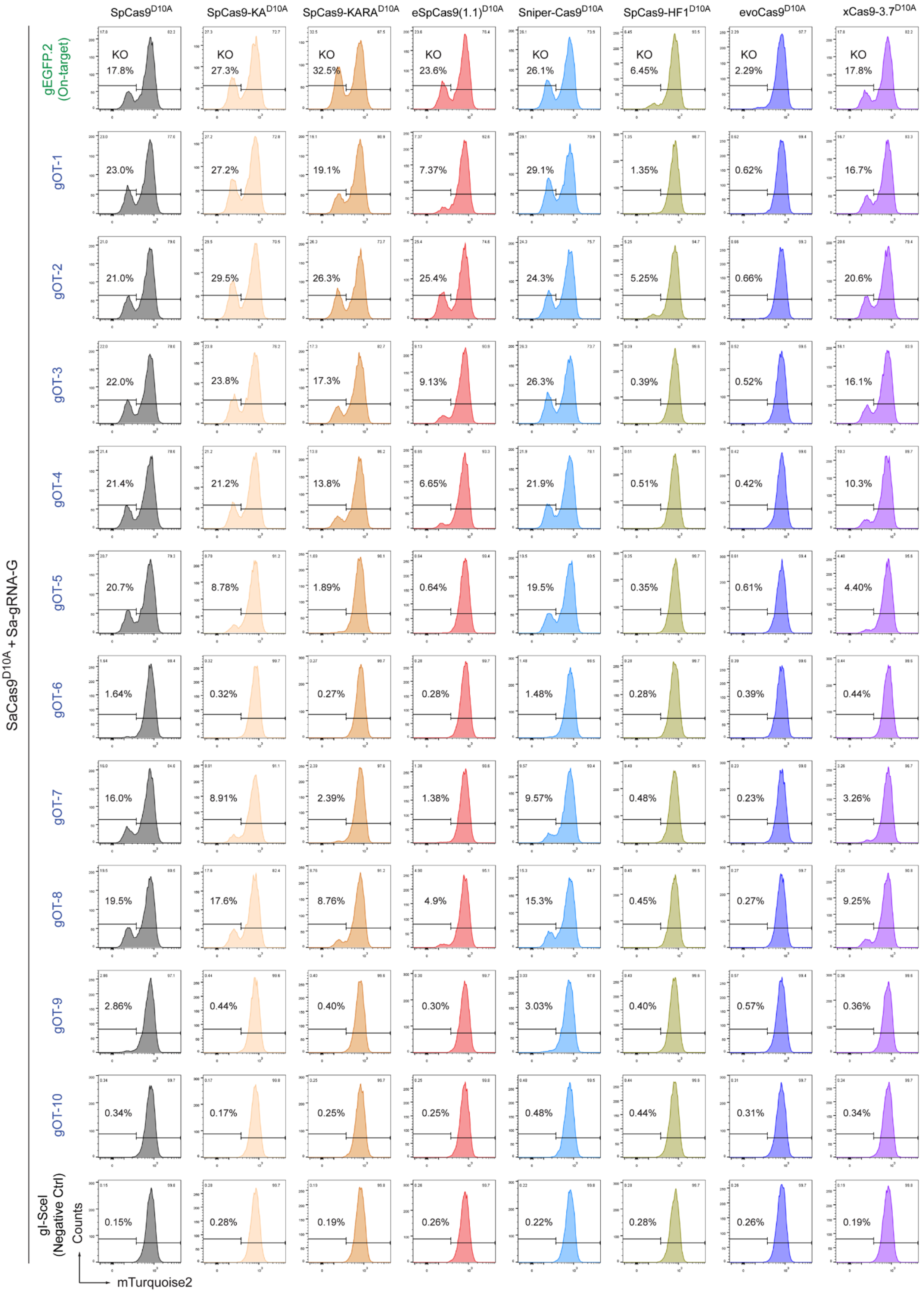

Supplementary Figure S7. Comparing the specificity profiles of nicking RGNs with SpCas9^{D10A} or different SpCas9^{D10A} variants. Representative histograms of reporter cells transfected with plasmids encoding the indicated nickases and target-site matching (gEGFP.2) or mismatching gRNAs (gOT-1 through gOT-10). A non-targeting gRNA served to establish background fluorescence levels (g-IsceI). The cumulative data is presented in **Figure 3B**.

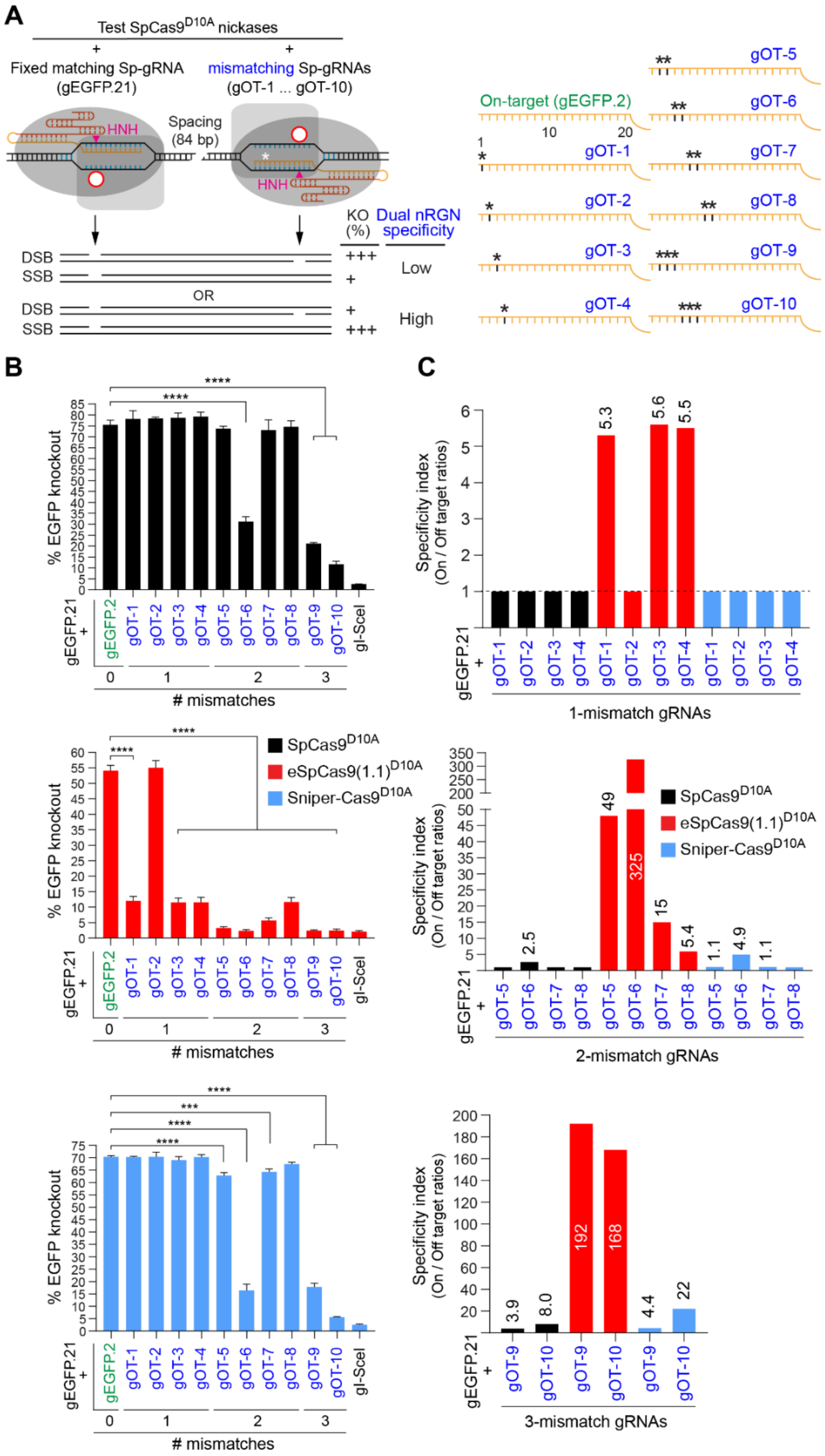

Supplementary Figure S8. Determining the specificity profiles of dual nRGNs based on standard versus high-specificity nickases. (**A**) Gene knockout assay for determining the specificity of dual nRGNs. Test *S. pyogenes* nickases were delivered together with a fixed and a varying gRNA with spacers complementary (matching) and partially complementary (mismatching), respectively, to a bipartite reporter-encoding sequence. Coordinated formation of SSBs at opposite DNA chains by

each nicking complex yields DSB-induced gene knockouts. Test nickase specificities are inversely proportional to gene knockout frequencies. The matching spacer of gEGFP.2 is drawn in relation to gRNA spacers with 1-nt (gOT-1 through gOT-4), 2-nt (gOT-5 through gOT-8) or 3-nt mismatches (gOT-9 and gOT-10) outside the seed region (asterisks). (**B**) Comparing the specificity profiles of nRGNs with SpCas9^{D10A}, eSpCas9(1.1)^{D10A} or Sniper-Cas9^{D10A}. Reporter cells were transfected with plasmids encoding the denoted nRGNs. Gene knockout levels were determined at 10 days post-transfection through flow cytometry of EGFP-negative cells and normalized for initial transfection efficiencies on per sample basis by DsRed-directed flow cytometry at 3 days post-transfection. Datasets correspond to mean ± S.D. of a minimum of three independent biological replicates. Significance between the indicated datasets was calculated with one-way ANOVA followed by Tukey's test for multiple comparisons; ***0.0001 < P < 0.001; ****P < 0.0001. (**C**) Specificity indexes of dual nRGNs based on SpCas9^{D10A}, eSpCas9(1.1)^{D10A} or Sniper-Cas9^{D10A}. The specificity indexes corresponding to DNA cleavage frequencies triggered by dual nRGNs with *EGFP*-matched gEGFP.2 divided by those induced with *EGFP*-mismatched gRNAs gOT-1 through gOT-10, are plotted. The statistically significant dual nRGN specificity indexes are depicted above or within the respective bars.

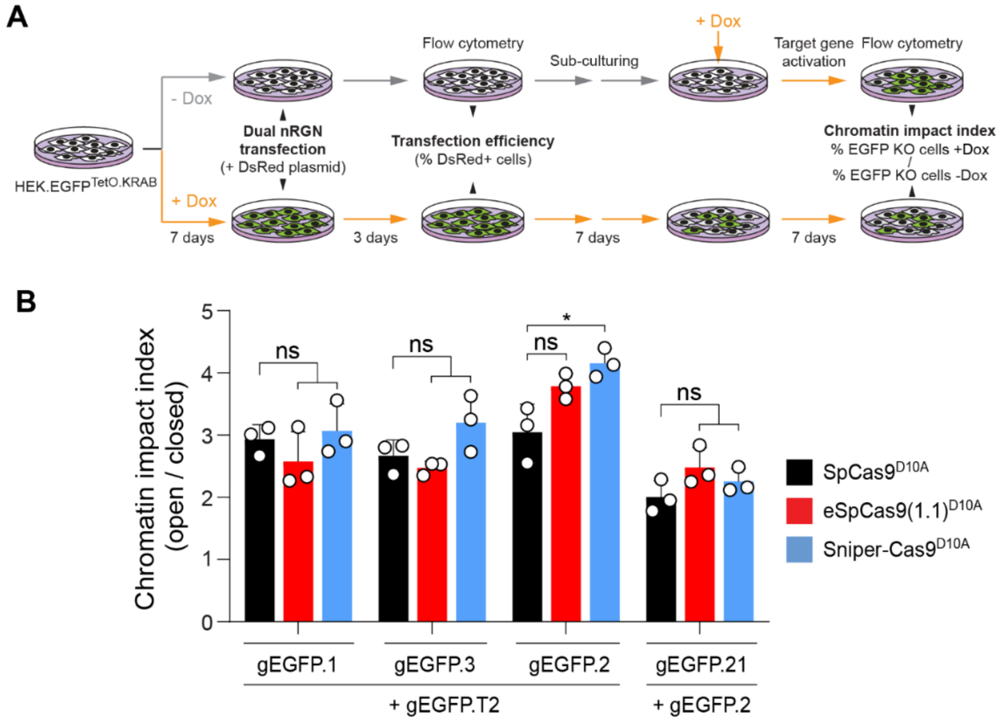

Supplementary Figure S9. Testing the impact of alternate chromatin conformations on standard versus high-specificity dual nRGNs. (**A**) Schematics of the experimental design. The tTR-KRAB-expressing HEK.EGFP$^{TetO.KRAB}$ cells permit quantifying gene-editing events at *EGFP* sequences subjected to different epigenetic states. This is achieved via a doxycycline (Dox)-dependent control over the recruitment of endogenous epigenetic silencing apparatuses. HEK.EGFP$^{TetO.KRAB}$ cells cultured with Dox (*EGFP* "open") or without Dox (*EGFP* "closed") are initially transfected with plasmids expressing different sets of dual nRGN components. A DsRed-expressing construct is included in each transfection mixture to internally control for DNA delivery efficiency. After the action of specific dual nRGNs is completed in each of the two parallel experimental settings (i.e. -Dox and +Dox), EGFP expression is activated in the -Dox setting by Dox addition permitting comparing dual nRGN-induced gene knockout frequencies at euchromatin versus heterochromatin. (**B**) Chromatin impact indexes of dual nRGNs based on SpCas9^{D10A}, eSpCas9(1.1)^{D10A} and Sniper-Cas9^{D10A}. The various chromatin impact indexes were established by calculating the ratios between *EGFP* knockout levels determined in the presence versus those measured in the absence of Dox (solid and open bars, respectively, in **Figures 5B**, **5C** and **5D**). The results are shown as mean ± S.D. of independent biological replicates (n=3). Significance amongst the different datasets was calculated using one-way ANOVA followed by Dunnett's test for multiple comparisons; *0.01< P < 0.05; P≥0.05 was considered non-significant (ns).

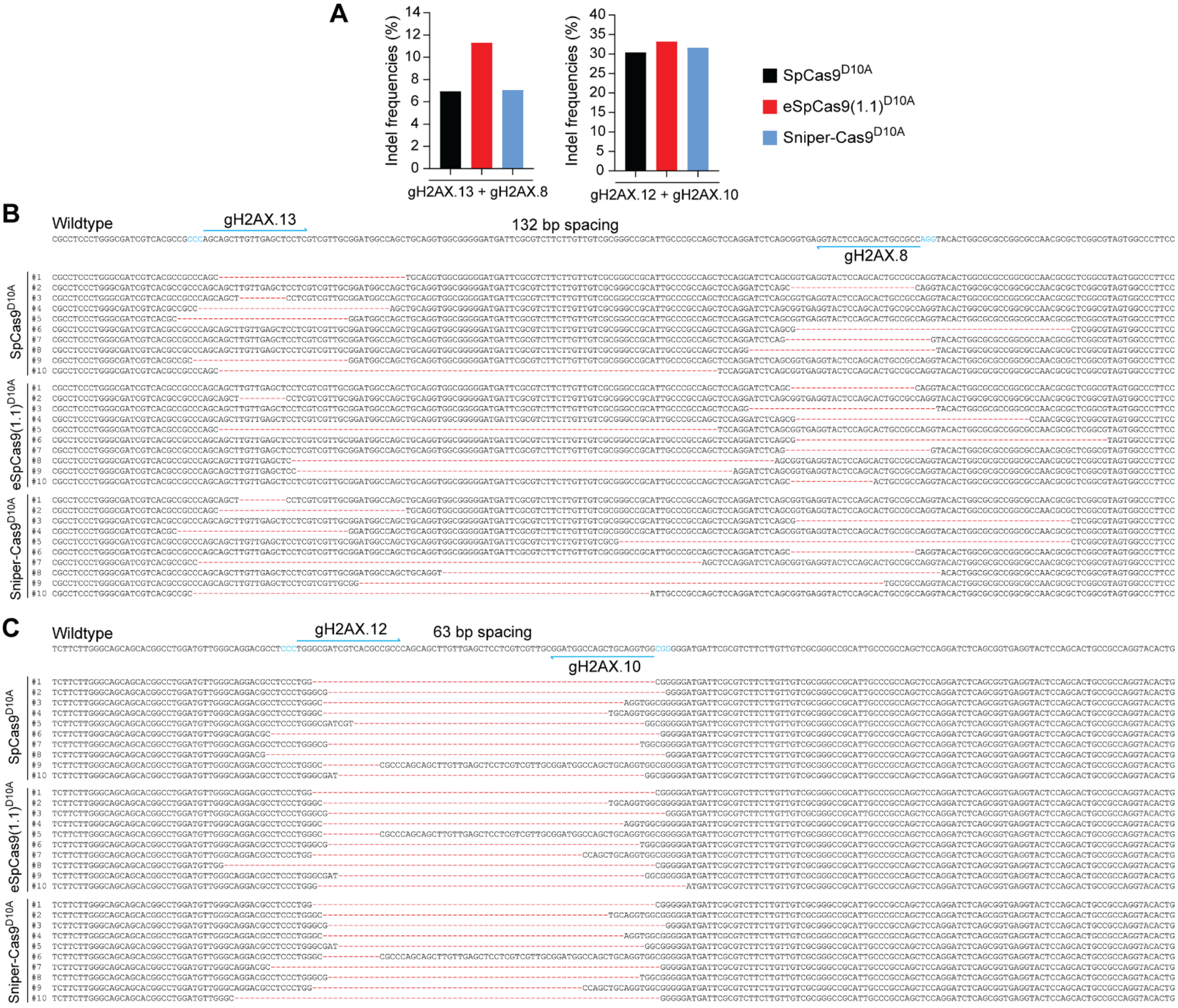

Supplementary Figure S10. Characterization of dual nRGN "footprints" at *H2AX* by deep sequencing analysis. (**A**) Cumulative indel frequencies detected in HEK293T cells treated with the indicated dual nRGNs and top 10 most frequent "footprints" induced by the indicated Cas9^{D10A} nickases coupled to (**B**) gRNA pair gH2AX.13/gH2AX.8 or (**C**) gRNA pair gH2AX.12/gH2AX.10.

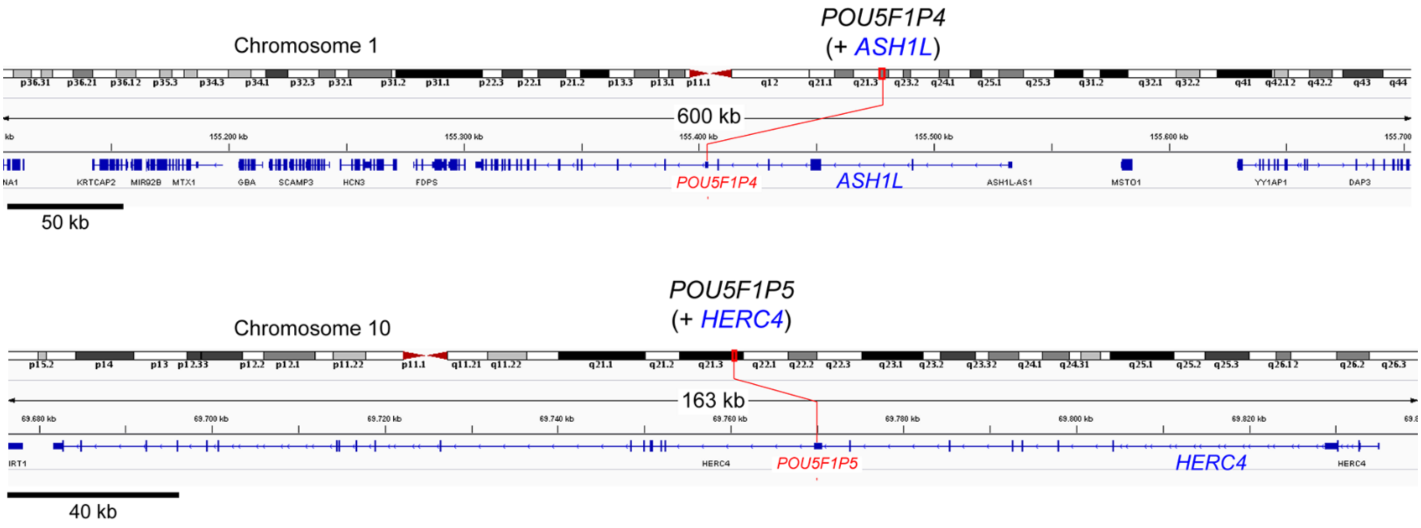

Supplementary Figure S11. Genomic coordinates of *OCT4* pseudogenes *POU5F1P4* and *POU5F1P5*. *POU5F1P4* and *POU5F1P5* are embedded within *ASH1L* (ASH1-like histone lysine methyltransferase) and *HERC4* (HECT and RLD domain containing E3 ubiquitin protein ligase 4), respectively. *ASH1L* encodes a transcriptional activator from the trithorax group and is expressed in more than 25 tissues; *HERC4* encodes a HERC ubiquitin ligase family member and is expressed in more than 25 tissues.

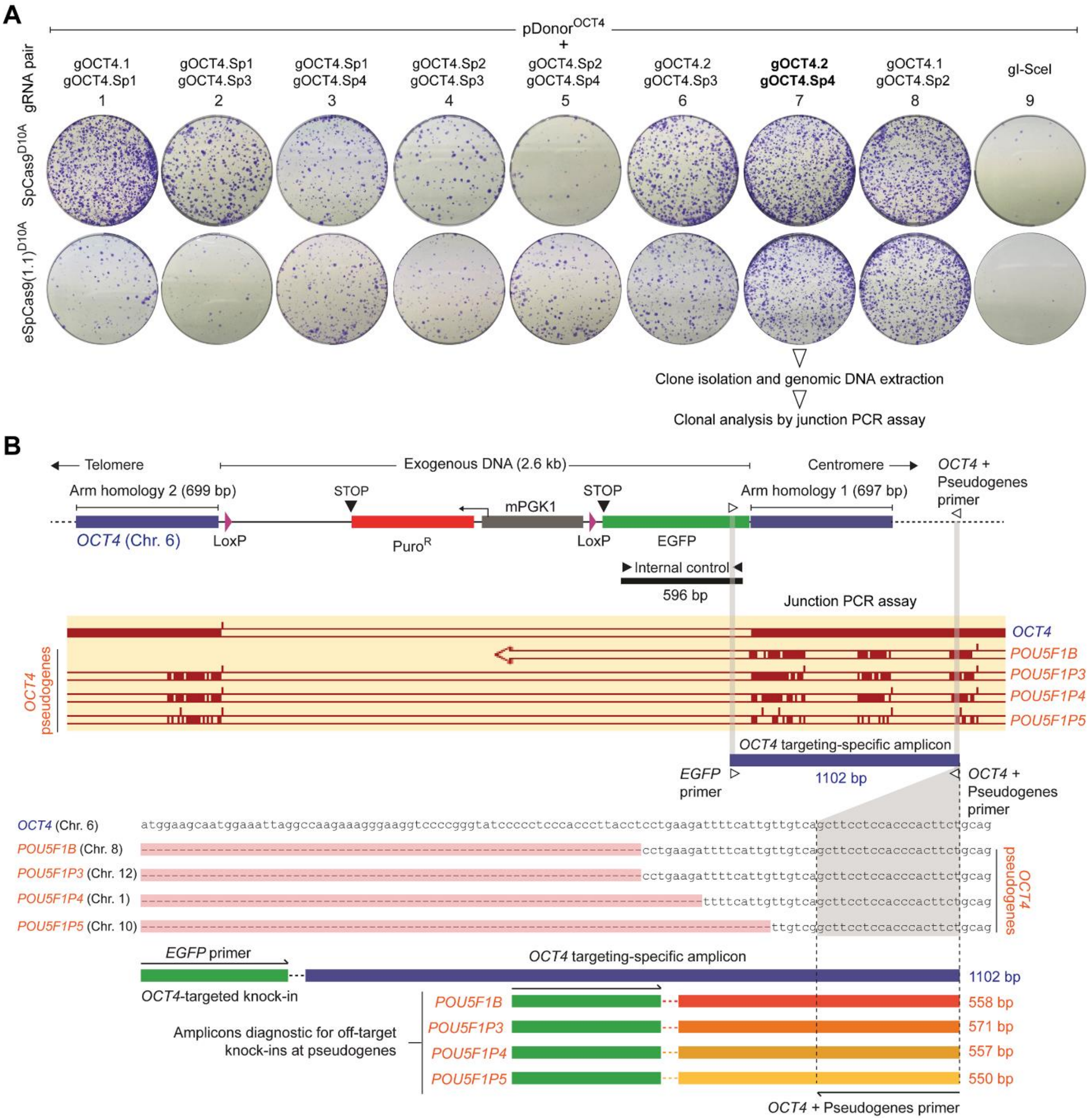

Supplementary Figure S12. Clonal analysis for assessing gene knock-ins at *OCT4* and *OCT4* pseudogene off-target sequences. (**A**) Colony-formation assays. HeLa cells genetically modified through the delivery of the indicated gene-editing tools were identified after puromycin selection and crystal violet staining. (**B)** Junction PCR assay for assessing the specificity of HDR-mediated *OCT4* gene editing. The assay combines a *EGFP*-specific primer with a primer that recognizes *OCT4*, *POU5F1P5*, *POU5F1P4*, *POU5F1B* and *POU5F1P3*. Homology regions between *OCT4* and *OCT4* pseudogenes are indicated as filled boxes. PCR products diagnostic for HDR-derived junctions between donor DNA and *OCT4* and between donor DNA and the different *OCT4* pseudogenes are shown. The *OCT4* gene knock-in specificity achieved by dual nRGNs consisting of SpCas9^{D10A} or eSpCas9(1.1)^{D10A} and gRNA pair gOCT4.2/gOCT4.Sp4, is presented in **Figure 9D**.

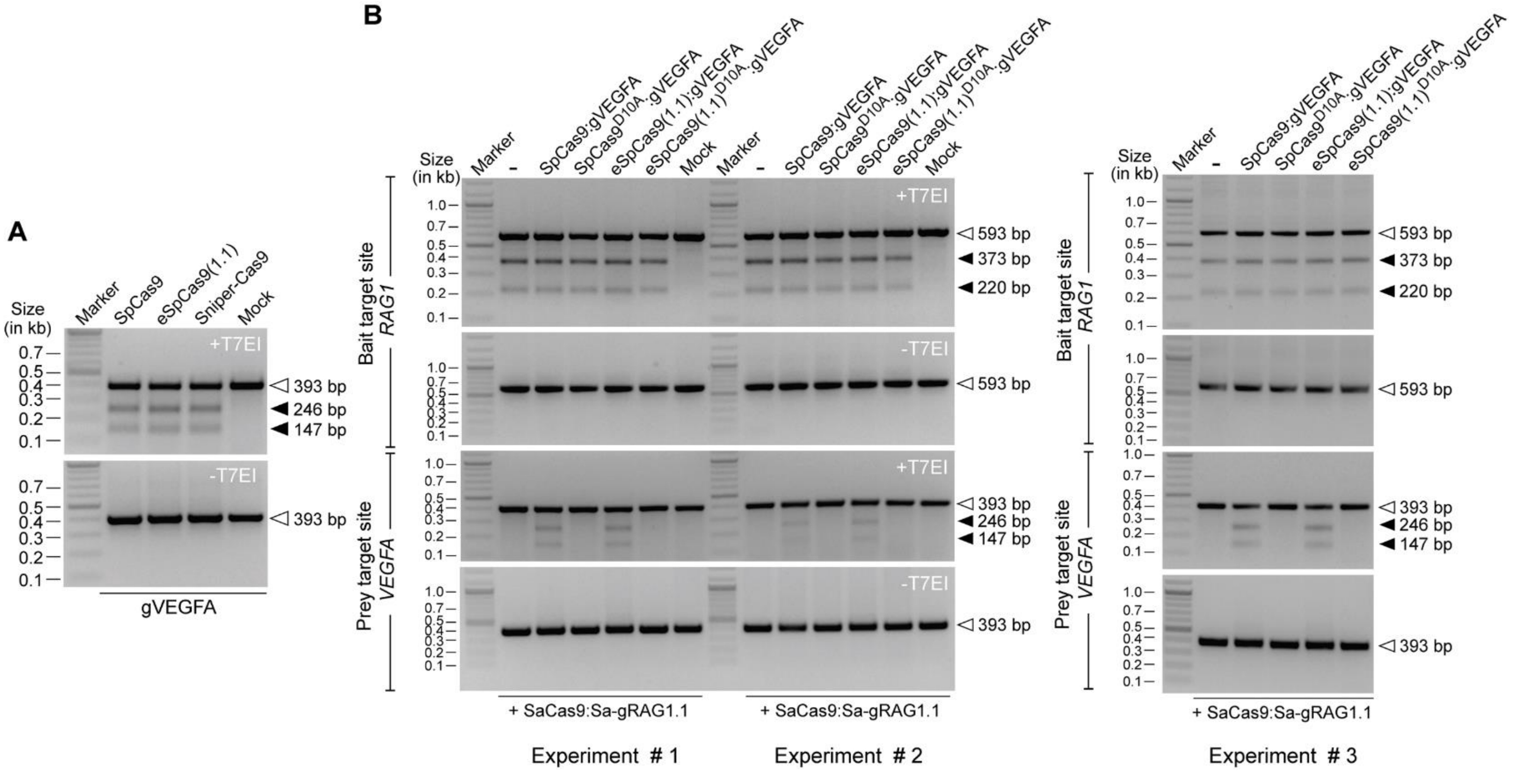

Supplementary Figure S13. Detection of targeted indels in genomic DNA used for the orthogonal HTGTS analysis. (**A**) Validation of high-specificity RGNs containing a promiscuous gRNA. HEK293T cells were transfected with constructs encoding gVEGFA and either SpCas9, eSpCas9(1.1) or Sniper-Cas9. HEK293T cells transfected only with the promiscuous gRNA gVEGFA were used as the negative control. Target site genotyping assays were carried out on genomic DNA isolated at 3 days post-transfection. T7EI-specific products resulting from indels established after NHEJ-mediated DSB repair at *VEGFA*, are marked by solid arrowheads; amplicons corresponding to intact *VEGFA* are instead marked by open arrowheads. Marker, GeneRuler DNA Ladder Mix molecular weight marker. (**B**) Assessing targeted chromosomal cleavage in DNA for orthogonal HTGTS analysis. HEK293T cells were transfected with plasmids expressing the indicated combinations of proteins and gRNAs (n=3 independent biological replicates). At 2 days post-transfection, genomic DNA was subjected to T7EI assays for assessing DNA cleavage at *RAG1* induced by SaCas9:Sa-gRAG1.1 complexes (universal bait DSBs) and at *VEGFA* triggered by test nucleases and test nickases (prey DSBs). Test nucleases consisted of SpCas9:gVEGFA and eSpCas9(1.1):gVEGFA complexes; test nickases consisted of SpCas9^{D10A}:gVEGFA and eSpCas9(1.1)^{D10A}:gVEGFA complexes. Open and solid arrowheads locate *RAG1* and *VEGFA* amplicons resistant and susceptible to the mismatch sensing T7EI endonuclease, respectively. The former and latter DNA species correspond to intact and cleaved target sequences, respectively. Negative controls were derived from mock-transfected HEK293T cells and from amplicons not treated with T7EI (-T7EI). Marker, GeneRuler DNA Ladder Mix molecular weight marker.

	Chromosomes	Enriched Translocation Sites (hg38 reference)		**SaCas9:Sa-gRAG1.1			**SaCas9:Sa-gRAG1.1 + SpCas9:gVEGFA			**SaCas9:Sa-gRAG1.1 + eSpCas9(1.1):gVEGFA			**SaCas9:Sa-gRAG1.1 + SpCas9D10A:gVEGFA			**SaCas9:Sa-gRAG1.1 + eSpCas9(1.1)D10A:gVEGFA			Target Sequence PAM	Mismatches
				Exp#1 ◦23181	Exp#2 ◦19374	Exp#3 ◦12987	Exp#1 ◦22165	Exp#2 ◦18660	Exp#3 ◦13675	Exp#1 ◦22169	Exp#2 ◦20086	Exp#3 ◦13127	Exp#1 ◦23629	Exp#2 ◦12156	Exp#3 ◦11932	Exp#1 ◦20756	Exp#2 ◦15480	Exp#3 ◦13914		
	Bait *SaCas9+Sa-gRAG1.1*																		consensus PAM NNGRRT	
	*chr11	36,562,703	36,585,089	N/A	N/A	N/A	N/A	N/A	N/A	N/A	N/A	N/A	N/A	N/A	N/A	N/A	N/A	N/A	GGGCAGAACTGAGTCCCAAG GTGGGT	0
	chr1	54,580,205	54,581,836	**1.85**	**1.6**	**3.31**	**0.59**	**0.48**	**1.17**	**0.9**	**0.85**	**1.22**	**1.14**	**0.9**	**1.84**	**0.67**	**1.16**	**2.66**	GGGCAGAGCAAAGTCCCATG GTGGGT	4
	chr8	98,304,549	98,305,557	**1.08**	**0.93**	**1.08**	**0.32**	**0.38**	**0.66**	**0.59**	**0.55**	**0.38**	**0.59**	**0.74**	0.34	**0.77**	**0.65**	**0.36**	GGGCAGGCCTGAGTCCCAAG AAGAGC	3
	chr8	71,976,446	71,977,550	**0.86**	**1.24**	**3**	**0.27**	0.16	**1.61**	**0.32**	**0.4**	**1.3**	**0.68**	**0.41**	**1.84**	**0.48**	**0.45**	**1.65**	GAGCAGAGCCAAGTCCCAAG CTGAGC	5
	chr11	22,773,677	22,774,176	**0.13**	0	0.23	0.05	0	0	0.09	0	0.08	0.08	0	0	0.05	0	0	CATAGCAAATAAGCCCCAAG GAGAGT	9
	Prey *SpCas9+Sp-gVEGFA*																		consensus PAM NGG	
	*chr6	43,765,755	43,774,428	0.04	0.05	0	**30.23**	**21.97**	**24.2**	**32.52**	**27.18**	**28.34**	**2.62**	**1.56**	**2.43**	**1.64**	**1.29**	**1.65**	GGTGAGTGAGTGTGTGCGTG TGG	0
Hotspots	chr22	37,264,738	37,268,149	0	0	0	21.25	**16.4**	**32.47**	0.09	0.25	0.15	0	0	0	0	0	0	GCTGAGTGAGTGTATGCGTG TGG	2
	***chr11	69,081,544	69,085,720	0	0	0	**16.15**	**12.11**	**24.64**	0.32	**0.5**	0.3	0	0	0	0	0	0	GGTGAGTGAGTGCGTGCGGG TGG<br>AGTGGGTGAGTGAGTGCGTG CGG	2<br>3
	chr5	116,096,784	116,099,773	0	0	0	**13.85**	**11.58**	**17.11**	0.05	0	0.08	0	0	0	0	0	0	TGTGGGTGAGTGTGTGCGTG AGG	2
	chr14	65,100,038	65,104,160	0	0	0	**9.79**	**5.95**	**7.61**	**11.95**	**12.45**	**12.34**	0.17	0.33	0	0.05	0.06	0.22	AGTGAGTGAGTGTGTGTGTG GGG	2
	chr5	90,144,288	90,146,443	0	0	0	**8.57**	**5.14**	**11.12**	0.05	0	0	0	0	0	0	0	0	AGAGAGTGAGTGTGTGCATG AGG	3
	chr20	20,196,676	20,198,171	0	0	0	**1.76**	**2.04**	**4.02**	0	0	0	0	0	0	0	0	0	AGTGTGTGAGTGTGTGCGTG TGG	2
	chr3	194,275,428	194,276,869	0	0	0	**1.71**	**1.39**	**3.88**	0	0.05	0	0	0	0	0	0	0	AGTGAATGAGTGTGTGTGTG TGG	3
	chr14	105,561,675	105,563,712	0	0	0	**1.4**	**1.71**	**2.56**	**4.06**	**3.83**	**5.79**	**0.25**	0.25	0.17	0.05	0.06	0.14	GGTGAGTGAGTGTGTGTGTG AGG	2
	chr10	97,000,122	97,001,773	0	0	0	**1.26**	**0.7**	**2.34**	0	0	0	0	0	0	0	0	0	GTTGAGTGAATGTGTGCGTG AGG	2
	chr11	7,604,064	7,605,063	0	0	0	**1.17**	**0.59**	**0.59**	0	0	0	0	0	0	0	0	0	GGTGAGTAGGTGTGTGTGTG GGG	3
	chr12	6,827,391	6,828,485	0	0	0	**1.13**	**0.91**	**0.95**	0	0	0	0	0	0	0	0	0	GGTGGATGAGTGTGTGTGTG GGG	3
	chr14	73,886,294	73,887,293	0	0	0	**0.95**	**0.54**	**0.73**	0	0	0	0	0	0	0	0	0	AGCGAGTGGGTGTGTGCGTG GGG	3
	chr14	61,611,555	61,612,554	0	0	0	**0.81**	**0.48**	**0.73**	0	0	0	0	0	0	0	0	0	TGTGAGTAAGTGTGTGTGTG TGG	3
	chr19	40,055,460	40,056,459	0	0	0	**0.63**	**0.43**	**0.66**	0	0	0	0	0	0	0	0	0	ACTGTGTGAGTGTGTGCGTG AGG	3
	chr9	23,824,056	23,825,055	0	0	0	**0.54**	**0.43**	**0.73**	0	0	0	0	0	0	0	0	0	TGTGGGTGAGTGTGTGCGTG AGA	3
	chr8	48,084,746	48,085,744	0	0	0	**0.54**	**0.54**	**0.44**	0	0	0	0	0	0	0	0	0	GTAGAGTGAGTGTGTGTGTG TGG	3
	chr20	52,107,366	52,108,363	0	0	0	**0.32**	0.21	**0.51**	0	0.05	0	0	0	0	0	0	0	CGTGAGTGAGTGTGTACCTG GGG	3
	chr9	18,733,137	18,734,133	0	0	0	**0.27**	**0.38**	**0.44**	0	0	0	0	0	0	0	0	0	AGCGAGTGAGTGTGTGTGTG GGG	3
	chr8	23,074,486	23,075,480	0	0	0	0.18	0.05	0.07	**0.45**	**0.25**	**0.46**	0	0	0	0	0	0	AGTGAGTGTGTGTGTGTGAG AGA	5
Enriched Sites	chr11	79,466,979	79,467,978	0	0	0	**0.54**	0.27	0.22	0.05	0.15	0.15	0	0.08	0	0	0	0	AGTGAGTGAGTGAGTGAGTG GGG	3
	chr19	6,108,522	6,109,520	0	0	0	**0.41**	0.21	0.29	0	0	0	0	0	0	0	0	0	TGTGAGTGAGTGTGTGTGTG TGA	3
	chr22	49,343,576	49,344,575	0	0	0	0.36	0.16	**0.51**	0	0	0	0	0	0	0	0	0	GGTGTGTGAGTGTGTGTGTG TGG	2
	chr4	61,201,893	61,202,400	0	0	0	**0.36**	0.16	**0.22**	0	0	0	0	0	0	0	0	0	GATGAGTGTGTGTGTGTGTG AGG	3
	chr19	16,458,177	16,459,175	0	0	0	**0.32**	0.21	0.29	0	0	0	0	0	0	0	0	0	TGTGAGTGAGTGTGTGTGTG GAG	3
	chr2	176,598,201	176,599,197	0	0	0	**0.32**	0.16	0.15	0	0	0	0	0.08	0	0	0	0	GGTGAGTGTGTGTGTGCATG TGG	2
	chr16	74,863,723	74,864,721	0	0	0	**0.27**	0.05	0.15	0	0	0	0	0	0	0	0	0	GGTGAGAGAGTGTGTGCGTA GGA	3
	chr11	63,598,860	63,599,369	0	0	0	**0.23**	0.11	0.07	0	0	0	0	0	0	0	0	0	ATTGAGTGAGTATGTGTGTG AGG	4
	chr6	156,756,693	156,757,194	0	0	0	**0.23**	0.05	0.15	0	0	0	0	0	0	0	0	0	GATGAGTGAGTGAGTGAGTG GGG	3
	chr10	128,429,590	128,430,589	0	0	0	0.18	0.27	**0.73**	0	0	0	0	0	0	0	0	0	GGGGAGTGACTGTGTGCGTG TGG	2
	chr11	69,271,733	69,272,718	0	0	0	0.09	0.16	**0.66**	0	0	0	0	0	0	0	0	0	GGTGGATGAGTGAATGCGTG AGG	4
	chr16	83,998,559	83,999,540	0	0	0	0.05	0.16	**0.29**	0	0.05	0	0	0	0	0	0	0	GGTGAATGAGTGTGTGCTCT GGG	4

◦ indicates the number of junctions in each library

* Predicted target sites for S.aureus and S.pyogenes CRISPR complexes at chromosomes 11 and 6 respectively

** Translocation junctions frequencies are per 1000 junctions within each individual library ; frequencies in bold are statistically significant (MACS2; q-value cutoff -10^-10)

*** 2 off-targets were found in this hotspot region: the first one seems is mainly used in presence of eSpCas9(1.1), while the second one is mainly used in presence of SpCas9

Supplementary Figure S14. Genome-wide off-target effects induced by cleaving and nicking RGNs harboring a promiscuous gRNA. Description of the chromosomal coordinates of hotspots and enriched translocation sites. Enriched sites are off-target sites found significant in at least one of the total libraries; hotspots are defined as enriched sites found significant in at least 2 out of 3 normalized libraries for each CRISPR set. The frequencies of translocation junctions per 1000 junctions within each individual library and the on-target and off-target sequences of *S. aureus* and *S. pyogenes* CRISPR complexes used in the orthogonal HTGTS analyses, are indicated (n=3 independent biological replicates). Orthogonal HTGTS analyses was performed on genomic DNA from HEK293T cells exposed to nucleases SpCas9:gVEGFA and eSpCas9(1.1):gVEGFA or to nickases SpCas9^{D10A}:gVEGFA and eSpCas9(1.1)^{D10A}:gVEGFA (**Figures 12** and **13**). Translocation junction frequencies in bold are statistically significant (MACS2; q-value cutoff -10^-10).

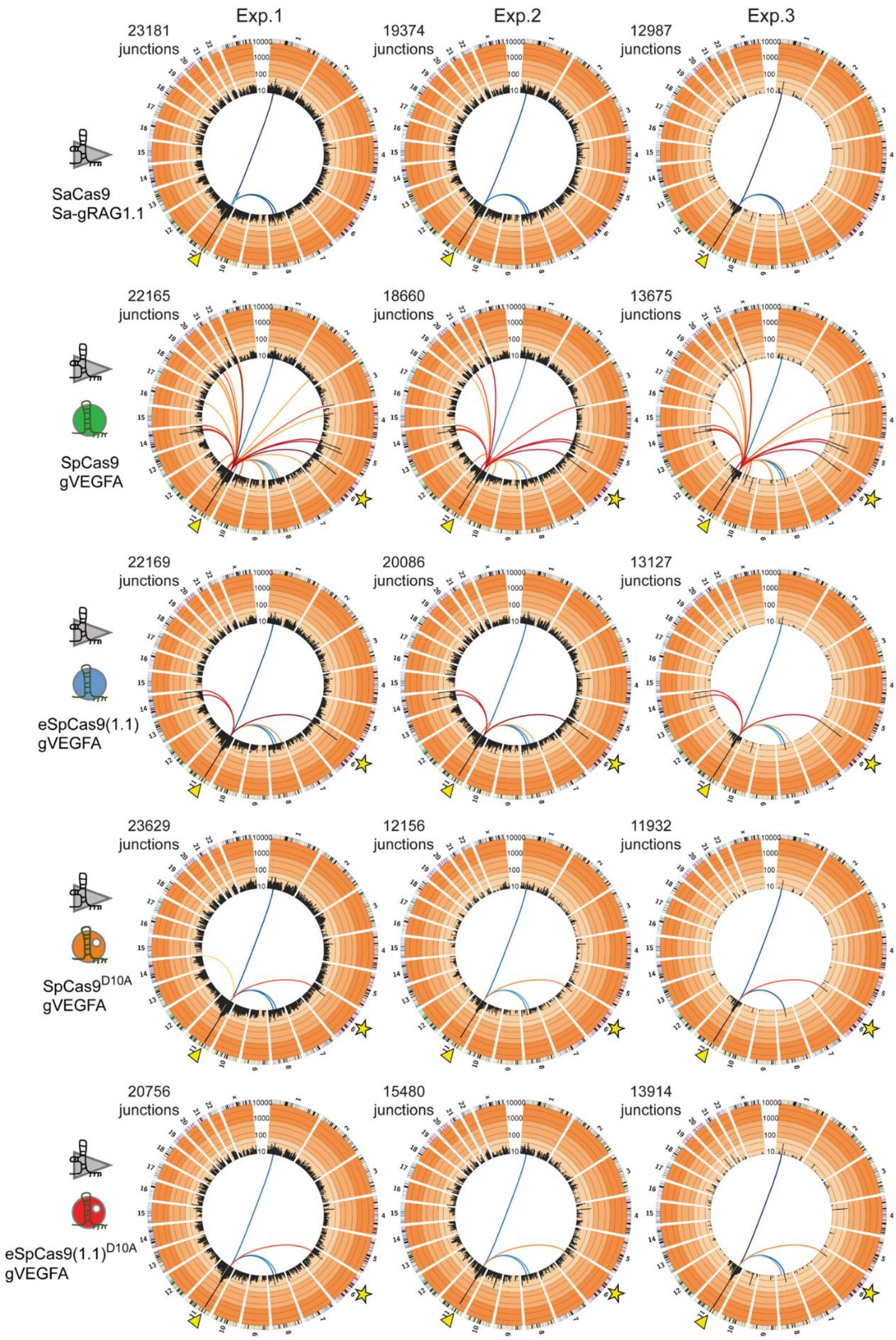

Supplementary Figure S15. Orthogonal HTGTS analyses of off-target effects triggered by cleaving versus nicking RGNs. Individual circos plots from three independent biological replicates of HTGTS sequence read libraries from HEK293T cells. HEK293T cells were transfected with constructs expressing the indicated RGNs and nRGNs. At 2 days post-transfection, orthogonal HTGTS analyses were carried out on genomic DNA previously screened by target-site genotyping assays (**Supplementary Figure S13B**). For a description of the various icons see legend of **Figure 12**. Junctions obtained from total libraries are shown, the number of junctions in each library is indicated. Hotspots are established when significantly enriched translocation sites are present in the library (MACS2; q-value cutoff -10^-10).

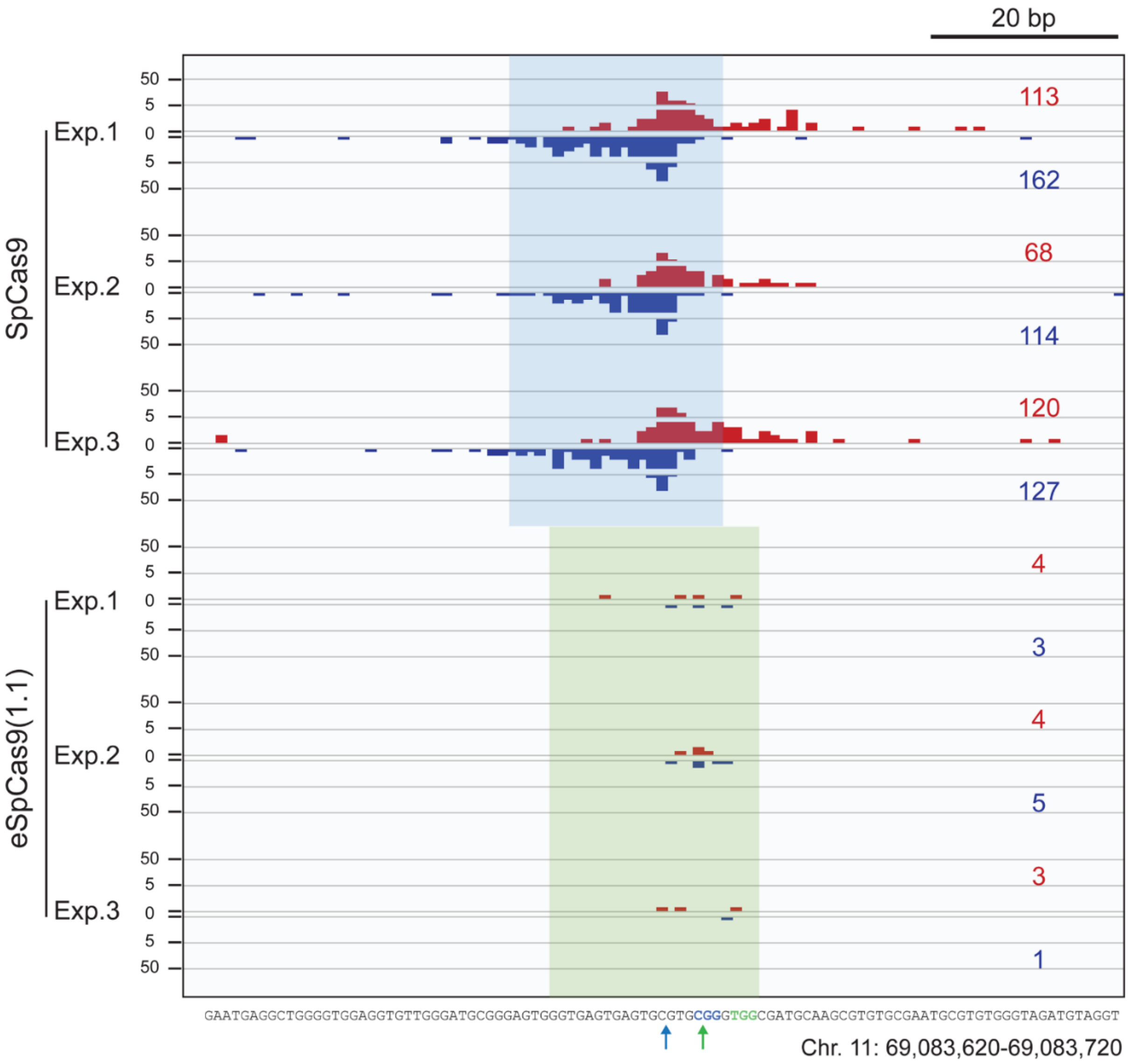

Supplementary Figure S16. Differential translocation enrichment to adjacent chromosome 11 off-target sites for SpCas9 and eSpCas9(1.1) nucleases. IGV plots are shown for SpCas9 and eSpCas9(1.1) overlapping off-target sites identified within the enriched translocation region on chr11: 69,081,544-69,085,720. Junctions are displayed as stacked tracks (i.e., log scale between tracks; linear scale within each track). Red and blue numbers indicate the number of junctions from this region that translocated in the plus and minus orientation, respectively. The shaded blue and green rectangles indicate the locations of the off-target sites (AGTGGGTGAGTGAGTGCGTG<u>CGG</u> and GGTGAGTGAGTGCGTGCGGG<u>TGG</u>) that seem mostly engaged by SpCas9:gVEGFA and eSpCas9(1.1):gVEGFA complexes, respectively. The blue and green arrows indicate the expected cutting sites within each off-target site described in the **Supplementary Figure S14**.
