# Supplementary material for: Precise and broad scope genome editing based on high-specificity Cas9 nickases
Source: Nucleic Acids Res. 2021 Jan 4;49(2):1173–98. doi: 10.1093/nar/gkaa1236 (PMC7826261; doi:10.1093/nar/gkaa1236)
Supplement: gkaa1236_Supplemental_Files [file gkaa1236_supplemental_files.zip › Wang et al. Supplementary Tables S1-S42.pdf]

Supplementary Table S1. Overview of the plasmids used for expressing the different SpCas9 nucleases and respective SpCas9^{D10A} nickases.

Plasmid codes	Encoded products
AV62_pU.CAG.Cas9.rBGpA	SpCas9
AB65_pU.CAG.Cas9-D10A.rBGpA	SpCas9^{D10A}
AW01_pU.CAG.Cas9-eSp(1.1).rBGpA	eSpCas9(1.1)
AA69_pU.CAG.Cas9-eSp(1.1)-D10A.rBGpA.2NLS	eSpCas9(1.1)^{D10A}
AP75_pU.CAG.Cas9-K848A.rBGpA	SpCas9-KA
AP76_pU.CAG.Cas9-D10A-K848A.rBGpA	SpCas9-KA ^{D10A}
AP69_pU.CAG.Cas9-K848A-R1060A.rBGpA	SpCas9-KARA
AP70_pU.CAG.Cas9-D10A-K848A-R1060A.rBGpA	SpCas9-KARA ^{D10A}
AE69_pU.CAG.SniperCas9.rBGpA	SniperCas9
AE70_pU.CAG.SniperCas9-D10A.rBGpA	SniperCas9^{D10A}
AV64_pU.CAG.Cas9-HF1.rBGpA	SpCas9-HF1
BB37_pU.CAG.Cas9-HF1-D10A.rBGpA	SpCas9-HF1^{D10A}
AP73_pU.CAG.Cas9-evo.rBGpA	evoCas9
AP74_pU.CAG.Cas9-evo-D10A.rBGpA	evoCas9^{D10A}
AT82_pU.CAG.xCas9-3.6.rBGpA	xCas9-3.6
AT83_pU.CAG.xCas9-3.6-D10A.rBGpA	xCas9-3.6^{D10A}
AT84_pU.CAG.xCas9-3.7.rBGpA	xCas9-3.7
AT85_pU.CAG.xCas9-3.7-D10A.rBGpA	xCas9-3.7^{D10A}

Supplementary Table S2. Oligonucleotides used for gRNA assembly and corresponding target genes.

Plasmid name	Oligonucleotide code	Oligonucleotide sequence (5' → 3')	Target site
BB14_pU6.gTURQ.1	#275	ACCGTTGTGGCGGATCTTGAAGT	*mTurquoise2*
	#276	AAACACTTCAAGATCCGCCACAA	
BB14_pU6.gTURQ.2	#277	ACCGCGCCACAACATCGAGGACGG	*mTurquoise2*
	#278	AAACCCGTCCTCGATGTTGTGGCG	
BB21_pU6.gTURQ.3	#271	ACCGCCAGGACAGGGTGGTCACGA	*mTurquoise2*
	#272	AAACTCGTGACCACCCTGTCCTGG	
BB13_pU6.gTURQ.4	#273	ACCGCTCGTGACCACCCTGTCCTG	*mTurquoise2*
	#274	AAACCAGGACAGGGTGGTCACGAG	
AM51_pU6.gI-SceI	#25	ACCGGTGAGCTCTTATTTGCGTAGCTAGCTGAC	*I-SceI*
	#26	AAACGTCAGCTAGCTACGCAAATAAGAGCTCAC	
AV58_pU6.gEGFP.1	#159	ACCGGGCACGGGCAGCTTGCCGG	*EGFP*
	#160	AAACCCGGCAAGCTGCCCGTGCC	
AV59_pU6.gEGFP.2	#161	ACCGTCGCCCTCGAACTTCACCT	*EGFP*
	#162	AAACAGGTGAAGTTCGAGGGCGA	
AV60_pU6.gEGFP.3	#163	ACCGTAGGTCAGGGTGGTCACGA	*EGFP*
	#164	AAACTCGTGACCACCCTGACCTA	
AW16_pU6.gEGFP.6.tru17	#169	ACCGAGGGCGATGCCACCTA	*EGFP*
	#170	AAACTAGGTGGCATCGCCCT	
AW18_pU6.gEGFP.6.tru19	#171	ACCGCGAGGGCGATGCCACCTA	*EGFP*
	#172	AAACTAGGTGGCATCGCCCTCG	
AW20_pU6.gEGFP.6.FL20	#173	ACCGGCGAGGGCGATGCCACCTA	*EGFP*
	#174	AAACTAGGTGGCATCGCCCTCGC	
AW22_pU6.gEGFP.7	#175	ACCGACCAGGATGGGCACCACCC	*EGFP*
	#176	AAACGGGTGGTGCCCATCCTGGT	

BB10_pU6.SagRNAeGFP.G	#289	CACCGCAACATCCTGGGGCACAAGC	*EGFP*
	#290	AAACGCTTGTGCCCCAGGATGTTGC	
BB11_pU6.gEGFP.21	#291	ACCGCAACATCCTGGGGCACAAGC	*EGFP*
	#292	AAACGCTTGTGCCCCAGGATGTTG	
AE71_ pU6.opt-gEGFP.OT.1	#421	ACCGATCGCCCTCGAACTTCACCT	*EGFP*
	#422	AAACAGGTGAAGTTCGAGGGCGAT	
AE72_ pU6.opt-gEGFP.OT.2	#423	ACCGCCGCCCTCGAACTTCACCT	*EGFP*
	#424	AAACAGGTGAAGTTCGAGGGCGG	
AE73_ pU6.opt-gEGFP.OT.3	#425	ACCGTTGCCCTCGAACTTCACCT	*EGFP*
	#426	AAACAGGTGAAGTTCGAGGGCAA	
AE74_ pU6.opt-gEGFP.OT.4	#427	ACCGTCACCCTCGAACTTCACCT	*EGFP*
	#428	AAACAGGTGAAGTTCGAGGGTGA	
AN65_ pU6.opt-gEGFP.OT.5	#447	ACCGCTGCCCTCGAACTTCACCT	*EGFP*
	#448	AAACAGGTGAAGTTCGAGGGCAG	
AN66_ pU6.opt-gEGFP.OT.6	#449	ACCGTCATCCTCGAACTTCACCT	*EGFP*
	#450	AAACAGGTGAAGTTCGAGGATGA	
AN67_ pU6.opt-gEGFP.OT.7	#451	ACCGTCGCTTTCGAACTTCACCT	*EGFP*
	#452	AAACAGGTGAAGTTCGAAAGCGA	
AN68_ pU6.opt-gEGFP.OT.8	#453	ACCGTCGCCCCTGAACTTCACCT	*EGFP*
	#454	AAACAGGTGAAGTTCAGGGGCGA	
AN69_ pU6.opt-gEGFP.OT.9	#455	ACCGCTACCCTCGAACTTCACCT	*EGFP*
	#456	AAACAGGTGAAGTTCGAGGGTAG	
AN70_ pU6.opt-gEGFP.OT.10	#457	ACCGTCGTTTTCGAACTTCACCT	*EGFP*
	#458	AAACAGGTGAAGTTCGAAAACGA	
AK63_pU6.opt-gEGFP27a.tru17	#517	ACCGCCCTCGAACTTCACCT	*EGFP*
	#518	AAACAGGTGAAGTTCGAGGG	
AK64_pU6.opt-gEGFP28.tru18	#519	ACCGCTCGATGCGGTTCACCA	*EGFP*
	#520	AAACTGGTGAACCGCATCGAG	

AK65_pU6.opt-gEGFP29.tru19	#521	ACCGATGCCCTTCAGCTCGATG	*EGFP*
	#522	AAACCATCGAGCTGAAGGGCAT	
AK66_pU6.opt-gEGFP30. FL20	#523	ACCGCCGTCGTCCTTGAAGAAGA	*EGFP*
	#524	AAACTCTTCTTCAAGGACGACGG	
AM17_pU6.opt-gEGFP.tru.18.OT1	#539	ACCGTTCGATGCGGTTCACCA	*EGFP*
	#540	AAACTGGTGAACCGCATCGAA	
AM18_pU6.opt-gEGFP.tru.18.OT2	#541	ACCGCCCGATGCGGTTCACCA	*EGFP*
	#542	AAACTGGTGAACCGCATCGGG	
AT69_pU6.opt-gH2AX.8	#465	ACCGGTACTCCAGCACTGCCGCC	*H2AX*
	#466	AAACGGCGGCAGTGCTGGAGTAC	
AT70_pU6.opt-gH2AX.9	#467	ACCGCTGAGATCCTGGAGCTGGC	*H2AX*
	#468	AAACGCCAGCTCCAGGATCTCAG	
AT71_pU6.opt-gH2AX.10	#469	ACCGGATGGCCAGCTGCAGGTGG	*H2AX*
	#470	AAACCCACCTGCAGCTGGCCATC	
AT72_pU6.opt-gH2AX.11	#471	ACCGCTGGCCATCCGCAACGACG	*H2AX*
	#472	AAACCGTCGTTGCGGATGGCCAG	
AT73_pU6.opt-gH2AX.12	#473	ACCGGCGGCGTGACGATCGCCCA	*H2AX*
	#474	AAACTGGGCGATCGTCACGCCGC	
AK50_pU6.opt-gH2AX.13	#513	ACCGAGGAGCTCAACAAGCTGCT	*H2AX*
	#514	AAACAGCAGCTTGTTGAGCTCCT	
AK67_pU6.opt-gH2AX.14	#531	ACCGAGGCGTCCTGCCCAACATCC	*H2AX*
	#532	AAACGGATGTTGGGCAGGACGCCT	
AK69_pU6.opt-gH2AX.15	#535	ACCGGCGTCCTGCCCAACATCC	*H2AX*
	#536	AAACGGATGTTGGGCAGGACGC	
AT75_pU6.opt-gH2AX.OT1	#477	ACCGCGGAGATCCTGGAGCTGGC	*H2AX*
	#478	AAACGCCAGCTCCAGGATCTCCG	
AT76_pU6.opt-gH2AX.OT2	#479	ACCGGATGGCGAGCTGCAGGTGG	*H2AX*
	#480	AAACCCACCTGCAGCTCGCCATC	
AT77_pU6.opt-gH2AX.OT3	#481	ACCGCTCGCCATCCGCAACGACG	*H2AX*
	#482	AAACCGTCGTTGCGGATGGCGAG	

AK68_pU6.opt-gH2AX.OT4	#533	ACCGCGGCGTCCTGCCCAACATCC	*H2AX*
	#534	AAACGGATGTTGGGCAGGACGCCG	
AX33_pU6.opt-gOCT4.1	#217	ACCGCACCTCAGTTTGAATGCAT	*OCT4*
	#218	CAAAATGCATTCAAACTGAGGTG	
AX34_pU6.opt-gOCT4.2	#219	ACCGTCTCCCATGCATTCAAACTG	*OCT4*
	#220	CAAACAGTTTGAATGCATGGGAGA	
AK51_pU6.opt-gOCT4.Sp1	#497	ACCGAAAACCTGGAGTTTGTGCC	*OCT4*
	#498	AAACGGCACAAACTCCAGGTTTT	
AK52_pU6.opt-gOCT4.Sp2	#499	ACCGGAATGGGGGACAGGGGGAG	*OCT4*
	#500	AAACCTCCCCCTGTCCCCCATTC	
AK53_pU6.opt-gOCT4.Sp3	#501	ACCGGGAGAGCCCAGAGTGGTGA	*OCT4*
	#502	AAACTCACCACTCTGGGCTCTCC	
AK54_pU6.opt-gOCT4.Sp4	#503	ACCGGTGACGGAGACAGGGGGAA	*OCT4*
	#504	AAACTTCCCCCTGTCTCCGTCAC	
AG65_pU6.opt-gVEGFA	#7352	ACCGGTGAGTGAGTGTGTGCGTG	*VEGFA*
	#7353	AAACCACGCACACACTCACTCAC	

Supplementary Table S3. Transfection scheme for assessing the activities and specificities of the different nucleases in TURQ2 cells (**Figure 1C**).

TURQ2 cells	5 ×10^{4} cells per well of 24-well plates													
	400ng DNA and 2.05 µl PEI (1 mg ml^{-1}) per well (medium replaced at 6 h post-transfection)													
Encoded products	SpCas9	SpCas9-KA	SpCas9-KARA	eSpCas9(1.1)	Sniper-Cas9	SpCas9-HF	evoCas9	xCas9-3.7	gTURQ.1	gTURQ.2	gTURQ.3	gTURQ.4	gEGFP.3	gI-SceI
Construct length (bp)	9215	9360	9360	9360	9215	9300	9215	9217	3046	3046	3046	3046	3046	3056
1	303.3								96.7					
2		303.3							96.7					
3			303.3						96.7					
4				303.3					96.7					
5					303.3				96.7					
6						303.3			96.7					
7							303.3		96.7					
8								303.3	96.7					
9	303.3									96.7				
10		303.3								96.7				
11			303.3							96.7				
12				303.3						96.7				
13					303.3					96.7				
14						303.3				96.7				
15							303.3			96.7				
16								303.3		96.7				
17	303.3										96.7			
18		303.3									96.7			
19			303.3								96.7			
20				303.3							96.7			
21					303.3						96.7			
22						303.3					96.7			
23							303.3				96.7			
24								303.3			96.7			

25	303.3											96.7		
26		303.3										96.7		
27			303.3									96.7		
28				303.3								96.7		
29					303.3							96.7		
30						303.3						96.7		
31							303.3					96.7		
32								303.3				96.7		
33	303.3												96.7	
34		303.3											96.7	
35			303.3										96.7	
36				303.3									96.7	
37					303.3								96.7	
38						303.3							96.7	
39							303.3						96.7	
40								303.3					96.7	
41	303.3													96.7
42		303.3												96.7
43			303.3											96.7
44				303.3										96.7
45					303.3									96.7
46						303.3								96.7
47							303.3							96.7
48								303.3						96.7

Supplementary Table S4. Transfection scheme for testing the activity of the different nucleases in H27 cells (**Supplementary Figure S1**).

H27 cells	7 ×10^4 cells per well of 24-well plates									
	500ng DNA and 1.54 µl PEI (1 mg ml^{-1}) per well (medium replaced at 6 h post-transfection)									
Encoded products	SpCas9	SpCas9-KA	SpCas9-KARA	eSpCas9(1.1)	Sniper-Cas9	SpCas9-HF	evoCas9	xCas9-3.7	gEGFP.2	gEGFP.21
Construct length (bp)	9215	9360	9360	9360	9215	9300	9215	9217	3046	3047
1	375.8								124.2	
2		375.8							124.2	
3			375.8						124.2	
4				375.8					124.2	
5					375.8				124.2	
6						375.8			124.2	
7							375.8		124.2	
8								375.8	124.2	
9	375.8									124.2
10		375.8								124.2
11			375.8							124.2
12				375.8						124.2
13					375.8					124.2
14						375.8				124.2
15							375.8			124.2
16								375.8		124.2

Supplementary Table S5. Transfection scheme for testing the activity of the different nickases in H27 cells (**Figure 2B**).

H27 cells	7 ×10^4 cells per well of 24-well plates														
	500ng DNA and 1.54 µl PEI (1 mg ml^{-1}) per well (medium replaced at 6 h post-transfection)														
Encoded products	SpCas9 ^{D10A}	SpCas9-KA ^{D10A}	SpCas9-KARA ^{D10A}	eSpCas9(1.1) ^{D10A}	Sniper-Cas9 ^{D10A}	SpCas9-HF ^{D10A}	xCas9-3.7 ^{D10A}	evoCas9 ^{D10A}	gEGFP .1	gEGFP .3	gEGFP .2	gEGFP .T2	gEGFP .21	gI-SceI	DsRed
Construct length (bp)	9215	9360	9360	9360	9215	9300	9217	9215	3046	3046	3046	3974	3047	3056	4712
1	220.0								72.7			94.9			112.5
2		220.0							72.7			94.9			112.5
3			220.0						72.7			94.9			112.5
4				220.0					72.7			94.9			112.5
5					220.0				72.7			94.9			112.5
6						220.0			72.7			94.9			112.5
7							220.0		72.7			94.9			112.5
8								220.0	72.7			94.9			112.5
9	220.0									72.7		94.9			112.5
10		220.0								72.7		94.9			112.5
11			220.0							72.7		94.9			112.5
12				220.0						72.7		94.9			112.5
13					220.0					72.7		94.9			112.5
14						220.0				72.7		94.9			112.5
15							220.0			72.7		94.9			112.5
16								220.0		72.7		94.9			112.5
17	220.0										72.7	94.9			112.5
18		220.0									72.7	94.9			112.5
19			220.0								72.7	94.9			112.5
20				220.0							72.7	94.9			112.5
21					220.0						72.7	94.9			112.5
22						220.0					72.7	94.9			112.5
23							220.0				72.7	94.9			112.5
24								220.0			72.7	94.9			112.5

25	220.0										83.8		83.8		112.5
26		220.0									83.8		83.8		112.5
27			220.0								83.8		83.8		112.5
28				220.0							83.8		83.8		112.5
29					220.0						83.8		83.8		112.5
30						220.0					83.8		83.8		112.5
31							220.0				83.8		83.8		112.5
32								220.0			83.8		83.8		112.5
33	220.0										83.8		83.8		112.5
34		220.0												167.6	112.5
35			220.0											167.6	112.5
36				220.0										167.6	112.5
37					220.0									167.6	112.5
38						220.0								167.6	112.5
39							220.0							167.6	112.5
40								220.0						167.6	112.5

Supplementary Table S6. Transfection scheme for determining the expression of the different nucleases in H27 cells (**Supplementary Figure S4A,** left panel).

H27 cells	1.5 ×10^{5} cells per well of 12-well plates								
	1050ng DNA and 3.95 µl PEI (1 mg ml^{-1}) per well (medium replaced at 6 h post-transfection)								
Encoded products	SpCas9	SpCas9-KA	SpCas9-KARA	eSpCas9(1.1)	Sniper-Cas9	SpCas9-HF	xCas9-3.6	xCas9-3.7	evoCas9
Construct length (bp)	9215	9360	9360	9360	9215	9300	9217	9217	9215
1	1050.0								
2		1050.0							
3			1050.0						
4				1050.0					
5					1050.0				
6						1050.0			
7							1050.0		
8								1050.0	
9									1050.0

Supplementary Table S7. Transfection scheme for determining the expression of the different nickases in H27 cells (**Supplementary Figure S4A,** right panel).

H27 cells	1.5 ×10^{5} cells per well of 12-well plates								
	1050ng DNA and 3.95 µl PEI (1 mg ml^{-1}) per well (medium replaced at 6 h post-transfection)								
Encoded products	SpCas9^{D10A}	SpCas9-KA $_{D10A}$	SpCas9-KARA $_{D10A}$	eSpCas9(1.1) $_{D10A}$	Sniper-Cas9 $_{D10A}$	SpCas9-HF ^{D10A}	xCas9-3.6 $_{D10A}$	xCas9-3.7 $_{D10A}$	evoCas9 ^{D10A}
Construct length (bp)	9215	9360	9360	9360	9215	9300	9217	9217	9215
1	1050.0								
2		1050.0							
3			1050.0						
4				1050.0					
5					1050.0				
6						1050.0			
7							1050.0		
8								1050.0	
9									1050.0

Supplementary Table S8. Transfection scheme for characterizing by amplicon deep sequencing footprints induced by dual nRGNs at *EGFP* (**Figure 2C**, **Supplementary Figures S2** and **S4B**).

H27 cells	1.4 ×10^5 cells per well of 12-well plates<br>1000ng DNA and 3.69 µl PEI (1 mg ml^{-1}) per well (medium replaced at 6 h post-transfection)									
Encoded products	SpCas9^{D10A}	SpCas9-KA ^{D10A}	SpCas9-KARA ^{D10A}	eSpCas9(1.1) ^{D10A}	Sniper-Cas9 ^{D10A}	SpCas9-HF ^{D10A}	xCas9-3.7 ^{D10A}	evoCas9 ^{D10A}	gEGFP.2	gEGFP.21
Construct length (bp)	9215	9360	9360	9360	9215	9300	9217	9215	3046	3047
1	567.6								199.0	199.0
2		567.6							199.0	199.0
3			567.6						199.0	199.0
4				567.6					199.0	199.0
5					567.6				199.0	199.0
6						567.6			199.0	199.0
7							567.6		199.0	199.0
8								567.6	199.0	199.0

Supplementary Table S9. Transfection scheme for characterizing by amplicon deep sequencing footprints induced by dual nRGNs at *H2AX* (**Figure 6D**, **Supplementary Figures S4B** and **S10**).

HEK293T cells	3.5 ×10^5 cells per well of 12-well plates<br>2000ng DNA and 9.21 µl PEI (1 mg ml^{-1}) per well (medium replaced at 6 h post-transfection)						
Encoded products	SpCas9^{D10A}	eSpCas9(1.1) ^{D10A}	Sniper-Cas9 ^{D10A}	gH2AX.8	gH2AX.10	gH2AX.12	gH2AX.13
Construct length (bp)	9215	9360	9215	3056	3056	3056	3056
1	1202.5			398.8			398.8
2		1202.5		398.8			398.8
3			1202.5	398.8			398.8
4	1202.5				398.8	398.8	
5		1202.5			398.8	398.8	
6			1202.5		398.8	398.8	

Supplementary Table S10. Transfection scheme for assessing dose-dependent expression of xCas9-3.7 and xCas9-3.7^{D10A} in H27 cells (**Supplementary Figure S5A**).

H27 cells	1.5 ×10^{5} cells per well of 12-well plates						
	1500ng and 3.95 µl PEI (1 mg ml^{-1}) per well (medium replaced at 6 h post-transfection)						
Encoded products	dSaCas9	SpCas9	eSpCas9(1.1)	xCas9-3.7	SpCas9^{D10A}	eSpCas9(1.1)^{D10A}	xCas9-3.7^{D10A}
Construct length (bp)	8379	9215	9360	9217	9215	9360	9217
1	1125.0	375.0					
2	1125.0		375.0				
3	1125.0			375.0			
4	750.0			750.0			
5	375.0			1125.0			
6				1500.0			
7	1125.0				375.0		
8	1125.0					375.0	
9	1125.0						375.0
10	750.0						750.0
11	375.0						1125.0
12							1500.0

Supplementary Table S11. Transfection scheme for assessing dose-dependent activity of xCas9-3.7 in TURQ2 cells (**Supplementary Figure S5B**).

TURQ2 cells	5 ×10^4 cells per well of 24-well plates							
	400ng DNA and 1.54 µl PEI (1 mg ml^{-1}) per well (medium replaced at 6 h post-transfection)							
Encoded products	SpCas9	xCas9-3.7	gTURQ.1	gTURQ.2	gTURQ.3	gTURQ.4	gEGFP.3	gI-SceI
Construct length (bp)	9215	9217	3046	3046	3046	3046	3046	3056
1	150.3		99.4					
2		150.3	99.4					
3		300.6	99.4					
4	150.3			99.4				
5		150.3		99.4				
6		300.6		99.4				
7	150.3				99.4			
8		150.3			99.4			
9		300.6			99.4			
10	150.3					99.4		
11		150.3				99.4		
12		300.6				99.4		
13	150.3						99.4	
14		150.3					99.4	
15		300.6					99.4	
16	150.3							99.4
17		150.3						99.4
18		300.6						99.4

Supplementary Table S12. Transfection scheme for assessing dose-dependent activities of xCas9-3.7^{D10A} in H27 cells (**Supplementary Figure S5C**).

H27 cells	7 ×10^{4} cells per well of 24-well plates									
	500ng DNA and 1.54 µl PEI (1 mg ml^{-1}) per well (medium replaced at 6 h post-transfection)									
Encoded products	dSaCas9	SpCas9 ^{D10A}	xCas9-3.7 ^{D10A}	gEGFP.1	gEGFP.3	gEGFP.2	gEGFP.T2	gEGFP.21	gI-SceI	DsRed
Construct length (bp)	8379	9215	9217	3046	3046	3046	3974	3047	3056	4712
1	110.0	110.0		72.7			94.9			112.5
2	110.0		110.0	72.7			94.9			112.5
3			220.0	72.7			94.9			112.5
4	110.0	110.0			72.7		94.9			112.5
5	110.0		110.0		72.7		94.9			112.5
6			220.0		72.7		94.9			112.5
7	110.0	110.0				72.7	94.9			112.5
8	110.0		110.0			72.7	94.9			112.5
9			220.0			72.7	94.9			112.5
10	110.0	110.0				83.8		83.8		112.5
11	110.0		110.0			83.8		83.8		112.5
12			220.0			83.8		83.8		112.5
13	110.0	110.0							167.6	112.5
14	110.0		110.0						167.6	112.5
15			220.0						167.6	112.5

Supplementary Table S13. Transfection scheme for testing the specificity of nickases in TURQ2 cells (**Figure 3B**).

TURQ2 cells	5 $\times 10^4$ cells per well of 24-well plates															
	500ng DNA and 1.54 µl PEI (1 mg ml^{-1}) per well (medium replaced at 6 h post-transfection)															
Encoded products	SaCas9 D10A	Sa-gRNA.G	SpCas9 D10A	SpCas9-KA D10A	SpCas9-KARA D10A	eSpCas9(1.1) D10A	Sniper-Cas9 D10A	SpCas9-HF D10A	evoCas9- D10A	xCas9-3.7 D10A	gEGFP .2	gOT-1	gOT-2	gOT-3	gOT -4	DsRed
Construct length (bp)	8379	2288	9215	9360	9360	9360	9215	9300	9215	9217	3046	3047	3046	3046	304 6	4712
1	120.6	32.9	134.7								43.9					67.8
2	120.6	32.9	134.7									43.9				67.8
3	120.6	32.9	134.7										43.9			67.8
4	120.6	32.9	134.7											43.9		67.8
5	120.6	32.9	134.7												43.9	67.8
6	120.6	32.9		134.7							43.9					67.8
7	120.6	32.9		134.7								43.9				67.8
8	120.6	32.9		134.7									43.9			67.8
9	120.6	32.9		134.7										43.9		67.8
10	120.6	32.9		134.7											43.9	67.8
11	120.6	32.9			134.7						43.9					67.8
12	120.6	32.9			134.7							43.9				67.8
13	120.6	32.9			134.7								43.9			67.8
14	120.6	32.9			134.7									43.9		67.8
15	120.6	32.9			134.7										43.9	67.8
16	120.6	32.9				134.7					43.9					67.8
17	120.6	32.9				134.7						43.9				67.8
18	120.6	32.9				134.7							43.9			67.8
19	120.6	32.9				134.7								43.9		67.8
20	120.6	32.9				134.7									43.9	67.8
21	120.6	32.9					134.7				43.9					67.8
22	120.6	32.9					134.7					43.9				67.8
23	120.6	32.9					134.7						43.9			67.8
24	120.6	32.9					134.7							43.9		67.8

25	120.6	32.9					134.7								43.9	67.8
26	120.6	32.9						134.7			43.9					67.8
27	120.6	32.9						134.7				43.9				67.8
28	120.6	32.9						134.7					43.9			67.8
29	120.6	32.9						134.7						43.9		67.8
30	120.6	32.9						134.7							43.9	67.8
31	120.6	32.9							134.7		43.9					67.8
32	120.6	32.9							134.7			43.9				67.8
33	120.6	32.9							134.7				43.9			67.8
34	120.6	32.9							134.7					43.9		67.8
35	120.6	32.9							134.7						43.9	67.8
36	120.6	32.9								134.7	43.9					67.8
37	120.6	32.9								134.7		43.9				67.8
38	120.6	32.9								134.7			43.9			67.8
39	120.6	32.9								134.7				43.9		67.8
40	120.6	32.9								134.7					43.9	67.8

Encoded products	SaCas9 D10A	Sa-gRNA.G	SpCas9 D10A	SpCas9-KA D10A	SpCas9-KARA D10A	eSpCas9(1.1) D10A	Sniper-Cas9 D10A	SpCas9-HF D10A	evoCas9- D10A	xCas9-3.7 D10A	gOT-5	gOT-6	gOT-7	gOT-8	DsRed
Construct length (bp)	8379	2288	9215	9360	9360	9360	9215	9300	9215	9217	3046	3047	3046	3046	4712
41	120.6	32.9	134.7								43.9				67.8
42	120.6	32.9	134.7									43.9			67.8
43	120.6	32.9	134.7										43.9		67.8
44	120.6	32.9	134.7											43.9	67.8
45	120.6	32.9		134.7							43.9				67.8
46	120.6	32.9		134.7								43.9			67.8
47	120.6	32.9		134.7									43.9		67.8
48	120.6	32.9		134.7										43.9	67.8
49	120.6	32.9			134.7						43.9				67.8
50	120.6	32.9			134.7							43.9			67.8
51	120.6	32.9			134.7								43.9		67.8

52	120.6	32.9			134.7									43.9	67.8
53	120.6	32.9				134.7					43.9				67.8
54	120.6	32.9				134.7						43.9			67.8
55	120.6	32.9				134.7							43.9		67.8
56	120.6	32.9				134.7								43.9	67.8
57	120.6	32.9					134.7				43.9				67.8
58	120.6	32.9					134.7					43.9			67.8
59	120.6	32.9					134.7						43.9		67.8
60	120.6	32.9					134.7							43.9	67.8
61	120.6	32.9						134.7			43.9				67.8
62	120.6	32.9						134.7				43.9			67.8
63	120.6	32.9						134.7					43.9		67.8
64	120.6	32.9						134.7						43.9	67.8
65	120.6	32.9							134.7		43.9				67.8
66	120.6	32.9							134.7			43.9			67.8
67	120.6	32.9							134.7				43.9		67.8
68	120.6	32.9							134.7					43.9	67.8
69	120.6	32.9								134.7	43.9				67.8
70	120.6	32.9								134.7		43.9			67.8
71	120.6	32.9								134.7			43.9		67.8
72	120.6	32.9								134.7				43.9	67.8

Encoded products	SaCas9 $_{D10A}$	Sa-gRNA.G	SpCas9 $_{D10A}$	SpCas9-KA ^{D10A}	SpCas9-KARA $_{D10A}$	eSpCas9(1.1) $_{D10A}$	Sniper-Cas9 $_{D10A}$	SpCas9-HF $_{D10A}$	evoCas9- $_{D10A}$	xCas9-3.7 $_{D10A}$	gOT-9	gOT-10	gI-SceI	DsRed
Construct length (bp)	8379	2288	9215	9360	9360	9360	9215	9300	9215	9217	3046	3046	3056	4712
73	120.6	32.9	134.7								43.9			67.8
74	120.6	32.9	134.7									43.9		67.8
75	120.6	32.9	134.7										43.9	67.8
76	120.6	32.9		134.7							43.9			67.8
77	120.6	32.9		134.7								43.9		67.8
78	120.6	32.9		134.7									43.9	67.8

79	120.6	32.9			134.7						43.9			67.8
80	120.6	32.9			134.7							43.9		67.8
81	120.6	32.9			134.7								43.9	67.8
82	120.6	32.9				134.7					43.9			67.8
83	120.6	32.9				134.7						43.9		67.8
84	120.6	32.9				134.7							43.9	67.8
85	120.6	32.9					134.7				43.9			67.8
86	120.6	32.9					134.7					43.9		67.8
87	120.6	32.9					134.7						43.9	67.8
89	120.6	32.9						134.7			43.9			67.8
90	120.6	32.9						134.7				43.9		67.8
91	120.6	32.9						134.7					43.9	67.8
92	120.6	32.9							134.7		43.9			67.8
93	120.6	32.9							134.7			43.9		67.8
94	120.6	32.9							134.7				43.9	67.8
95	120.6	32.9								134.7	43.9			67.8
96	120.6	32.9								134.7		43.9		67.8
97	120.6	32.9								134.7			43.9	67.8

Supplementary Table S14. Transfection scheme for testing the specificity of nickase complexes in H27 cells (**Supplementary Figure S8B**).

H27 cells	7 ×10^4 cells per well of 24-well plates																
	500ng DNA and 1.84 µl PEI (1 mg ml^{-1}) per well (medium replaced at 6 h post-transfection)																
Encoded products	SpCas9 D10A	eSpCas9(1.1) D10A	Sniper-Cas9 D10A	gEGFP.21	gEGFP.2	gOT-1	gOT-2	gOT-3	gOT-4	gOT-5	gOT-6	gOT-7	gOT-8	gOT-9	gOT-10	gI-SceI	DsRed
Construct length (bp)	9215	9360	9215	3047	3046	3047	3046	3046	3046	3046	3047	3046	3046	3046	3046	3056	4712
1	230.1			76.1	76.1												117.7
2		230.1		76.1	76.1												117.7
3			230.1	76.1	76.1												117.7
4	230.1			76.1		76.1											117.7
5		230.1		76.1		76.1											117.7
6			230.1	76.1		76.1											117.7
7	230.1			76.1			76.1										117.7
8		230.1		76.1			76.1										117.7
9			230.1	76.1			76.1										117.7
10	230.1			76.1				76.1									117.7
11		230.1		76.1				76.1									117.7
12			230.1	76.1				76.1									117.7
13	230.1			76.1					76.1								117.7
14		230.1		76.1					76.1								117.7
15			230.1	76.1					76.1								117.7
16	230.1			76.1						76.1							117.7
17		230.1		76.1						76.1							117.7
18			230.1	76.1						76.1							117.7
19	230.1			76.1							76.1						117.7
20		230.1		76.1							76.1						117.7
21			230.1	76.1							76.1						117.7
22	230.1			76.1								76.1					117.7
23		230.1		76.1								76.1					117.7
24			230.1	76.1								76.1					117.7

25	230.1			76.1									76.1				117.7
26		230.1		76.1									76.1				117.7
27			230.1	76.1									76.1				117.7
28	230.1			76.1										76.1			117.7
29		230.1		76.1										76.1			117.7
30			230.1	76.1										76.1			117.7
31	230.1			76.1											76.1		117.7
32		230.1		76.1											76.1		117.7
33			230.1	76.1											76.1		117.7
34	230.1			76.1												76.1	117.7
35		230.1		76.1												76.1	117.7
36			230.1	76.1												76.1	117.7

Supplementary Table S15. Transfection scheme for testing the activity of nuclease complexes with truncated gRNAs in H27 cells (**Figure 4A**).

H27 cells	7 ×10^4 cells per well of 24-well plates												
	500ng DNA and 1.54 µl PEI (1 mg ml^{-1}) per well (medium replaced at 6 h post-transfection)												
Encoded products	SpCas9	SpCas9-KA	SpCas9-KARA	eSpCas9(1.1)	Sniper-Cas9	SpCas9-HF	evoCas9	gEGFP6. tru17	gEGFP6. tru19	gEGFP6. FL20	gEGFP.7	gI-SceI	DsRed
Construct length (bp)	9215	9360	9360	9360	9215	9300	9215	3043	3045	3046	3046	3056	4712
1	230.2							76.0				76.1	117.6
2		230.2						76.0				76.1	117.6
3			230.2					76.0				76.1	117.6
4				230.2				76.0				76.1	117.6
5					230.2			76.0				76.1	117.6
6						230.2		76.0				76.1	117.6
7							230.2	76.0				76.1	117.6
8	230.2								76.0			76.1	117.6
9		230.2							76.0			76.1	117.6
10			230.2						76.0			76.1	117.6
11				230.2					76.0			76.1	117.6
12					230.2				76.0			76.1	117.6
13						230.2			76.0			76.1	117.6
14							230.2		76.0			76.1	117.6
15	230.2									76.0			117.6
16		230.2								76.0			117.6
17			230.2							76.0			117.6
18				230.2						76.0			117.6
19					230.2					76.0			117.6
20						230.2				76.0			117.6
21							230.2			76.0			117.6
22	230.2										76.0		117.6
23		230.2									76.0		117.6
24			230.2								76.0		117.6

25				230.2							76.0		117.6
26					230.2						76.0		117.6
27						230.2					76.0		117.6
28							230.2				76.0		117.6
29	230.2											152.2	117.6
30		230.2										152.2	117.6
31			230.2									152.2	117.6
32				230.2								152.2	117.6
33					230.2							152.2	117.6
34						230.2						152.2	117.6
35							230.2					152.2	117.6

Supplementary Table S16. Transfection scheme for testing the activity of nickase complexes with truncated gRNAs in H27 cells (**Figure 4A**).

H27 cells	7×10^4 cells per well of 24-well plates												
	500ng DNA and 1.54 µl PEI (1 mg ml^{-1}) per well (medium replaced at 6 h post-transfection)												
Encoded products	SpCas9 ^{D10A}	SpCas9-KA ^{D10A}	SpCas9-KARA ^{D10A}	eSpCas9(1.1) ^{D10A}	Sniper-Cas9 ^{D10A}	SpCas9-HF ^{D10A}	evoCas9 ^{D10A}	gEGFP6.tru17	gEGFP6.tru19	gEGFP6.FL20	gEGFP.7	gI-SceI	DsRed
Construct length (bp)	9215	9360	9360	9360	9215	9300	9215	3043	3045	3046	3046	3056	4712
1	230.2							76.0			76.1		117.7
2		230.2						76.0			76.1		117.7
3			230.2					76.0			76.1		117.7
4				230.2				76.0			76.1		117.7
5					230.2			76.0			76.1		117.7
6						230.2		76.0			76.1		117.7
7							230.2	76.0			76.1		117.7
8	230.2								76.0		76.1		117.7
9		230.2							76.0		76.1		117.7
10			230.2						76.0		76.1		117.7
11				230.2					76.0		76.1		117.7
12					230.2				76.0		76.1		117.7
13						230.2			76.0		76.1		117.7
14							230.2		76.0		76.1		117.7
15	230.2									76.0	76.1		117.7
16		230.2								76.0	76.1		117.7
17			230.2							76.0	76.1		117.7
18				230.2						76.0	76.1		117.7
19					230.2					76.0	76.1		117.7
20						230.2				76.0	76.1		117.7
21							230.2			76.0	76.1		117.7
22	230.2											152.1	117.7
23		230.2										152.1	117.7
24			230.2									152.1	117.7

25				230.2								152.1	117.7
26					230.2							152.1	117.7
27						230.2						152.1	117.7
28							230.2					152.1	117.7

Supplementary Table S17. Transfection scheme for testing the activity of nickase complexes with truncated gRNAs in TURQ2 cells (**Figure 4C**).

TURQ2 cells	5×10^4 cells per well of 24-well plates									
	400ng DNA and 1.54 µl PEI (1 mg ml^{-1}) per well (medium replaced at 6 h post-transfection)									
Encoded products	$SaCas9^{D10A}$	Sa-gRNA.G	$SpCas9^{D10A}$	Sniper-Cas9 ^{D10A}	gEGFP27a.tru .17	gEGFP28. tru.18	gEGFP29. tru.19	gEGFP30.FL. 20	gI-SceI	DsRed
Construct length (bp)	8379	2288	9215	9215	3053	3054	3055	3056	3056	4712
1	121.2	33.1	133.3		44.2					68.2
2	121.2	33.1		133.3	44.2					68.2
3	121.2	33.1	133.3			44.2				68.2
4	121.2	33.1		133.3		44.2				68.2
5	121.2	33.1	133.3				44.2			68.2
6	121.2	33.1		133.3			44.2			68.2
7	121.2	33.1	133.3					44.2		68.2
8	121.2	33.1		133.3				44.2		68.2
9	121.2	33.1	133.3						44.2	68.2
10	121.2	33.1		133.3					44.2	68.2

Supplementary Table S18. Transfection scheme for testing the specificity of nickase complexes with truncated gRNAs in TURQ2 cells (**Figure 4D**).

TURQ2 cells	5 ×10^4 cells per well of 24-well plates								
	400ng DNA and 1.54 µl PEI (1 mg ml^{-1}) per well (medium replaced at 6 h post-transfection)								
Encoded products	SaCas9^{D10A}	Sa-gRNA.G	SpCas9^{D10A}	Sniper-Cas9 ^{D10A}	gEGFP28. tru.18	gEGFP28. tru.18.OT1	gEGFP28. tru.18.0T2	gI-SceI	DsRed
Construct length (bp)	8379	2288	9215	9215	3053	3054	3055	3056	4712
1	121.2	33.1	133.3		44.2				68.2
2	121.2	33.1		133.3	44.2				68.2
3	121.2	33.1	133.3			44.2			68.2
4	121.2	33.1		133.3		44.2			68.2
5	121.2	33.1	133.3				44.2		68.2
6	121.2	33.1		133.3			44.2		68.2
7	121.2	33.1	133.3						68.2
8	121.2	33.1		133.3					68.2
9	121.2	33.1	133.3					44.2	68.2
10	121.2	33.1		133.3				44.2	68.2

Supplementary Table S19. Transfection scheme for determining the impact of alternate chromatin states on nickase complex activities in HEK.EGFP$^{TetO.KRAB}$ cells (**Figure 5**).

HEK.EGFP$^{TetO.KRAB}$ cells	2.5 ×10^{5} cells per well of 24-well plates									
	1050ng DNA and 4.61 µl PEI (1 mg ml^{-1}) per well (medium replaced at 6 h post-transfection)									
Encoded products	SpCas9^{D10A}	eSpCas9(1.1) ^{D10A}	Sniper-Cas9 ^{D10A}	gEGFP.1	gEGFP.3	gEGFP.2	gEGFP.T2	gEGFP.21	gI-SceI	DsRed
Construct length (bp)	9215	9360	9215	3046	3046	3046	3974	3047	3056	4712
1	461.9			152.7			199.2			236.2
2	461.9				152.7		199.2			236.2
3	461.9					152.7	199.2			236.2
4	461.9					175.9		176.0		236.2
5	461.9								351.9	236.2
6		461.9		152.7			199.2			236.2
7		461.9			152.7		199.2			236.2
8		461.9				152.7	199.2			236.2
9		461.9				175.9		176.0		236.2
10		461.9							351.9	236.2
11			461.9	152.7			199.2			236.2
12			461.9		152.7		199.2			236.2
13			461.9			152.7	199.2			236.2
14			461.9			175.9		176.0		236.2
15			461.9						351.9	236.2

Supplementary Table S20. Transfection scheme for testing nuclease complex activities in H2AX::mCherry$^+$ HeLa cells (**Figure 6C, left panel**).

H2AX::mCherry$^+$ HeLa cells	6 $\times 10^4$ cells per well of 24-well plates												
	450ng DNA and 1.58 µl PEI (1 mg ml^{-1}) per well (medium replaced at 6 h post-transfection)												
Encoded products	SpCas9	eSpCas9(1.1)	Sniper-Cas9	gH2AX.8	gH2AX.9	gH2AX.10	gH2AX.11	gH2AX.12	gH2AX.13	gH2AX.14	gH2AX.15	gI-SceI	EGFP
Construct length (bp)	9215	9360	9215	3056	3056	3056	3056	3056	3056	3057	3056	3056	5763
1	229.9			76.3									117.7
2		229.9		76.3									117.7
3			229.9	76.3									117.7
4	229.9				76.3								117.7
5		229.9			76.3								117.7
6			229.9		76.3								117.7
7	229.9					76.3							117.7
8		229.9				76.3							117.7
9			229.9			76.3							117.7
10	229.9						76.3						117.7
11		229.9					76.3						117.7
12			229.9				76.3						117.7
13	229.9							76.3					117.7
14		229.9						76.3					117.7
15			229.9					76.3					117.7
16	229.9								76.3				117.7
17		229.9							76.3				117.7
18			229.9						76.3				117.7
19	229.9									76.3			117.7
20		229.9								76.3			117.7
21			229.9							76.3			117.7
22	229.9										76.3		117.7
23		229.9									76.3		117.7
24			229.9								76.3		117.7

25	229.9											76.3	117.7
26		229.9										76.3	117.7
27			229.9									76.3	117.7

Supplementary Table S21. Transfection scheme for testing nickase complex activities in H2AX::mCherry+ HeLa cells (**Figure 6C, right panel**).

H2AX::mCherry+ HeLa cells	6 ×10^4 cells per well of 24-well plates												
	450ng DNA and 1.58 µl PEI (1 mg ml^{-1}) per well (medium replaced at 6 h post-transfection)												
Encoded products	SpCas9 D10A	eSpCas9(1.1) D10A	Sniper-Cas9 D10A	gH2AX.8	gH2AX.9	gH2AX.10	gH2AX.11	gH2AX.12	gH2AX.13	gH2AX.14	gH2AX.15	gI-SceI	EGFP
Construct length (bp)	9215	9360	9215	3056	3056	3056	3056	3056	3056	3057	3056	3056	5763
1	196.6			65.2	65.2								123.0
2		196.6		65.2	65.2								123.0
3			196.6	65.2	65.2								123.0
4	196.6			65.2			65.2						123.0
5		196.6		65.2			65.2						123.0
6			196.6	65.2			65.2						123.0
7	196.6			65.2				65.2					123.0
8		196.6		65.2				65.2					123.0
9			196.6	65.2				65.2					123.0
10	196.6			65.2					65.2				123.0
11		196.6		65.2					65.2				123.0
12			196.6	65.2					65.2				123.0
13	196.6					65.2	65.2						123.0
14		196.6				65.2	65.2						123.0
15			196.6			65.2	65.2						123.0
16	196.6					65.2		65.2					123.0
17		196.6				65.2		65.2					123.0
18			196.6			65.2		65.2					123.0
19	196.6					65.2			65.2				123.0

20		196.6				65.2			65.2				123.0
21			196.6			65.2			65.2				123.0
22	196.6					65.2				65.2			123.0
23		196.6				65.2				65.2			123.0
24			196.6			65.2				65.2			123.0
25	196.6					65.2					65.2		123.0
26		196.6				65.2					65.2		123.0
27			196.6			65.2					65.2		123.0
28	196.6											130.4	123.0
29		196.6										130.4	123.0
30			196.6									130.4	123.0

Supplementary Table S22. Transfection scheme for testing nickase complex specificities in H2AX::mCherry^{+} HeLa cells (**Figure 7, upper panel**).

H2AX::mCherry^{+} HeLa cells	6 ×10^{4} cells per well of 24-well plates							
	450ng DNA and 1.58 µl PEI (1 mg ml^{-1}) per well (medium replaced at 6 h post-transfection)							
Encoded products	SpCas9^{D10A}	eSpCas9(1.1) ^{D10A}	Sniper-Cas9 ^{D10A}	gH2AX.OT2	gH2AX.OT3	gH2AX.OT4	gI-SceI	EGFP
Construct length (bp)	9215	9360	9215	3056	3056	3057	3056	5763
1	196.6			65.2	65.2			123.0
2		196.6		65.2	65.2			123.0
3			196.6	65.2	65.2			123.0
4	196.6			65.2		65.2		123.0
5		196.6		65.2		65.2		123.0
6			196.6	65.2		65.2		123.0
7	196.6						130.4	123.0
8		196.6					130.4	123.0
9			196.6				130.4	123.0

Supplementary Table S23. Transfection scheme for testing nickase complex specificities in H2AX::mCherry+ HeLa cells (**Figure 7, lower panel**).

H2AX::mCherry+ HeLa cells	6×10^4 cells per well of 24-well plates											
	450ng DNA and 1.58µl PEI (1 mg ml^{-1}) per well (medium replaced at 6 h post-transfection)											
Encoded products	$SpCas9^{D10A}$	$eSpCas9(1.1)^{D10A}$	Sniper-$Cas9^{D10A}$	gH2AX.8	gH2AX.11	gH2AX.12	gH2AX.15	gH2AX. OT1	gH2AX. OT2	gH2AX. OT3	gI-SceI	EGFP
Construct length (bp)	9215	9360	9215	3056	3056	3056	3057	3056	3056	3056	3056	5763
1	196.6				65.2				65.2			123.0
2		196.6			65.2				65.2			123.0
3			196.6		65.2				65.2			123.0
4	196.6					65.2			65.2			123.0
5		196.6				65.2			65.2			123.0
6			196.6			65.2			65.2			123.0
7	196.6			65.2				65.2				123.0
8		196.6		65.2				65.2				123.0
9			196.6	65.2				65.2				123.0
10	196.6			65.2						65.2		123.0
11		196.6		65.2						65.2		123.0
12			196.6	65.2						65.2		123.0
13	196.6						65.2		65.2			123.0
14		196.6					65.2		65.2			123.0
15			196.6				65.2		65.2			123.0
16	196.6										65.2	123.0
17		196.6									65.2	123.0
18			196.6								65.2	123.0
19	196.6										130.4	123.0
20		196.6									130.4	123.0
21			196.6								130.4	123.0

Supplementary Table S24. Transfection scheme of *OCT4* gene targeting experiments in HeLa cells (**Figure 9**).

HeLa cells	5 ×10^4 cells per well of 24-well plates									
	500ng DNA and 1.54 µl PEI (1 mg ml^{-1}) per well (medium replaced at 6 h post-transfection)									
Encoded products	SpCas9^{D10A}	eSpCas9(1.1) ^{D10A}	pDonor^{OCT4}	gOCT4.1	gOCT4.2	gOCT4.Sp1	gOCT4.Sp2	gOCT4.Sp3	gOCT4.Sp4	gI-SceI
Construct length (bp)	9215	9360	7955	3046	3046	3056	3056	3056	3056	3056
1	198.1		171.0	65.5		65.5				
2	198.1		171.0	65.5			65.5			
3	198.1		171.0			65.5		65.5		
4	198.1		171.0			65.5			65.5	
5	198.1		171.0				65.5	65.5		
6	198.1		171.0				65.5		65.5	
7	198.1		171.0		65.5			65.5		
8	198.1		171.0		65.5				65.5	
9	198.1		171.0							131.0
10		198.1	171.0	65.5		65.5				
11		198.1	171.0	65.5			65.5			
12		198.1	171.0			65.5		65.5		
13		198.1	171.0			65.5			65.5	
14		198.1	171.0				65.5	65.5		
15		198.1	171.0				65.5		65.5	
16		198.1	171.0		65.5			65.5		
17		198.1	171.0		65.5				65.5	
18		198.1	171.0							131.0

Supplementary Table S25. Transfection scheme used for validating the gRNA construct used in the orthogonal HTGTS assays (**Supplementary Figure S13A**).

HEK293T cells	1.8×10^5 cells per well of 24-well plates			
	1000ng and 3.95 µl PEI (1 mg ml^{-1}) per well (medium replaced at 6 h post-transfection)			
Encoded products	SpCas9	eSpCas9(1.1)	Sniper-Cas9	gVEGFA
Construct length (bp)	9215	9360	9215	3056
1	751.0			249.0
2		751.0		249.0
3			751.0	249.0

Supplementary Table S26. Transfection scheme for generating genomic DNA samples for orthogonal HTGTS analyses (**Figures 12** and **13, Supplementary Figure S13B**).

HEK293T cells	2.5×10^6 cells per well of T25 flask						
	4290 ng DNA and 12.86 µl PEI (1 mg ml^{-1}) per well (medium replaced at 16 h post-transfection)						
Encoded products	SaCas9	SagRNA$^{RAG1.1}$	SpCas9	SpCas9^{D10A}	eSpCas9(1.1)	eSpCas9(1.1)^{D10A}	gVEGFA
Construct length (bp)	8379	2288	9215	9215	9360	9360	3056
1	3370	920					
2	1570	430	1720				570
3	1570	430		1720			570
4	1570	430			1720		570
5	1570	430				1720	570

Supplementary Table S27. Transfection scheme of *OCT4* gene targeting experiments in iPSCs (**Figure 10**).

iPSCs	5 ×10^4 cells per well of 24-well plates							
	500ng DNA and 1.50 µl Lipofectamine Stem per well (medium replaced at 24 h post-transfection)							
Encoded products	SpCas9	SpCas9^{D10A}	eSpCas9(1.1)	eSpCas9(1.1) ^{D10A}	pDonor^{OCT4}	gOCT4.2	gOCT4.Sp4	gI-SceI
Construct length (bp)	9215	9215	9360	9360	7955	3047	3056	3056
1	198.1				171.0	65.5		65.5
2	198.1				171.0		65.5	65.5
3		198.1			171.0	65.5	65.5	
4		198.1			171.0			131.0
5			198.1		171.0	65.5		65.5
6			198.1		171.0		65.5	65.5
7				198.1	171.0	65.5	65.5	
8				198.1	171.0			131.0

Supplementary Table S28. Primer pairs and compositions of the PCR mixtures used for *POU5F1P4* and *POU5F1P5* off-target analyses in genome-modified HeLa and iPSC populations.

Target	Primer code	Primers (5' → 3') / final concentrations (µM)	dNTP (mM)	$MgCl_2$ (mM)	GoTaq Flexi Buffer	GoTaq (Units)	Amplicon size (bp)
POU5F1P4	#1581	AGAAGGATGTGGTCCGAGTG / 0.5	0.4	1	1×	2.5	765
	#1583	TTACAGGTGTGAGCTACGGC / 0.5					
POU5F1P5	#1586	TCAAGCCTCTTAACCCCTGC / 0.5	0.4	1	1×	2.5	724
	#1587	CTAGAAGGATGTGATCCGAGTGTG / 0.5					

Supplementary Table S29. Primer pairs used for amplifying the orthogonal HTGTS prey and bait target sites.

Target	Primer code	Primers (5’ → 3’)	Amplicon size (bp)
VEGFA Prey site	#7370	CTCTCTGTACATGAAGCAACTCCAG	393
	#7371	CCGTTCCCTCTTTGCTAGGAATATTG	
RAG1 Bait site	#1361	GCACCTAATGTATACTGGGAC	593
	#1363	TAACAATGGCTGAGTTGGGAC	

Supplementary Table S30. PCR cycling parameters used for *POU5F1P4* and *POU5F1P5* off-target analyses in genome-modified HeLa and iPSC populations.

Target	Initial denaturation	Denaturation	Annealing	elongation	Cycles	Final elongation
POU5F1P4	95 ℃	95 ℃	65.7 ℃	72 ℃	40	72 ℃
	5 min	30 sec	30 sec	1 min		2 min
POU5F1P5	95 ℃	95 ℃	55 ℃	72 ℃	35	72 ℃
	5 min	30 sec	30 sec	1 min		2 min

Supplementary Table S31. PCR cycling parameters used for amplifying the orthogonal HTGTS prey and bait target sites.

Target	Initial denaturation	Denaturation	Annealing	Elongation	Cycles	Final elongation
VEGFA Prey site	95 ℃	95 ℃	60.9 ℃	72 ℃	40	72 ℃
	5 min	30 sec	30 sec	30 sec		5 min
RAG1 Bait site	95 ℃	95 ℃	60 ℃	70 ℃	33	70 ℃
	2 min	20 sec	10 sec	15 sec		5 min

Supplementary Table S32. Composition of PCR mixtures used for amplifying the *RAG1* target site.

Component	Volume	Final Concentration
KOD Hot Start DNA Polymerase 10× Buffer	5 µl	1×
25 mM $MgSO_4$	3 µl	1.5 mM
dNTPs (2 mM each)	5 µl	0.2 mM (each)
PCR Grade Water	31 µl	-
Sense primer (10 µM)	1.5 µl	0.3 µM
Anti-sense primer (10 µM)	1.5 µl	0.3 µM
Template DNA	2 µl	-
KOD Hot Start DNA Polymerase (1 U/µl)	1 µl	0.02 U/µl
Total reaction volume	50 µl	-

Supplementary Table S33. Composition of the PCR mixtures used for amplifying the *VEGFA* target site.

Component	Volume	Final Concentration
GoTaq G2 Flexi DNA Pol 5× Colorless Buffer	10 µl	1×
25 mM $MgCl_2$	2 µl	1.0 mM
dNTPs (2.5 mM each)	2 µl	0.1 mM (each)
PCR Grade Water	29.5 µl	-
Sense primer (10 µM)	2 µl	0.5 µM
Anti-sense primer (10 µM)	2 µl	0.5 µM
Template DNA	2 µl	-
GoTaq G2 Flexi DNA Polymerase (5U/µl)	0.5 µl	0.05 U/µl
Total reaction volume	50 µl	-

Supplementary Table S34. Thermocycler program for generating heteroduplex substrates for the T7EI-based genotyping assays.

Temperature	Time
95 °C	10 min
95 °C to 85 °C	-2.0 °C/s
85 °C	1 min
85 °C to 75 °C	-0.3 °C/s
75 °C	1 min
75 °C to 65 °C	-0.3 °C/s
65 °C	1 min
65 °C to 55 °C	-0.3 °C/s
55 °C	1 min
55 °C to 45 °C	-0.3 °C/s
45 °C	1 min
45 °C to 35 °C	-0.3 °C/s
35 °C	1 min
35 °C to 25 °C	-0.3 °C/s
25 °C	1 min
16 °C	Hold

Source: SURVEYOR Mutation Detection Kit (Transgenomic)

Supplementary Table S35. Primer pairs and PCR mixture compositions used for assessing the specificity of donor DNA insertion in *OCT4* gene editing experiments (**Figure 9D**).

Target	Primer code	Primers (5' → 3') / final concentrations (μM)	dNTP (mM)	$MgCl_2$ (mM)	GoTaq Flexi Buffer	GoTaq (Units)	Amplicon size (bp)
EGFP Ctrl	#978	GAGCTGGACGGCGACGTAAACG / 0.5	0.4	1	1×	2.5	596
	#979	CGCTTCTCGTTGGGGTCTTTGCT / 0.5					
Pseudogene junctions	#1665	TGAACTTGTGGCCGTTTACG / 0.5	0.4	1	1×	2.5	1102
	#1667	AGAAGTGGGTGGAGGAAGC / 0.5					

Supplementary Table S36. PCR cycling parameters used for assessing the specificity of donor DNA insertion in *OCT4* gene editing experiments (**Figure 9D**).

Target	Initial denaturation	Denaturation	Annealing	elongation	Cycles	Final elongation
EGFP Ctrl	98 ℃	98 ℃	72 ℃		40	72 ℃
	5 min	5 sec	20 sec			1 min
Pseudogene junctions	98 ℃	98 ℃	72 ℃		35	72 ℃
	30 sec	5 sec	10 sec			2 min

Supplementary Table S37. Overview of the antibodies used in the confocal microscopy analyses (**Figures 11C** and **11D**).

Experiments	Primary antibodies	Secondary antibodies
OCT4 gene editing (iPSCs)	1. Anti-OCT3/4 antibody (1:500; Santa Cruz Biotechnology; Cat. Nr.: SC-5279)	1. Donkey anti-mouse.IgG Alexa Fluor 555 (1:500; Invitrogen; Cat. Nr.: A31570)
Differentiation of *OCT4*-edited iPSC population	1. Anti-α Smooth Muscle Actin (α-SMA) antibody (1:500; Sigma-Aldrich; Cat. Nr.: A2547)<br>2. Anti-Tubulin β 3 (TUBB3) antibody (1:400; Eurogentec; Cat. Nr.:MMS-435P)<br>3. Anti Alpha-1 Fetoprotein (AFP) antibody (1:25; Quartett; Cat. Nr.: 2011200530)	1. Donkey anti-mouse.IgG Alexa Fluor 555 (1:500; Invitrogen; Cat. Nr.: A31570).<br>2. Goat anti-rabbit IgG Alexa Fluor 568 (1:500; Invitrogen; Cat. Nr.: A11036).

Supplementary Table S38. Composition of PCR mixtures used in gene-specific amplifications for deep sequence analyses.

Component	Volume	Final Concentration
5X Phusion HF Buffer	4 µl	1×
dNTPs (2.5 mM each)	1.6 µl	0.2 mM (each)
PCR Grade Water	10.7 µl	-
Forward primer (10 µM)	1 µl	0.5 µM
Reverse primer (10 µM)	1 µl	0.5 µM
gDNA	1.5 µl	-
Phusion DNA Polymerase (2 U/µl)	0.2 µl	0.02 U/µl
Total reaction volume	20 µl	-

Supplementary Table S39. Gene-specific primer sequences and concentrations used in the amplicon deep sequence analyses.

Target	Primer code	Primers (5’ → 3’) / final concentrations (µM)
EGFP	#1791	GATGTGTATAAGAGACAGGCACGACTTCTTCAAGTCCG / 0.5
	#1792	CGTGTGCTCTTCCGATCTAGTTCACCTTGATGCCGTTC / 0.5
H2AX	#1795	GATGTGTATAAGAGACAGTCTTCTTGGGCAGCAGCACG / 0.5
	#1796	CGTGTGCTCTTCCGATCTTGCTGCGGAAGGGCCACTAC / 0.5

Supplementary Table S40. PCR cycling parameters used in the amplicon deep sequence analyses.

Target	Initial denaturation	Denaturation	Annealing	elongation	Cycles	Final elongation
EGFP (gene-specific PCR)	98 ℃	98 ℃	66.1 ℃	72 ℃	35	72 ℃
	30 sec	10 sec	10 sec	10 sec		5 min
H2AX (gene-specific PCR)	98 ℃	98 ℃	70.0 ℃	72 ℃	35	72 ℃
	30 sec	10 sec	10 sec	10 sec		5 min
Barcode PCR	98 ℃	98 ℃	62.0 ℃	72 ℃	10	72 ℃
	30 sec	10 sec	10 sec	10 sec		3 min

Supplementary Table S41. Barcode PCR primers used in the amplicon deep sequence analyses.

Primer code	Primers (5’ → 3’)
Fun-i501	AATGATACGGCGACCACCGAGATCTACACTAGATCGCTCGTCGGCAGCGTCAGATGTGTATAAGAGACAG
Fun-i502	AATGATACGGCGACCACCGAGATCTACACCTCTCTATTCGTCGGCAGCGTCAGATGTGTATAAGAGACAG
Fun-i503	AATGATACGGCGACCACCGAGATCTACACTATCCTCTTCGTCGGCAGCGTCAGATGTGTATAAGAGACAG
Fun-i701	CAAGCAGAAGACGGCATACGAGATTCGCCTTAGTGACTGGAGTTCAGACGTGTGCTCTTCCGATCT
Fun-i702	CAAGCAGAAGACGGCATACGAGATCTAGTACGGTGACTGGAGTTCAGACGTGTGCTCTTCCGATCT
Fun-i703	CAAGCAGAAGACGGCATACGAGATTTCTGCCTGTGACTGGAGTTCAGACGTGTGCTCTTCCGATCT
Fun-i704	CAAGCAGAAGACGGCATACGAGATGCTCAGGAGTGACTGGAGTTCAGACGTGTGCTCTTCCGATCT
Fun-i705	CAAGCAGAAGACGGCATACGAGATAGGAGTCCGTGACTGGAGTTCAGACGTGTGCTCTTCCGATCT
Fun-i706	CAAGCAGAAGACGGCATACGAGATCATGCCTAGTGACTGGAGTTCAGACGTGTGCTCTTCCGATCT
Fun-i707	CAAGCAGAAGACGGCATACGAGATGTAGAGAGGTGACTGGAGTTCAGACGTGTGCTCTTCCGATCT

Supplementary Table S42. Composition of PCR mixtures used for barcode PCR amplification.

Component	Volume	Final Concentration
5X Phusion HF Buffer	4 µl	1×
dNTPs (2.5 mM each)	1.2 µl	0.15 mM (each)
PCR Grade Water	11.6 µl	-
Index primer p5-XX (5 µM)	1 µl	0.25 µM
Index primer p7-XX (5 µM)	1 µl	0.25 µM
Purified PCR product	1 µl	-
Phusion DNA Polymerase (2 U/µl)	0.2 µl	0.02 U/µl
Total reaction volume	20 µl	-
